# Modular synthesis of thiazoline and thiazole derivatives by using a cascade protocol†
†Electronic supplementary information (ESI) available. See DOI: 10.1039/c7ra05993k


**DOI:** 10.1039/c7ra05993k

**Published:** 2017-06-26

**Authors:** Zakeyah A. Alsharif, Mohammad A. Alam

**Affiliations:** a Department of Chemistry and Physics, College of Science and Mathematics, Arkansas State University, Jonesboro, AR 72467, USA. Email: malam@astate.edu; Tel: +1-870-973-3319

## Abstract

Thiazolines and thiazoles are an integral part of numerous natural products, a number of drugs, and many useful molecules such as ligands for metal catalysis. We report the first common synthetic protocol for the synthesis of thiazoles and thiazolines. Novel molecules are efficiently synthesized by using readily available and inexpensive substrates. The reaction conditions are mild and pure products are obtained without work-up and column purification.

## Abstract 2

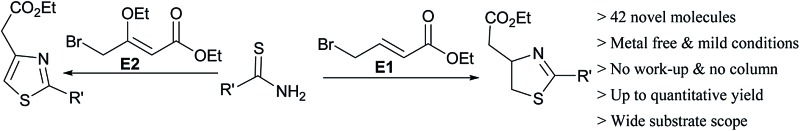
The first common synthetic protocol for thiazolines and thiazoles.

## Introduction

Dihydro-1,3-thiazole (thiazoline) and thiazole derivatives are known as drugs, natural products, and pharmacologically active synthetic molecules.^1^ Some of the approved drugs are dasatinib (anticancer), ritonavir (anti-HIV), nizatidine (anti-ulcer), and fentiazac (anti-inflammatory), among several medicines.^2^ Natural products containing both or one of the thiazoline and thiazole moieties include apratoxins, firefly luciferin, dolastatin E, mirabazole, tantazoles, piscibactin, *etc.* These molecules show a wide range of biological activities such as anticancer, antimicrobial, antimalarial, anti-tuberculosis, neurotoxic, and many other useful properties.^3^ A large number of synthetic derivatives have been reported for their wide pharmacological applications.^3^,^4^ Thiazoline derivatives also received recent attention in organic synthesis and asymmetric catalysis as ligands.^5^ Thiazole based efficient solar cells, organic semiconductors and electronics have also been receiving attention in recent years.^6^,^7^

## Results and discussion

**Scheme 1:**
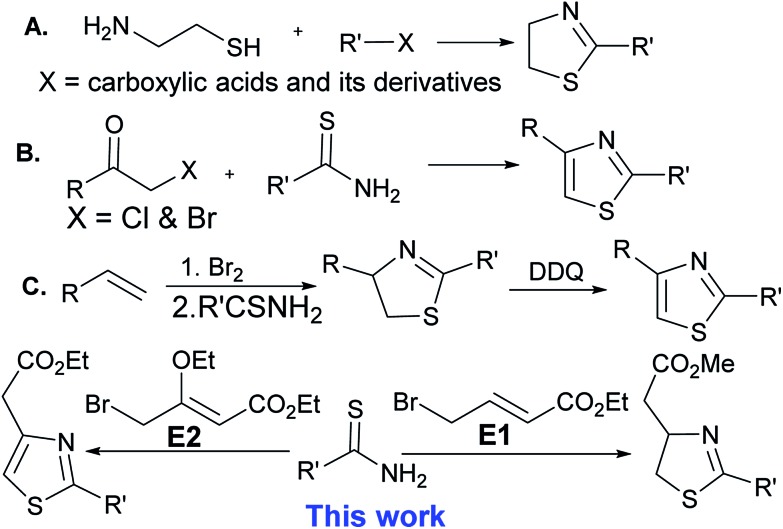
Synthesis of thiazoline and thiazole derivatives.

Because of the great value of thiazoline and thiazole derivatives, several groups have reported the synthesis of thiazolines over the past few decades, but the methods are limited to the condensation reaction of cysteamine and cysteine with carboxylic acids and its derivatives (entry A).^8^ Thiazoles are mostly synthesized by the condensation of α-haloketones with thioamides, the Hantzsch thiazole synthesis (entry B).^2^ Recently, Li *et al.* have reported the synthesis of thiazolines by reacting alkenes with bromine followed by the reaction of thioamides in a one pot reaction. Thiazoline derivative has also been oxidized with DDQ (2,3-dichloro-5,6-dicyanobenzoquinone) to form thiazoles (entry C).^9^ In a continuation of our efforts to get novel small molecule heterocycles^10^,^11^ by using domino protocol,^12^ we report the synthesis of thiazoline and thiazole derivatives using a domino protocol, mild reaction conditions, and readily available substrates (Scheme 1).

HFIP is one of the fluorinated solvents with unique properties such as high hydrogen bonding donor ability, low nucleophilicity, high ionizing power, and the ability to solvate water. HFIP is used to promote a wide range of reactions and this promoter and solvent helps to avoid metal catalyst and added reagents. HFIP mediated products are easily isolated in high yield. HFIP is an environmentally benign solvent as it can be recovered and recycled and also most of the reactions do not need work-up and tidy purification.^13^ Due to unique properties and environmentally benign nature, HFIP has been used as solvent and promoter for a wide range of reactions.^14^–^16^ We have found HFIP as the best solvent and promoter for the synthesis of pyridopyrimidinones.^12^ Keeping in mind the benign nature of HFIP, this solvent has been used as a promoter and the reaction medium for this domino reaction. In this methodology, work-up and column purification are not required. Pure products are obtained by filtration followed by recrystallization with ethyl acetate or ether. HFIP (bp = 58.2 °C) is easily recovered by distillation and recycled.

Based on our experience with HFIP,^12^ we tried the reaction of phenyl thiourea with ethyl 4-bromocrotonate (**E1**) to synthesize thiazoline. The product formation was promising and we obtained a clean product in 90% yield (entry 1, Table 1). The same reaction in 2,2,2-trifluroethanol (TFE) gave the product in 74% yield (entry 2). We tried the reaction with other common laboratory solvents. Halogenated solvents; dichloromethane and chloroform (entries 3 and 4), produced thiazoline derivative (**1**) in almost 50% yield. The reaction in polar protic solvents; methanol, ethanol, isopropanol, 1-propanol, and 2-butanol (entries 5–9) also gave the product in modest yield. The polar aprotic solvents such as acetonitrile and tetrahydrofuran (entries 10 and 11) afforded the compound (**1**) in less than 50% yield but the reaction in acetone (entry 12) gave unidentifiable products. The reaction in DMSO, DMF, or water failed to give any product. A solvent mixture of HFIP and methanol (1 : 1) is also effective to get the product in 64% yield. One equivalent CH_3_CO_2_Na and 8 hours refluxing are required for the complete product formation. Progress of the reaction was monitored by thin layer chromatography (TLC). The pure product (**1**) is isolated by filtration to get rid of the salt followed by recrystallization with methyl *tert*-butyl ether. The reaction in multigram scale also afforded the product (**1**) formation without affecting the purity and yield.

**Table 1:** Reaction of *N*-phenylthiourea with methyl 4-bromocrotonate^*a*^

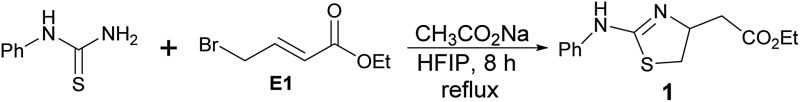		
S. no.	Solvent	Yield (%)
1	HFIP	90
2	2,2,2-Trifluroethanol	74
3	CH_2_Cl_2_	50
4	CHCl_3_	55
5	CH_3_OH	60
6	CH_3_CH_2_OH	50
7	Isopropanol	65
8	1-Propanol	55
9	2-Butanol	60
10	CH_3_CN	48
11	THF	40
12	Acetone	NR
13	DMF	NR
14	DMSO	NR
15	H_2_O	NR
16	Neat	NR
17	HFIP : MeOH (1 : 1)	64

^*a*^Reaction conditions: substrate (1.0 mmol), Michael acceptor (1.1 mmol), sodium acetate (1.1 mmol), and HFIP solvent (5 mL).

**Scheme 2:**
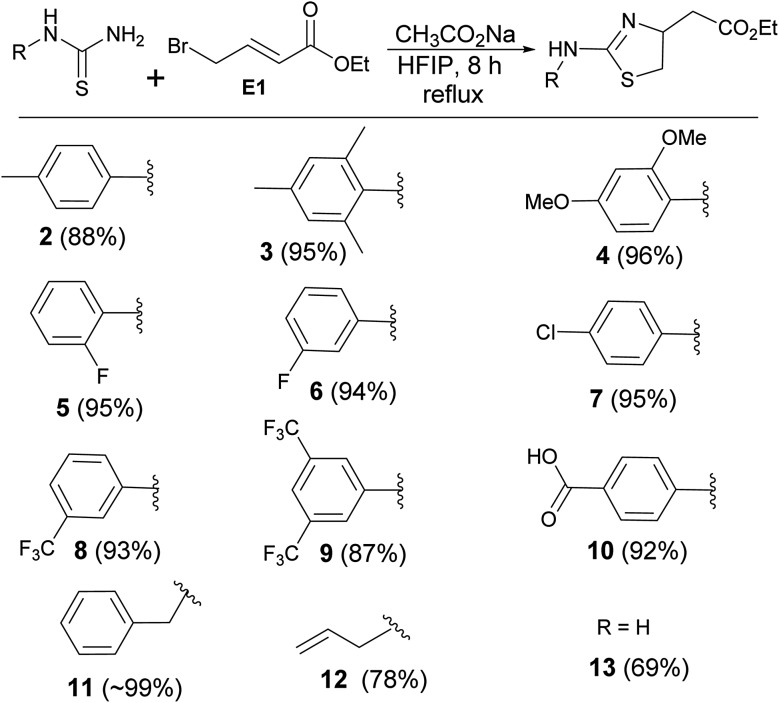
Synthesis of thiourea-derived thiazolines.

The encouraging outcome of the reaction prompted us to use other thiourea derivatives to synthesize a library of novel molecules (Scheme 2). The nature of the *N*-substituent of thiourea did not alter the outcome of the reactions. Electron donating, such as methyl and methoxy groups on the phenyl ring gave clean products (**2**, **3**, & **4**) in an average of 92% yield. Moderately electron withdrawing groups such as halogens gave the products (**5**, **6**, & **7**) without affecting the yield. Other electron withdrawing substances like trifluoromethyl and carboxylic acid gave the products (**8**, **9**, & **10**) in excellent yield (∼90%). The reaction of benzyl thiourea with the electrophile formed the product (**11**) in quantitative yield. Allyl thiourea and thiourea produced corresponding thiazolines (**12** & **13** (ref. 17)) in 78% and 69% yields respectively.

**Scheme 3:**
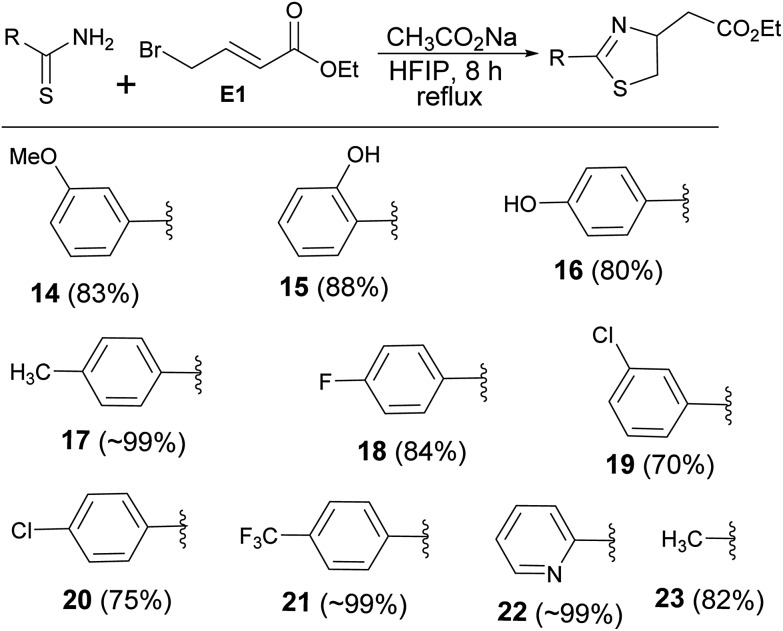
Synthesis of thiazole derivatives.

**Fig. 1:**
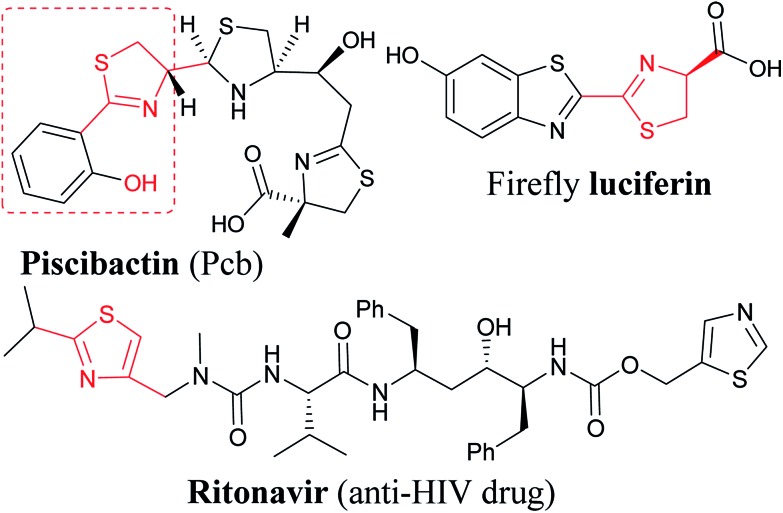
Representative example of 2,4-disubstituted thiazoline and thiazole derivatives.

Encouraged by the reaction of thiourea derivatives, we tried the reaction with thiobenzamides, and found the product formation in excellent yields (Scheme 3). Electron donating such as methoxy and hydroxy groups gave the products (**14**, **15**, & **16**) in ∼80% yield. In this series of molecules, formation of 2-hydroxyphenyl thiazoline derivative (**15**) is very significant. This moiety is found in a large number of naturally occurring siderophores such as piscibactin (Fig. 1). Siderophores are very important molecules because of their antibacterial activities.^18^ This compound^19^ could be a potential antibacterial agent and this methodology could be used to synthesize a number new thiazoline based siderophores. *p*-Tolyl derivative were formed the product (**17**) in quantitative yield and the product was obtained by filtration followed by evaporation of the solvent. Halogenated substrates like fluoro, and chloro gave the products (**18**, **19**, & **20**) in good yield. The reaction of the electrophile with trifluoromethyl thiobenzamide afforded the product (**21**) in quantitative yield. 2-Pyridinethioamide reacted with the electrophile under the standard reaction condition to give the product (**22**) in quantitative yield. Methyl substituted thiazoline (**23**) was also obtained in very good yield by reacting thioacetamide with the electrophile (**E1**). Thus, this methodology is equally applicable to heterocyclic and alkyl based thioamide substrates (Scheme 3).

**Scheme 4:**
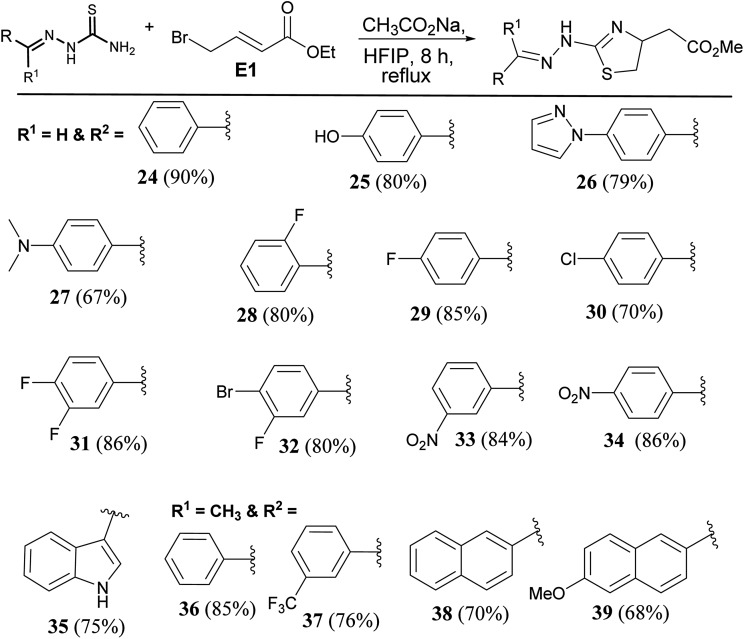
Synthesis of thiosemicarbazone derived thiazolines.

To extend the scope of the protocol, we tried the reaction with thiosemicarbazones (Scheme 4). Molecules of this class are known to show useful pharmacological properties.^20^,^21^ Benzaldehyde derived thiosemicarbazone reacted smoothly to give the product (**24**) in 90% yield. Irrespective of the nature of the substituents on the phenyl ring, products were formed in good to excellent yield. Hydroxy derivative afforded the product (**25**) in 80% yield. Pyrazole and amine substituted products (**26** & **27**) were formed in 79% and 60% yield respectively. Fluoro and chloro derivatives gave thiazolines (**28**, **29**, & **30**) in ∼78% yield. Disubstituted halogen substrates formed expected products (**31** & **32**). Nitro substituted thiazolines (**33** & **34**) were formed in an average of 83% yield. Indole derived thiosemicarbazide reacted smoothly to give the product (**35**). Ketone derived thiosemicarbazones reacted to afford expected thiazolines (**36** & **37**) in good yield. Naphthalene substituted thiazolines were formed (**38** & **39**) in an average of 69% yield (Scheme 4).

**Scheme 5:**
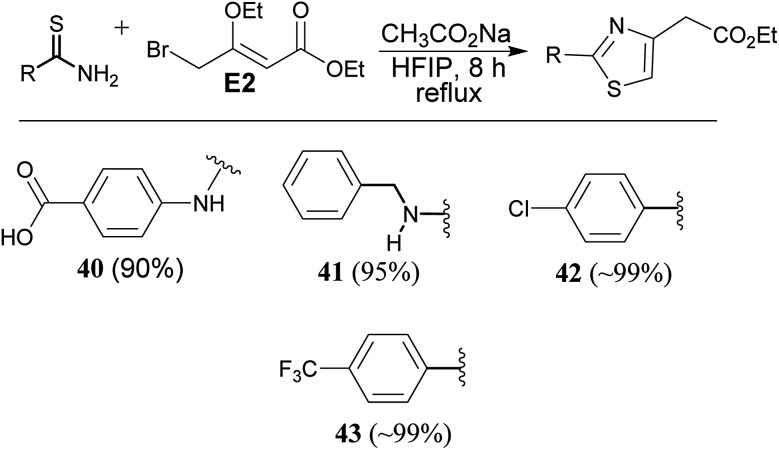
Synthesis of thiazole derivative.

Reaction of thiourea derivatives with a slightly modified electrophile, 4-bromo-3-ethoxycrotonate electrophile (**E2**) afforded thiazole derivatives (**40** & **41**) in excellent yield. Reaction of thiobenzamide derivatives gave the products (**42** & **43**) in quantitative yield. Thus, application of this methodology is equally applicable to synthesize thiazole derivatives (Scheme 5).

**Scheme 6:**
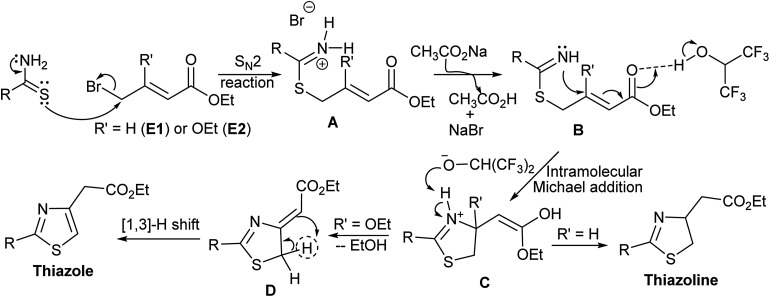
Plausible mechanism for the formation of thiazoline and thiazole derivatives.

Based on the product outcome, solvent effect, and the role of base, we propose following mechanism for this methodology (Scheme 6). The reaction starts with S_N_2 substitution of bromine of crotonate derivative (**E1** or **E2**) by the thioamide derivative. The resultant iminium bromide (**A**) reacts with the base (CH_3_CO_2_Na) to form imine derivative (**B**), which undergoes intramolecular Michael addition to form the thiazoline skeleton (**C**). Strong hydrogen bonding of HFIP with the carbonyl carbon of the Michael acceptor (**B**) facilitates the intramolecular Michael addition to form the thiazoline derived enol (**C**). The fate of this thiazole derivative (**C**) depends on the substituent (R′). For R = H, it undergoes keto–enol tautomerism to form thiazoline and for R = OEt, it undergoes elimination to form exo ylideneacetate derivative (**D**). [1,3]-Hydrogen shift^12^ of ylideneacetate (**D**) leads to the formation of thiazole derivative (Scheme 6).

## Conclusions

In this article, we designed and developed a strategy for the synthesis of novel thiazoles and thiazolines. Although, thiazole derivatives (**40–43**) can be synthesized from literature procedures but the synthesis of thiazoline derivatives (**1–39**) cannot be synthesized easily from the existing procedures. The key factor in the discovery is the use of polar and strong hydrogen bonding hexafluoroisopropanol as a solvent. Ease of synthesis and scalability to multigram scale have been the strength of the developed methodology. It gives immense scope to generate a library of new molecules required for drug discovery and biological study. Synthesis and biological studies of more thiazoline and thiazole derivatives, particularly thiazoline-androstane will be reported in due course.

## Conflict of interest

The authors declare no conflict of interest.

## Supplementary Material

Supplementary information

## References

[cit1] Chen B. and Heal W., in Comprehensive Heterocyclic Chemistry III, ed. C. A. Ramsden, E. F. V. Scriven and R. J. K. Taylor, Elsevier, Oxford, 2008, pp. 635–754.

[cit2] Ayati A., Emami S., Asadipour A., Shafiee A., Foroumadi A. (2015). Eur. J. Med. Chem..

[cit3] Rouf A., Tanyeli C. (2015). Eur. J. Med. Chem..

[cit4] Chhabria M. T., Patel S., Modi P., Brahmkshatriya P. S. (2016). Curr. Top. Med. Chem. (Sharjah, United Arab Emirates).

[cit5] Zhang S., Pattacini R. and Braunstein P., in Advances in Organometallic Chemistry and Catalysis, John Wiley & Sons, Inc., 2013, pp. 185–198.

[cit6] Bulut I., Chávez P., Mirloup A., Huaulmé Q., Hébraud A., Heinrich B., Fall S., Méry S., Ziessel R., Heiser T., Lévêque P., Leclerc N. (2016). J. Mater. Chem. C.

[cit7] Lin Y., Fan H., Li Y., Zhan X. (2012). Adv. Mater..

[cit8] Gaumont A. C., Gulea M., Levillain J. (2009). Chem. Rev..

[cit9] Alom N. E., Wu F., Li W. (2017). Org. Lett..

[cit10] Brider J., Rowe T., Gibler D. J., Gottsponer A., Delancey E., Branscum M. D., Ontko A., Gilmore D., Alam M. A. (2016). Med. Chem. Res..

[cit11] Allison D., Delancey E., Ramey H., Williams C., Alsharif Z. A., Al-khattabi H., Ontko A., Gilmore D., Alam M. A. (2017). Bioorg. Med. Chem. Lett..

[cit12] Alam M. A., Alsharif Z., Alkhattabi H., Jones D., Delancey E., Gottsponer A., Yang T. (2016). Sci. Rep..

[cit13] Bonnet-Delpon D., Bégué J.-P., Crousse B. (2004). Synlett.

[cit14] Kelley B. T., Walters J. C., Wengryniuk S. E. (2016). Org. Lett..

[cit15] Khaksar S., Talesh S. M. (2012). J. Fluorine Chem..

[cit16] Shen C., Wang L., Wen M., Shen H., Jin J., Zhang P. (2016). Ind. Eng. Chem. Res..

[cit17] Campbell M. M., Mickel S. J., Singh G. (1991). Bioorg. Med. Chem. Lett..

[cit18] Sansinenea E., Ortiz A. (2009). Mini-Rev. Org. Chem..

[cit19] Krogh A., Larsson B., Von Heijne G., Sonnhammer E. L. L. (2001). J. Mol. Biol..

[cit20] Koradin C., Kordes M., Le V. R., Culbertson D. L., Anspaugh D. D. and Cotter H. V. T., US Pat., WO2007147701A1, 2007.

[cit21] Yuan J.-W., Wang S.-F., Luo Z.-L., Qiu H.-Y., Wang P.-F., Zhang X., Yang Y.-A., Yin Y., Zhang F., Zhu H.-L. (2014). Bioorg. Med. Chem. Lett..

