# Supplementary material for: Modular synthesis of thiazoline and thiazole derivatives by using a cascade protocol
Source: RSC Adv. 2017 Jun 26;7(52):32647–51. doi: 10.1039/c7ra05993k (PMC5695723; doi:10.1039/c7ra05993k)

Electronic Supplementary Material (ESI) for RSC Advances.
This journal is © The Royal Society of Chemistry 2017

Modular synthesis of thiazoline and thiazole derivatives by using a cascade protocol

Zakeyah A Alsharif,[a] Mohammad A. Alam[a]*

Department of Chemistry and Physics, College of Science and Mathematics, Arkansas State University, Jonesboro, AR 72467
*Corresponding author
Mohammad A Alam
E-mail: malam@astate.edu, Tel: +1-870-973-3319

Materials and instrumentation

All commercial chemicals and solvents are reagent grade and were used without further treatment unless otherwise noted. ^{1}H NMR spectra were obtained with a Varian Mercury-300MHz with TMS as internal standard. ^{13}C NMR spectra were obtained with a Varian Mercury-75MHz with TMS as internal standard. The ESI-FTMS Mass spectra were recorded Bruker ApexII-FTMS system. Low resolution mass spectra were recorded in Shimadzu QQQ instrument.

General Procedure

A mixture of thioamide derivative (1.0 mmol), Michael acceptor (1.1 mmol), and sodium acetate (1.1 mmol) in HFIP (5 mL) was for eight hours. Filtration to remove sodium bromide followed by evaporation gave the crude product. Recrystallization with ether or ethyl acetate followed by filtration afforded the pure product.

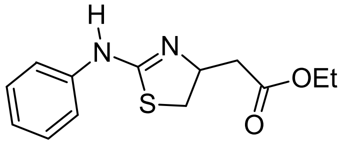

Ethyl (2-anilino-4,5-dihydro-1,3-thiazol-4-yl)acetate (1)

Beige powder, yield = 90%, m. p. = 158.8 °C, ^{1}H NMR (DMSO-d_6, 300MHz): δ (ppm) 9.39 (s, 1H), 7.51 (s, 5H), 4.90 (s br, 1H), 3.95 (t, J = 9.0 Hz, 1H), 3.86 (q, J = 6.99 Hz, 1H), 3.50 – 3.45 (m, 1H), 2.81 (dd, J = 5.9, 16.2 Hz, 1H), 2.58-2.49 (m, 2H), 1.04 (t, J = 5.0 Hz, 3H); ^{13}C NMR (75MHz, DMSO-d_6) δ = 170.1, 169.7, 134.9, 130.7, 130.3, 128.3, 65.0, 60.9, 36.3, 33.5, 14.3. HRMS (ESI-FTMS Mass (m/z): calcd for $C_{13}H_{17}N_2O_2S$ $[M + H]^+$ 265.1005, found 265.1009.

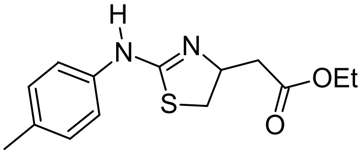

Ethyl [2-(4-methylanilino)-4,5-dihydro-1,3-thiazol-4-yl]acetate (2)

white powder, yield = 88%, m. p. = 140.0 °C, ^{1}H NMR (DMSO-d_6, 300MHz): δ (ppm) 7.41 – 7.34 (m, 4H), 4.90 – 4.86 (m, 1H), 3.91 (q, J=7.74 Hz, 3H), 3.49 (dd, J = 6.6, 9.9 Hz, 1H), 2.79 (dd, , J = 9.3, 16.8 Hz, 1H), 2.60 – 2.59 (m, 1H), 2.61 (s, 3H), 2.49 (t, J = 1.5 Hz, 3H); ^{13}C NMR (75MHz, DMSO-d_6) δ = 170.9, 169.6, 140.5, 131.9, 131.3, 128.1, 65.4, 60.9, 36.2, 33.3, 21.2, 14.2. HRMS (ESI-FTMS Mass (m/z): calcd for $C_{14}H_{19}N_2O_2S$ $[M + H]^+$ 279.1162, found 279.1165.

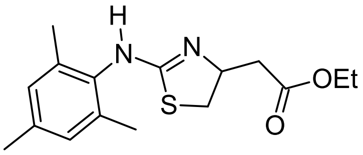

Ethyl [2-(2,4,6-trimethylanilino)-4,5-dihydro-1,3-thiazol-4-yl]acetate (3)

White powder, yield = 95%, m. p. = 154.8 °C, ^{1}H NMR (DMSO-d_6, 300MHz): δ (ppm) 8.81 (s br, 1H), 7.06 (d, J = 5.2 Hz, 2H), 4.86 – 4.81 (m, 1H), 3.93 (dd, J = 7.2, 11.1Hz, 1H), 3.79 – 3.73 (m, 2H), 3.66 (t, J = 10.8 Hz, 1H), 2.92 (dd, J = 6.4, 16.4 Hz, 1H), 2.42 (d, J = 4.7 Hz, 1H), 2.50 – 2.41 (m, 3H), 2.13 (d, J = 4.1 Hz, 6H), 1.01 (t, J = 6.9 Hz, 3H); ^{13}C NMR (75MHz, DMSO-d_6) δ = 170.6, 169.3, 140.4, 137.0, 136.6, 130.5, 130.4, 128.8, 63.9, 60.9, 36.2, 33.9, 21.0, 18.0, 17.8, 14.1. HRMS (ESI-FTMS Mass (m/z): calcd for $C_{16}H_{23}N_2O_2S$ $[M + H]^+$ 307.1475, found 307.1478.

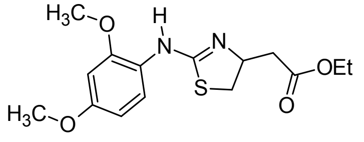

Ethyl [2-(2,4-dimethoxyanilino)-4,5-dihydro-1,3-thiazol-4-yl]acetate (4)

White powder, yield = 96%, m. p. = 190.0 °C, ^{1}H NMR (DMSO-d_6, 300MHz): δ (ppm) 7.30 (br s, 1H), 6.75 (d, J = 2.4 Hz, 1H), 6.68 (dd, J = 2.4, 8.7 Hz, 1H), 4.73 (br s, 1H), 4.05 – 3.82 (m, 2H), 3.81 (s, 6H), 3.52 – 3.45 (m, 3H), 2.80 (dd, J = 6.5, 16.5 Hz, 1H), 1.09 (t, J = 7.0 Hz, 3H); ^{13}C NMR (75MHz, DMSO-d_6) δ = 169.6, 162.5, 156.7, 130.2, 114.7, 106.5, 106.2, 100.3, 99.5, 41.9, 60.9, 56.6, 56.2, 36.5, 33.3, 14.2. HRMS (ESI-FTMS Mass (m/z): calcd for $C_{15}H_{21}N_2O_4S$ $[M + H]^+$ 325.1217, found 325.1220.

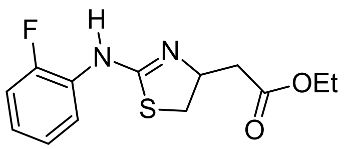

Ethyl [2-(2-fluoroanilino)-4,5-dihydro-1,3-thiazol-4-yl]acetate (5)

White powder, yield = 95%, m. p. = 183.1 °C, ^{1}H NMR (DMSO-d_6, 300MHz): δ (ppm) 9.18 (s, 1H), 7.66 – 7.59 (m 2H), 7.53 – 7.46 (m, 1H), 7.43 – 7.38 (m, 1H), 4.89 (br s, 1H), 3.96 (t, J = 10.4 Hz, 1H), 3.85 – 3.83 (m, 2H), 3.60 – 3.53 (m, 1H), 2.88 (d, J = 11.4 Hz, 1H), 2.5 (br s, 1H), 1.05 (t, J = 7.0 Hz, 3H); ^{13}C NMR (75MHz, DMSO-d_6) δ = 172.0, 169.4, 158.1 ($^{1}J_{C-F}$ =249.4 Hz), 133.4 ($^{3}J_{C-F}$ = 7.4 Hz), 130.7, 126.6, 121.6 ($^{3}J_{C-F}$ = 12.90 Hz), 117.9 ($^{2}J_{C-F}$ = 18.9 Hz), 64.9, 60.9, 36.3, 33.8, 14.2. HRMS (ESI-FTMS Mass (m/z): calcd for $C_{13}H_{16}FN_2O_2S$ $[M + H]^+$ 283.0911, found 283.0914.

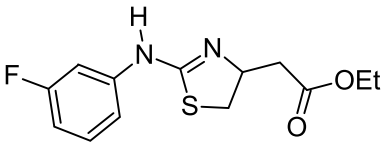

Ethyl [2-(3-fluoroanilino)-4,5-dihydro-1,3-thiazol-4-yl]acetate (6)

Brownish powder, yield = 95%, m. p. = 135.0 °C, ^{1}H NMR (DMSO-d_6, 300MHz): δ (ppm) 7.59 – 7.55 (m, 3H), 7.48 – 7.43 (m, 1H), 4.10 (q, J = 7.1 Hz, 3H), 3.73 (dd, J = 8.7, 11.2 Hz, 1H), 3.30 (dd, J = 8.0, 11.2 Hz, 1H), 2.79 (dd, J = 1.7, 6.6 Hz, 2H), 1.18 (t, J = 6.9 Hz, 3H); ^{13}C NMR (75MHz, DMSO-d_6) δ = 170.8, 168.8, 162.3 ($^{1}J_{C-F}$ = 243.6 Hz) , 133.9, 131.7 ($^{3}J_{C-F}$ = 8.1 Hz), 125.1, 119.7 ($^{2}J_{C-F}$ = 20.7 Hz), 114.8 ($^{2}J_{C-F}$ = 23.0 Hz), 72.3, 60.6, 38.7, 37.9, 14.5. HRMS (ESI-FTMS Mass (m/z): calcd for $C_{13}H_{16}FN_2O_2S$ $[M + H]^+$ 283.0911, found 283.0913.

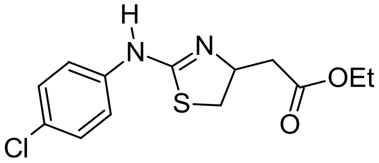

Ethyl [2-(4-chloroanilino)-4,5-dihydro-1,3-thiazol-4-yl]acetate (7)

White powder, yield = 95%, m. p. = 202.3 °C, ^{1}H NMR (DMSO-d_6, 300MHz): δ (ppm) 7.68 (d, J= 7.41 Hz, 2H), 7.54 (d, J= 7.5 Hz, 2H), 4.96 – 4.87 (m, 1H), 3.96 – 3.89 (m, 3H), 3.52 – 3.35 (m, 1H), 2.84 (dd, J = 9.3, 16.3, 1H), 2.53 (dd, J = 5.2, 16.6 Hz, 1H), 1.10 (t, J= 1.53 Hz, 3H); ^{13}C NMR (75MHz, DMSO-d_6) δ = 171.2, 169.7, 135.4, 133.4, 130.9, 130.6, 65.2, 60.9, 36.3, 33,6, 14.2. HRMS (ESI-FTMS Mass (m/z): calcd for $C_{13}H_{16}ClN_2O_2S$ $[M + H]^+$ 301.0586, found 301.0588.

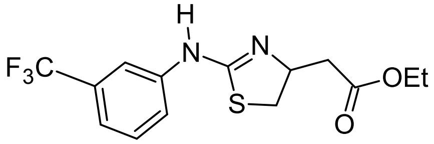

Ethyl {2-[3-(trifluoromethyl)anilino]-4,5-dihydro-1,3-thiazol-4-yl}acetate (8)

White powder, yield = 93%, m. p. = 210.1 °C, ^{1}H NMR (DMSO-d_6, 300MHz): δ (ppm) 8.92 (br s, 1H), 8.02 (s, 1H), 7.93 (s, 1H), 7.83 – 7.79 (m, 2H), 5.00 (quint, J = 6.9 Hz, 1H), 3.96 (dd, J = 7.8, 11.3 Hz, 1H), 3.86 (q, J = 6.7 Hz, 2H), 3.50 (dd, J = 6.7, 11.3 Hz, 1H), 2.86 (dd, J = 6.7, 16.7 Hz, 1H), 2.66 (dd, J = 5.6, 16.8 Hz, 1H), 1.05 (t, J = 7.0 Hz, 3H); ^{13}C NMR (75MHz, DMSO-d_6) δ = 171.6, 169.6, 135.3, 133.2, 132.1, 131.3 (q, $^{2}J_{C-F}$ = 32.1 Hz), 127.7, 126.1, 123.9 ($^{1}J_{C-F}$ = 270 Hz), 65.4, 60.9, 36.3, 33.8, 14.2. HRMS (ESI-FTMS Mass (m/z): calcd for $C_{14}H_{16}F_3N_2O_2S$ $[M + H]^+$ 333.0879, found 333.0882.

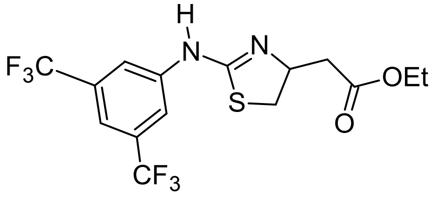

Ethyl {2-[3,5-bis(trifluoromethyl)anilino]-4,5-dihydro-1,3-thiazol-4-yl}acetate (9)

White powder, yield = 87%, m. p. = 148.1 °C, ^{1}H NMR (DMSO-d$_6$, 300MHz): δ (ppm) 9.08 (s, 1H), 8.40 (s, 2H), 8.36 (s, 1H), 5.04 (quint, J = 6.5 Hz,1H), 3.96 (dd, J = 7.7, 11.1 Hz , 1H), 3.82 (q, J = 7.0 Hz, 2H), 3.49 (dd, J = 6.7, 11.2 Hz, 1H), 2.94 (dd, J = 6.4, 16.9 Hz, 1H), 2.76 (dd, J = 6.0, 16.8 Hz, 1H), 1.03 (t, J = 1.2 Hz, 3H); ^{13}C NMR (75MHz, DMSO-d$_6$) δ = 172.1, 169.8, 136.5, 132.6 ($^2J_{C-F}$ = 33.6), 131.0, 125.0, 121.4, 65.3, 60.9, 36.3, 34.1, 14.1. HRMS (ESI-FTMS Mass (m/z): calcd for $C_{15}H_{15}F_6N_2O_2S$ [M + H]$^+$ 401.0753, found 401.0754.

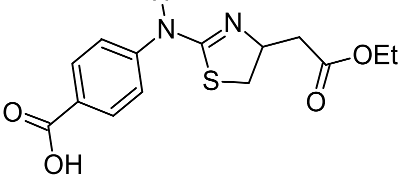

4-{[4-(2-Ethoxy-2-oxoethyl)-4,5-dihydro-1,3-thiazol-2-yl]amino}benzoic acid (10)

White powder, yield = 92%, m. p. = 250.0 °C, ^{1}H NMR (DMSO-d$_6$, 300MHz): δ (ppm) 8.09 (d, J = 8.4 Hz, 2H), 7.64 (d, J = 8.4 Hz, 2H), 5.00 – 4.96 (m, 1H), 3.97 – 3.86 (m, 3H), 3.50 (dd, J = 6.3, 11.3 Hz, 1H), 2.85 (dd, J = 7.4, 16.6 Hz, 1H), 2.64 (dd, J = 4.9, 16.5 Hz, 1H), 1.07 (t, J = 7.0Hz, 3H); ^{13}C NMR (75MHz, DMSO-d$_6$) δ = 170.4, 169.7, 166.9, 138.7, 132.3, 131.6, 128.5, 64.9, 60.9, 36.2, 33.5, 14.2. HRMS (ESI-FTMS Mass (m/z): calcd for $C_{14}H_{17}N_2O_4S$ [M + H]$^+$ 309.0904, found 309.0907.

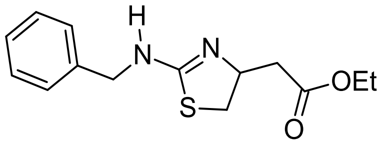

Ethyl [2-(benzylamino)-4,5-dihydro-1,3-thiazol-4-yl]acetate (11)

White powder, yield = 97%, m. p. = 139.3 °C, ^{1}H NMR (DMSO-d$_6$, 300MHz): δ (ppm) 9.78 (s, 1H), 7.44 – 7.36 (m, 3H), 7.30 (d, J = 7.5 Hz, 2H), 4.94 (d, J = 16.5 Hz, 1H), 4.69 (d, J = 16.4 Hz, 1H), 4.44 (s br, 1H), 4.04 (q, J = 7.2 Hz, 2H), 3.84 (dd, J = 8.07, 11.19 Hz, 1H), 3.35 (dd, J = 3, 11.4 Hz, 1H) 2.85 – 2.83 (m, 2H), 1.18 (t, J = 7.0 Hz, 3H); ^{13}C NMR (75MHz, DMSO-d$_6$) δ = 170.6, 170.1, 134.3, 129.4, 128.5, 127.7, 62.3, 61.0, 48.4, 35.0, 33.3, 14.3. HRMS (ESI-FTMS Mass (m/z): calcd for $C_{14}H_{19}N_2O_2S$ [M + H]$^+$ 279.1162, found 279.1165.

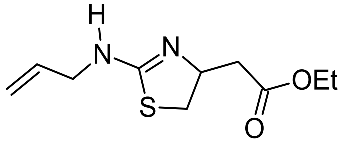

Ethyl {2-[(prop-2-en-1-yl)amino]-4,5-dihydro-1,3-thiazol-4-yl}acetate (12)

White powder, m. p. = 90.4 ^{o}C yield = 78%, ^{1}H NMR (DMSO-d$_6$, 300MHz): 97.9, δ (ppm) 9.56 (s, 1H), 5.86 – 5.76 (m, 1H), 5.29 (t, J = 16.9 Hz, 2H), 4.54 – 4.51 (m, 1H), 4.30 (d, J = 16.8 Hz, 1H), 4.12 – 4.05 (m, 3H), 3.82 (dd, J = 7.9, 11.4 Hz, 1H), 3.37-3.34 (m, 1H), 2.91 – 2.77 (m, 2H), 1.19 (t, J = 7.1 Hz, 3H); ^{13}C NMR (75MHz, DMSO-d$_6$) δ = 170.1, 169.8, 130.7, 119.0, 62.3, 61.0, 47.6, 35.2, 33.3, 14.4. HRMS (ESI-FTMS Mass (m/z): calcd for $C_{10}H_{17}N_2O_2S$ [M + H]$^+$ 229.1005, found 229.1007.

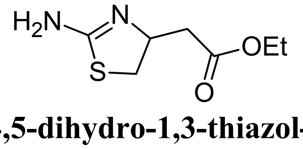

Ethyl (2-amino-4,5-dihydro-1,3-thiazol-4-yl)acetate (13)

Brownish powder, m. p. = 80.0 °C, yield = 69%, ^{1}H NMR (DMSO-d$_6$, 300MHz): δ (ppm) 4.49 (quint, J = 7.2 Hz, 1H), 4.09 (q, J = 7.1 Hz, 2H), 3.70 (dd, J = 7.7, 11.3 Hz, 1H), 3.30 (dd, J = 6.3, 11.3 Hz, 1H), 2.75 (d, J = 5.6 Hz, 2H), 1.19 (t, J = 7.0 Hz, 3H); ^{13}C NMR (75MHz, DMSO-d$_6$) δ = 171.6, 170.5, 60.9, 58.6, 37.9, 35.9, 14.4. HRMS (ESI-FTMS Mass (m/z): calcd for $C_7H_{13}N_2O_2S$ [M + H]$^+$ 189.0692, found 189.0695.

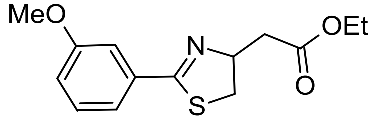

Ethyl [2-(3-methoxyphenyl)-4,5-dihydro-1,3-thiazol-4-yl]acetate (14)

Brownish powder, m. p. = 118.9 °C, yield = 83%, ^{1}H NMR (DMSO-d$_6$, 300MHz): δ (ppm) 7.45 - 7.39 (m, 1H), 7.34 – 7.29 (m, 2H), 7.16 – 7.15 (m, 1H), 4.97 – 4.92 (m, 1H), 4.12 (q, J = 7.0 Hz, 2H), 3.79 (s, 3H), 3.68 (dd, J = 10.2, 19.2 Hz, 1H), 3.26 (dd, J = 7.9, 19.1 Hz, 1H), 2.80 – 2.73 (m, 2H), 1.20 (t, J = 7.14 Hz, 3H) ; ^{13}C NMR (75MHz, DMSO-d$_6$) δ = 170.9, 169.1 159.7, 133.4, 130.6, 121.2, 118.4, 113.1, 72.7, 60.6, 55.8, 38.8, 37.6, 14.5. HRMS (ESI-FTMS Mass (m/z): calcd for $C_{14}H_{18}NO_3S$ [M + H]$^+$ 280.1002, found 280.1005.

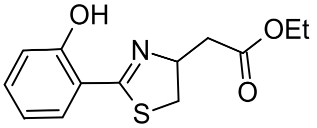

Ethyl [2-(2-hydroxyphenyl)-4,5-dihydro-1,3-thiazol-4-yl]acetate (15)

Brownish powder, m. p. = 162.5 °C, yield = 88%, ^{1}H NMR (DMSO-d$_6$, 300MHz): δ (ppm) 7.58 (d, J = 7.9 Hz, 1H), 7.51 (t, J = 8.1 Hz, 1H), 7.05 – 6.96 (m, 2H), 5.02 (quint, J =7.8 Hz, 1H), 4.10 (q, J = 7.0 Hz, 2H), 3.76 (t, J = 11.1 Hz, 1H), 3.33 (dd, J = 7.6, 11.4 Hz, 1H), 2.87 (d, J = 6.7 Hz, 1H), 2.84-2.79 (m, 1H), 1.17 (t, J = 7.1 Hz, 3H); ^{13}C NMR (75MHz, DMSO-d$_6$) δ = 176.4, 170.6, 159.0, 135.9, 131.1, 120.0, 117.5, 114.5, 68.3, 60.8, 38.3, 35.7, 14.4. HRMS (ESI-FTMS Mass (m/z): calcd for $C_{13}H_{16}NO_3S$ [M + H]$^+$ 266.0845, found 266.0849.

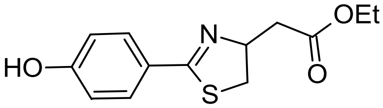

Ethyl [2-(4-hydroxyphenyl)-4,5-dihydro-1,3-thiazol-4-yl]acetate (16)

White powder, m. p. = 115.6 °C, yield = 80%, ^{1}H NMR (DMSO-d$_6$, 300MHz): δ (ppm) 7.84 (d, J = 8.7 Hz, 2H), 6.97 (d, J = 1.9 Hz, 2H), 5.04 – 4.99 (m, 2H), 4.10 (q, J = 7.1 Hz, 2H), 3.87 (dd, J = 7.7, 11.3 Hz, 1H), 3.48 (dd, J = 7.7, 11.3 Hz, 1H), 3.06 – 2.72 (m, 1H), 1.18 (t, J = 2.7 Hz, 3H); ^{13}C NMR (75MHz, DMSO-d$_6$) δ = 179.4, 170.2, 164.4, 132.4, 118.6, 116.7, 66.2, 60.9, 37.6, 36.6, 14.4. HRMS (ESI-FTMS Mass (m/z): calcd for $C_{13}H_{16}NO_3S$ [M + H]$^+$ 266.0845, found 266.0849.

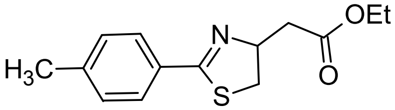

Ethyl [2-(4-methylphenyl)-4,5-dihydro-1,3-thiazol-4-yl]acetate (17)

Viscous liquid, yield = ~99%, ^{1}H NMR (DMSO-d$_6$, 300MHz): δ (ppm) 7.63 (d, J = 7.4 Hz, 2H), 7.27 (d, J = 7.8 Hz, 2H), 4.89 (q, J = 7.6 Hz, 1H), 4.10 (q, J = 7.0 Hz, 2H), 3.60 (dd, J = 8.5, 10.5 Hz, 1H), 3.16 (dd, J = 8.0, 10.9 Hz, 1H), 2.79-2.64 (m, 2H), 2.33 (s, 3H), 1.19 (t, J = 7.1 Hz, 3H); ^{13}C NMR (75MHz, DMSO-d$_6$) δ = 171.1, 166.3, 141.9, 130.3, 129.7, 128.4, 73.9, 60.5, 39.0, 37.7, 21.4, 14.5. HRMS (ESI-FTMS Mass (m/z): calcd for $C_{13}H_{16}NO_3S$ [M + H]$^+$ 264.3626, found 264.3624.

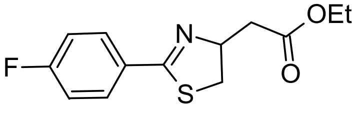

Ethyl [2-(4-fluorophenyl)-4,5-dihydro-1,3-thiazol-4-yl]acetate (18)

White powder, m. p. = 150.2 °C, yield = 84%, ^{1}H NMR (DMSO-d$_6$, 300MHz): δ (ppm) 7.84 (dd, J = 5.6, 7.9 Hz, 2H), 7.35 (t, J = 8.7 Hz, 2H), 4.99 – 4.89 (m, 1H), 4.10 (q, J = 7.0 Hz, 2H), 3.71 (dd, J = 8.7, 11.1 Hz, 1H), 3.28 (dd, J = 8.0, 11.1 Hz, 1H), 2.84-2.76 (m, 2H), 1.11 (t, J = 7.1 Hz, 3H); ^{13}C NMR (75MHz, DMSO-d$_6$) δ =

170.9, 165.0 ($^{1}J_{C-F}$ = 248.6 Hz), 131.2 ($^{3}J_{C-F}$ = 8.4 Hz), 128.6, 116.4 ($^{2}J_{C-F}$ = 21.9 Hz), 72.6, 60.6, 39.0, 38.8, 37.9, 14.5. HRMS (ESI-FTMS Mass (m/z): calcd for $C_{13}H_{15}FNO_2S$ $[M + H]^+$ 268.0802, found 268.0805.

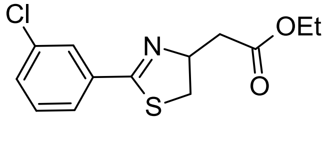

Ethyl [2-(3-chlorophenyl)-4,5-dihydro-1,3-thiazol-4-yl]acetate (19)

Brownish powder, m. p. = 144.9 °C, yield = 70%, ^{1}H NMR (DMSO-d_6, 300MHz): δ (ppm) ; 7.64 (s, 1H), 7.64 – 7.65 (m, 2H), 7.54 (d, J =7.0 Hz, 1H), 5.01 – 4.91 (m, 1H), 4.09 (q, J = 7.05 Hz, 2H), 3.30 (dd, J = 8.1, 11.1 Hz, 1H), 2.79 (dd, J = 7.2, 16.2 Hz, 1H), 2.76 – 2.71 (m, 2H), 1.19 (t, J = 6.0 Hz, 3H); ^{13}C NMR (75MHz, DMSO-d_6) δ = 170.8, 168.2, 134.0, 133.8, 132.4, 131.4, 127.7, 127.6, 72.6, 60.6, 38.7, 37.9, 14.5. HRMS (ESI-FTMS Mass (m/z): calcd for $C_{13}H_{15}ClNO_2S$ $[M + H]^+$ 286.0477, found 286.0481.

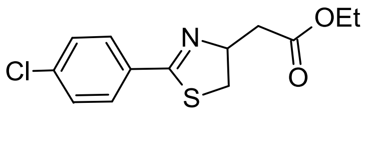

Ethyl [2-(4-chlorophenyl)-4,5-dihydro-1,3-thiazol-4-yl]acetate (20)

White powder, m. p. = 131.9 °C, yield = 75%, ^{1}H NMR (DMSO-d_6, 300MHz): δ (ppm) 7.95 (d, J = 1.5 Hz, 2H), 7.60 (d, J = 1.6 Hz, 2H), 5.00 – 4.90 (m, 1H), 4.10 (q, J = 7.0 Hz, 2H), 3.75 – 3.68 (m, 1H), 3.29 (dd, J = 8.1, 11.1 Hz, 1H), 2.77 (d, J = 6.9 Hz, 2H), 1.19 (t, J = 7.0 Hz, 3H); ^{13}C NMR (75MHz, DMSO-d_6) δ = 170.9, 168.2, 137.3, 130.8, 130.3, 129.5, 72.7, 60.6, 38.8, 37.9, 14.5. HRMS (ESI-FTMS Mass (m/z): calcd for $C_{13}H_{15}ClNO_2S$ $[M + H]^+$ 286.0477, found 286.0481.

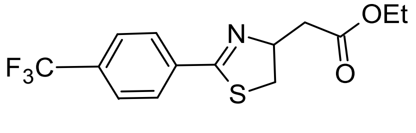

Ethyl {2-[4-(trifluoromethyl)phenyl]-4,5-dihydro-1,3-thiazol-4-yl}acetate (21)

White powder, m. p. = 148.0 °C, yield = 98%, ^{1}H NMR (DMSO-d_6, 300MHz): δ (ppm) 7.95 (d, J = 8.4 Hz, 2H), 7.86 (d, J = 8.4 Hz, 2H), 5.00 – 4.92 (m, 1H), 4.09 (q, J = 7.1 Hz, 2H), 3.28 (dd, J = 2.5, 11.19 Hz, 1H), 3.28 (dd, J = 8.2, 11.16 Hz, 1H), 2.78 (dd, J = 1.8, 10.86 Hz, 2H), 1.14 (t, J = 7.0 Hz, 3H); ^{13}C NMR (75MHz, DMSO-d_6) δ = 170.9, 166.3, 136.2, 132.0 (q, $^{1-3}J_{C-F}$ = 31.8 Hz), 129.2, 126.3 (q, $^{1-4}J_{C-F}$ = 3.7 Hz), 73.8, 60.5, 39.0, 38.1, 14.5. HRMS (ESI-FTMS Mass (m/z): calcd for $C_{14}H_{15}F_3NO_2S$ $[M + H]^+$ 318.077, found 318.0772.

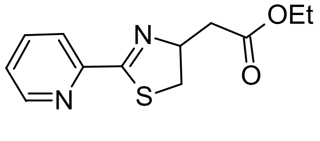

Ethyl [2-(pyridin-2-yl)-4,5-dihydro-1,3-thiazol-4-yl]acetate (22)

Viscous liquid, yield ~ 99%, ^{1}H NMR (DMSO-d_6 + $CDCl_3$, 300MHz): δ (ppm) 8.63 (d, J = 4.5 Hz, 1H), 8.00-7.95 (m, 1H), 7.89 (t, J = 7.5 Hz, 1H), 7.51 (t, J = 6.7 Hz, 1H), 5.00 (quint, J = 8.0 Hz, 2H), 4.10 (q, J = 7.1 Hz, 2H), 3.54 (dd, J = 11.2 Hz, 1H), 3.09 (dd, J = 8.1, 11.3 Hz, 1H), 2.83-2.66 (m, 2H), 1.20 (t, J = 7.1 Hz, 3H); ^{13}C NMR (75MHz, DMSO-d_6 + $CDCl_3$) δ = 170.8, 170.1, 150.4, 149.6, 137.4, 126.4, 121.7, 74.2, 60.5, 39.1, 36.5, 14.5. HRMS (ESI-FTMS Mass (m/z): calcd for $C_{12}H_{15}N_2O_2S$ $[M + H]^+$ 251.0854, found 251.0854.

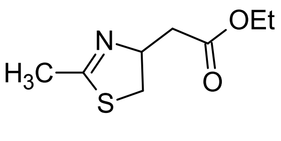

Ethyl (2-methyl-4,5-dihydro-1,3-thiazol-4-yl)acetate (23)

Viscous liquid, yield = 82%, ^{1}H NMR (DMSO-d$_6$ + CDCl$_3$, 300MHz): δ (ppm) 4.13-3.99 (m, 3H), 2.62-2.55 (m, 2H), 2.50-2.40 (m, 2H), 1.80 (s, 3H), 1.17 (t, J = 7.0 Hz, 3H); ^{13}C NMR (75MHz, DMSO-d$_6$ + CDCl$_3$) δ = 170.8, 169.3, 60.3, 48.6, 37.6, 28.2, 23.0, 14.4. HRMS (ESI-FTMS Mass (m/z): calcd for $C_8H_{14}NO_2S$ [M + H]$^+$ 188.0745, found 188.0848.

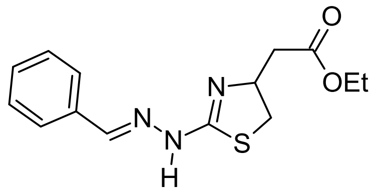

Ethyl {2-[(2*E*)-2-benzylidenehydrazinyl]-4,5-dihydro-1,3-thiazol-4-yl}acetate (24)

White powder, m. p. = 234.8 °C, yield = 90%, ^{1}H NMR (DMSO-d$_6$ + CDCl$_3$, 300MHz): δ (ppm) 10.08 (br s, 1H), 8.39 (s, 1H), 8.01 (d, J= 7.74 Hz, 2H), 7.51 (s, 3H), 5.45 – 5.41 (m, 1H), 4.11 (q, J= Hz, 2H), 3.94 – 3.88 (m, 1H), 3.53 (d, J= 11.67 Hz, 1H), 2.85 – 2.83 (m, 2H), 1.20 (t, J = 7.0 Hz, 3H) ; ^{13}C NMR (75MHz, DMSO-d$_6$ + CDCl$_3$) δ = 169.5, 168.6, 151.5, 133.1, 131.8, 129.1, 128.9, 61.1, 56.8, 32.5, 32.3, 14.3. HRMS (ESI-FTMS Mass (m/z): calcd for $C_{14}H_{18}N_3O_2S$ [M + H]$^+$ 292.1114, found 292.1118.

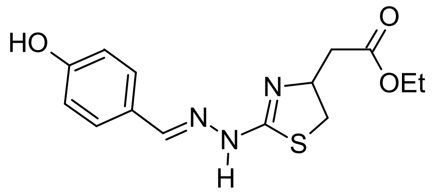

Ethyl (2-{(2*E*)-2-[(4-hydroxyphenyl)methylidene]hydrazinyl}-4,5-dihydro-1,3-thiazol-4-yl)acetate (25)

Brownish powder, m. p. = 241.4 °C, yield = 80%, ^{1}H NMR (DMSO-d$_6$, 300MHz): δ (ppm) 10.17 (br s, 1H), 9.94 (br s, 1H), 8.27 (s, 1H), 7.84 (d, J = 7.6 Hz, 2H), 6.87 (d, J = 7.5 Hz, 2H), 5.37 (br s, 1H), 4.08 (q, J = 7.1 Hz, 2H), 3.88 (dd, J = 7.8, 11.3 Hz, 1H), 3.50 (d, J = 11.5 Hz, 1H), 2.86 – 2.73 (m, 2H), 1.19 (t, J = 7.1 Hz, 3H); ^{13}C NMR (75MHz, DMSO-d$_6$) δ = 170.0, 167.6, 161.2, 151.4, 131.1, 124.3, 116.1, 61.1, 56.8, 32.4, 32.2, 14.4. HRMS (ESI-FTMS Mass (m/z): calcd for $C_{14}H_{18}N_3O_3S$ [M + H]$^+$ 308.1063, found 308.1066.

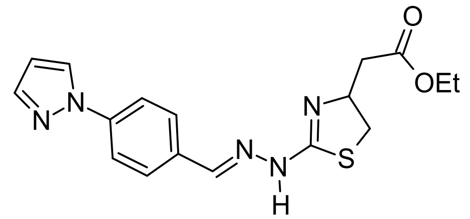

Ethyl {2-[(2*E*)-2-{[4-(1*H*-pyrazol-1-yl)phenyl]methylidene}hydrazinyl]-4,5-dihydro-1,3-thiazol-4-yl}acetate (26)

Brownish powder, m. p. = 193.7 °C, yield = 79%, ^{1}H NMR (DMSO-d$_6$, 300MHz): δ (ppm) 10.0 (br s, 1H), 8.65 (d, J = 2.4 Hz, 1H), 8.41 (s, 1H), 8.14 (d, J = 8.5 Hz, 2H), 8.05 (d, J = 8.5 Hz, 2H), 7.81 (s, 1H), 6.60 (s, 1H), 5.43 – 5.40 (m, 1H), 4.11 (q, J = 7.0 Hz, 2H), 3.95 – 3.88 (m, 1H), 3.55 – 3.51 (m, 1H), 2.85 (d, J = 6.0 Hz, 2H), 1.20 (t, J = 7.1 Hz, 3H); ^{13}C NMR (75MHz, DMSO-d$_6$) δ = 170.0, 168.6, 150.1, 142.2, 142.0, 131.0, 130.4, 128.6, 118.6, 109.0, 61.1, 56.8, 32.4, 32.3, 14.4. HRMS (ESI-FTMS Mass (m/z): calcd for $C_{17}H_{20}N_5O_2S$ [M + H]$^+$ 358.1332, found 358.1335.

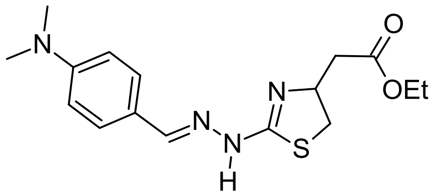

Ethyl {2-[(2*E*)-2-{[4-(dimethylamino)phenyl]methylidene}hydrazinyl]-4,5-dihydro-1,3-thiazol-4-yl}acetate (26)

Brownish powder, m. p. = 180.6 °C, yield = 67%, ^{1}H NMR (DMSO-d_6, 300MHz): δ (ppm) 8.21 (s, 1H), 7.78 (d, J = 7.3 Hz, 2H), 6.75 (d, J = 7.4 Hz, 2H), 5.36 (br s, 1H), 4.09 (q, J = 5.5 Hz, 2H), 3.85 (t, J = 10.8 Hz, 2H), 3.00 (s, 6H), 2.85 – 2.76 (m, 2H), 1.21 (t, J =Hz, 5.2, 3H); ^{13}C NMR (75MHz, DMSO-d_6) δ = 170.0, 166.7, 152.8, 152.0, 130.6, 120.1, 111.8, 61.1, 56.9, 32.7, 32.0, 14.3. HRMS (ESI-FTMS Mass (m/z): calcd for $C_{16}H_{23}N_4O_2S$ $[M + H]^+$ 335.1536, found 335.1541.

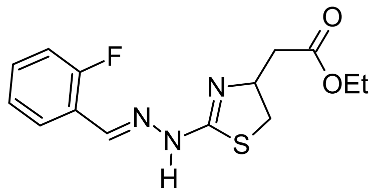

Ethyl (2-{(2*E*)-2-[(2-fluorophenyl)methylidene]hydrazinyl}-4,5-dihydro-1,3-thiazol-4-yl)acetate (28)

Brownish powder, m. p. = 228.9 °C, yield = 80%, ^{1}H NMR (DMSO-d_6, 300MHz): δ (ppm) 8.32 (s, 2H), 7.61 – 7.54 (m, 1H), 7.36 – 7.29 (m, 1H), 5.57 – 5.55 (m, 1H), 4.05 (q, J = 7.0 Hz, 3H), 3.91 – 3.85 (m, 2H), 2.83 (d, J = 5.6 Hz, 2H), 1.18 (t, J = 6.0 Hz, 3H); ^{13}C NMR (75MHz, DMSO-d_6) δ = 170.0, 169.4, 162.4 ($^{1}J_{C\text{-}F}$ = 251.5 Hz), 144.0, 134.4, 128.2, 125.3, 120.7 ($^{3}J_{C\text{-}F}$ = 9.5 Hz), 116.4 ($^{2}J_{C\text{-}F}$ = 20.4 Hz), 61.3, 56.7, 38.8, 32.8, 14.3. HRMS (ESI-FTMS Mass (m/z): calcd for $C_{14}H_{17}FN_3O_2S$ $[M + H]^+$ 310.102, found 310.1024.

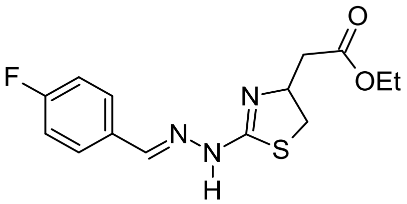

Ethyl (2-{(2*E*)-2-[(4-fluorophenyl)methylidene]hydrazinyl}-4,5-dihydro-1,3-thiazol-4-yl)acetate (29)

White powder, m. p. = 204.0 °C, yield = 85%, ^{1}H NMR (DMSO-d_6, 300MHz): δ (ppm) 8.41 (s, 1H), 8.12 – 8.08 (m, 2H), 7.36 (t, J = 8.7 Hz, 3H), 5.39 (s br, 1H), 4.10 (q, J = 6.9 Hz, 2H), 3.94 – 3.88 (m, 1H), 3.53 (d, J = 11.5 Hz, 1H), 2.84 – 2.83 (m, 2H), 1.20 (t, J = 7.1 Hz, 3H); ^{13}C NMR (75MHz, DMSO-d_6) δ = 170.0, 168.6, 164.4 ($^{1}J_{C\text{-}F}$ = 248.1 Hz), 149.9, 131.5 ($^{3}J_{C\text{-}F}$ = 8.79 Hz), 130.1, 116.4 ($^{2}J_{C\text{-}F}$ = 21.73 Hz), 61.1, 56.7, 32.4, 32.3, 14.4. HRMS (ESI-FTMS Mass (m/z): calcd for $C_{14}H_{17}FN_3O_2S$ $[M + H]^+$ 310.102, found 310.1024.

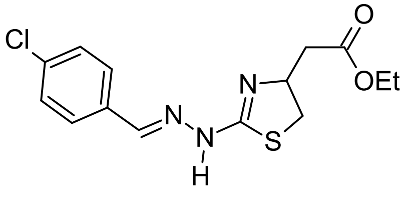

Ethyl (2-{(2*E*)-2-[(4-chlorophenyl)methylidene]hydrazinyl}-4,5-dihydro-1,3-thiazol-4-yl)acetate (30)

White powder, m. p. = 210.1 °C, yield = 70%, ^{1}H NMR (DMSO-d_6, 300MHz): δ (ppm) 10.22 (s, 1H), 8.41 (s, 1H), 8.07 (d, J = 7.2 Hz, 2H), 7.59 (d, J = 7.1 Hz, 2H), 5.42 – 5.40 (m, 1H), 4.10 (q, J = 6.5 Hz, 2H), 3.96 – 3.90 (m, 1H), 3.55 (d, J =11.5 Hz, 1H), 2.85 (d, J = 5.7 Hz, 2H), 1.21 (t, J = 5.8 Hz, 3H); ^{13}C NMR (75MHz, DMSO-

d_6) δ = 169.9, 169.1, 150.0, 136.6, 132.3, 130.8, 129.3, 61.1, 56.8, 32.6, 32.2, 14.4. HRMS (ESI-FTMS Mass (m/z): calcd for $C_{14}H_{17}ClN_3O_2S$ $[M + H]^+$ 328.0696, found 328.0700.

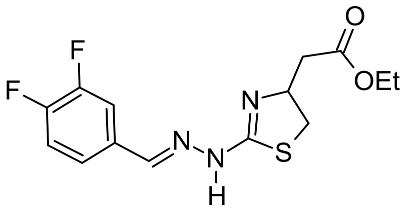

Ethyl (2-{(2*E*)-2-[(3,4-difluorophenyl)methylidene]hydrazinyl}-4,5-dihydro-1,3-thiazol-4-yl)acetate (31)

Brownish powder, m. p. = 169.9 °C, yield = 86%, ^{1}H NMR (DMSO-d_6, 300MHz): δ (ppm) 10.22 (s, 1H), 8.27 (t, J = 8.3 Hz, 1H), 7.77 (s, 1H), 7.64-7.55 (m, 1H), 5.35 – 5.33 (m, 1H), 4.07 (q, J = 6.9 Hz, 2H), 3.90 (dd, J = 3.6, 11.2 Hz, 1H), 3.91 – 3.88 (m, 1H), 2.84 – 2.82 (m, 2H), 1.18 (t, J= 7.11 Hz, 3H); ^{13}C NMR (75MHz, DMSO-d_6) δ = 169.9, 169.3, 153.5 ($^1J_{C-F}$ = 250.4 Hz, $^3J_{C-F}$ = 12.6 Hz), 150.0 ($^1J_{C-F}$ = 258.0 Hz, $^3J_{C-F}$ = 13.4 Hz), 148.6, 131.2, 127.6, 118.5 ($^2J_{C-F}$ = 17.6 Hz), 116.5 ($^2J_{C-F}$ = 18.8 Hz), 61.1, 56.7, 32.6, 32.0, 14.4. HRMS (ESI-FTMS Mass (m/z): calcd for $C_{14}H_{16}F_2N_3O_2S$ $[M + H]^+$ 328.0926, found 328.0929.

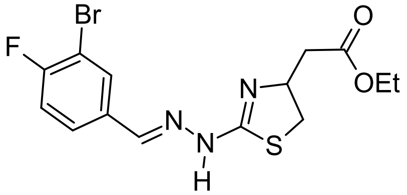

Ethyl (2-{(2*E*)-2-[(3-bromo-4-fluorophenyl)methylidene]hydrazinyl}-4,5-dihydro-1,3-thiazol-4-yl)acetate (32)

White powder, m. p. = 140.1 °C, yield = 80%, ^{1}H NMR (DMSO-d_6, 300MHz): δ (ppm) 10.28 (s, 1H), 8.52 (d, J = 6.8 Hz, 1H), 8.39 (s, 1H), 8.05 – 8.01 (m, 1H), 7.54 (t, J = 6.7 Hz, 1H), 5.38 – 5.36 (m, 1H), 4.11 (q, J = 5.6 Hz, 2H), 3.93 (t, J = 10.5 Hz, 1H), 3.54 (d, J = 11.6 Hz, 1H), 2.85 – 2.83 (m, 2H), 1.20 (t, J = 4.8 Hz, 3H); ^{13}C NMR (75MHz, DMSO-d_6) δ = 169.9, 169.3, 160.0 ($^1J_{C-F}$ = 248.9 Hz), 148.7, 133.2, 131.6, 131.3, 117.6 ($^2J_{C-F}$ = 22.90 Hz), 109.5 ($^2J_{C-F}$ = 21.4 Hz), 61.1, 56.7, 32.6, 32.0, 14.4. HRMS (ESI-FTMS Mass (m/z): calcd for $C_{14}H_{16}BrFN_3O_2S$ $[M + H]^+$ 390.0105, found 390.0108.

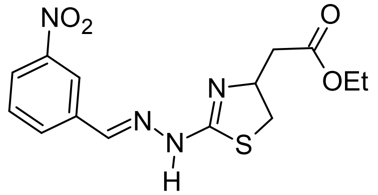

Ethyl (2-{(2*E*)-2-[(3-nitrophenyl)methylidene]hydrazinyl}-4,5-dihydro-1,3-thiazol-4-yl)acetate (33)

White powder, m. p. = 183.9 °C, yield = 84%, ^{1}H NMR (DMSO-d_6, 300MHz): δ (ppm) 10.38 (s, 1H), 8.87 (s, 1H), 8.56 (s, 1H), 8.50 (d, J = 7.38 Hz, 1H), 8.33 (dd, J = 1.6, 1.6 Hz, 1H), 7.81 (t, J = 7.5 Hz, 1H), 5.43 – 5.36 (m, 1H), 4.11 (q, J = 7.0 Hz, 2H), 3.96 (dd, J = 7.7, 11.6 Hz, 1H), 3.56 (d, J = 11.4 Hz, 1H), 2.90 – 2.87 (m, 2H), 1.21 (t, J = 6.72 Hz, 3H) ; ^{13}C NMR (75MHz, DMSO-d_6) δ = 170.0, 169.6, 149.2, 148.6, 135.1, 134.8, 130.8, 126.1, 123.7, 61.1, 56.9, 32.6, 31.9, 14.4. HRMS (ESI-FTMS Mass (m/z): calcd for $C_{14}H_{17}N_4O_4S$ $[M + H]^+$ 337.0965, found 337.0965.

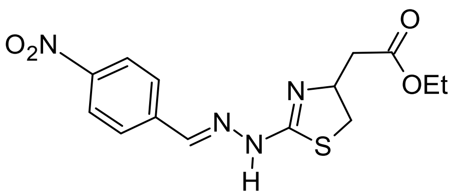

Ethyl (2-{(2*E*)-2-[(4-nitrophenyl)methylidene]hydrazinyl}-4,5-dihydro-1,3-thiazol-4-yl)acetate (34)

Yellowish powder, m. p. = 217.8 °C, yield = 86%, ^{1}H NMR (DMSO-d$_6$, 300MHz): δ (ppm) 8.44 (s, 1H), 8.32 (d, J = 7.62 Hz, 2H), 8.23 (d, J = 8.7 Hz, 2H), 5.35 (s br, 1H), 4.11 – 4.06 (m, 4H), 2.84 – 2.72 (m, 2H), 1.19 (t, J = 6.99 Hz, 3H); ^{13}C NMR (75MHz, DMSO-d$_6$) δ = 170.0, 168.6, 148.9, 147.6, 139.7, 129.7, 124.3, 61.2, 56.7, 32.3, 32.1, 14.4. HRMS (ESI-FTMS Mass (m/z): calcd for $C_{14}H_{17}N_4O_4S$ $[M + H]^+$ 337.0965, found 337.0969.

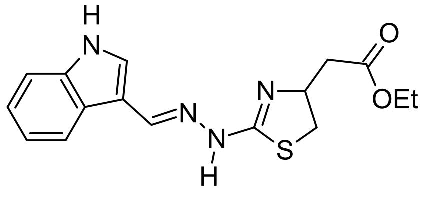

Ethyl (2-{(2*E*)-2-[(3*H*-indol-3-yl)methylidene]hydrazinyl}-4,5-dihydro-1,3-thiazol-4-yl)acetate (35)

Brownish powder, m. p. = 193.1 °C, yield = 75%, ^{1}H NMR (DMSO-d$_6$, 300MHz): δ (ppm) 11.93 (s, 1H), 8.56 (s, 1H), 8.27 (d, J = 7.3 Hz, 1H), 8.01 (s, 1H), 7.45 (d, J = 7.6 Hz, 1H), 7.23 – 7.14 (m, 2H), 5.42 (s br, 1H), 4.05 (s, 2H), 4.05 – 4.00 (m, 2H), 3.88 – 3.82 (m, 1H), 2.87 – 2.70 (m, 2H), 1.12 (t, J = 6.9 Hz, 3H); ^{13}C NMR (75MHz, DMSO-d$_6$) δ = 169.9, 166.2, 151.4, 137.5, 135.0, 124.1, 123.6, 122.8, 121.7, 112.5, 110.5, 61.1, 57.8, 33.0, 31.8, 14.3. HRMS (ESI-FTMS Mass (m/z): calcd for $C_{16}H_{19}N_4O_2S$ $[M + H]^+$ 331.1223, found 331.1226.

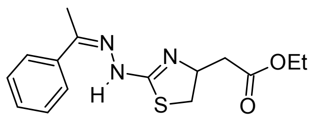

Ethyl {2-[(2*Z*)-2-(1-phenylethylidene)hydrazinyl]-4,5-dihydro-1,3-thiazol-4-yl}acetate (36)

White powder, m. p. = 166.0 °C, yield = 85%, ^{1}H NMR (DMSO-d$_6$, 300MHz): δ (ppm) 7.96 (d, J= 8.1 Hz, 2H), 7.61 – 7.58 (m, 1H), 7.54 – 7.49 (m, 2H), 484 – 4.80 (m, 1H), 3.94 – 3.88 (m, 3H), 3.55 – 3.51 (m, 2H), 2.88 – 2.77 (m, 2H), 2.44 (s, 3H), 1.07 (t, J= 7.1 Hz, 3H); ^{13}C NMR (75MHz, DMSO-d$_6$) δ = 180.5, 169.9, 166.0, 135.5, 132.7, 128.8, 128.4, 64.2, 61.0, 35.6, 31.7, 17.7, 14.2. HRMS (ESI-FTMS Mass (m/z): calcd for $C_{15}H_{20}N_3O_2S$ $[M + H]^+$ 306.1271, found 306.1275.

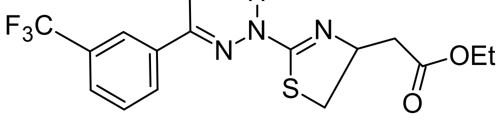

Ethyl {2-[(2*E*)-2-{2-[3-(trifluoromethyl)phenyl]propylidene}hydrazinyl]-4,5-dihydro-1,3-thiazol-4-yl}acetate (37)

White powder, m. p. = 95.1 °C, yield = 76%, ^{1}H NMR (DMSO-d$_6$, 300MHz): δ (ppm) 9.61 (bs a, 1H), 8.24-8.22 (m, 2H), 7.98 (d, J = 7.5 Hz, 1H), 7.75 (t, J = 7.8 Hz, 1H), 4.84 – 4.80 (m, 1H), 3.94 – 3.87 (m, 3H), 3.51 (dd, J = 6.0, 11.3 Hz, 1H), 2.92 – 2.83 (m, 2H), 2.50 (s, 3H), 1.07 (t, J = 7.0 Hz, 3H); ^{13}C NMR (75MHz, DMSO-d$_6$) δ = 179.0, 170.0, 165.9, 134.5 ($^1J_{C-F}$ = 310 Hz), 130.2, 129.6 ($^2J_{C-F}$ = 32.0 Hz), 128.8, 126.2, 124.6, 122.6, 64.1, 60.8, 35.8, 31.7, 18.0, 14.2. HRMS (ESI-FTMS Mass (m/z): calcd for $C_{16}H_{19}F_3N_3O_2S$ $[M + H]^+$ 374.1145, found 374.1144.

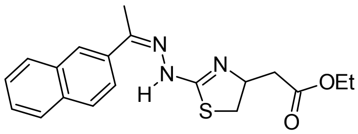

Ethyl (2-{(2*E*)-2-[2-(naphthalen-2-yl)propylidene]hydrazinyl}-4,5-dihydro-1,3-thiazol-4-yl)acetate (38)

White powder, m. p. = 191.0 °C, yield = 70%, ^{1}H NMR (DMSO-d_6, 300MHz): δ (ppm) 9.70 (br s, 1H), 8.60 (s, 1H), 8.09 – 8.07 (m, 2H), 8.00 – 7.98 (m, 2H), 7.62 (s, 2H), 4.87 (br s, 1H), 3.96 – 3.87 (m, 3H), 3.53 – 3.45 (m, 2H), 2.96-2.82 (m, Hz, 2H), 2.57 (s, 2H), 1.01 (t, J = 3.9 Hz, 3H); ^{13}C NMR (75MHz, DMSO-d_6) δ = 180.3, 169.9, 166.0, 134.9, 132.9, 132.6, 130.1, 129.6, 128.6, 128.1, 128.0, 127.3, 124.3, 64.3, 60.9, 35.7, 31.8, 17.8, 14.2. HRMS (ESI-FTMS Mass (m/z): calcd for $C_{19}H_{22}N_3O_2S$ $[M + H]^+$ 356.1427, found 356.143.

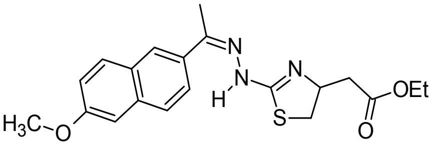

Ethyl (2-{(2*Z*)-2-[1-(6-methoxynaphthalen-2-yl)ethylidene]hydrazinyl}-4,5-dihydro-1,3-thiazol-4-yl)acetate (39)

White powder, m. p. = 216.2 °C, yield = 68%, ^{1}H NMR (DMSO-d_6, 300MHz): δ (ppm) 8.48 (s, 1H), 8.02 (dd, J = 8.7, 13.4 Hz, 2H), 7.88 (d, J = 8.7 Hz, 1H), 7.40 (s, 1H), 7.25 (dd, J = 2.1, 8.9 Hz, 1H), 4.82-4.77 (m, 1H), 3.91-3.85 (s, 6H), 3.52-3.50 (m, 1H), 2.85 (m, 2H), 2.50 (s, 3H), 1.03 (t, J = 7.0 Hz, 3H) ; ^{13}C NMR (75MHz, DMSO-d_6) δ = 179.1, 170.0, 165.2, 159.4, 136.7, 131.2, 130.7, 129.7, 127.9, 127.0, 124.8, 119.8, 106.4, 64.1, 60.9, 55.8, 35.9, 31.5, 17.4, 14.2. HRMS (ESI-FTMS Mass (m/z): calcd for $C_{20}H_{24}N_3O_3S$ $[M + H]^+$ 386.1533, found 386.1536.

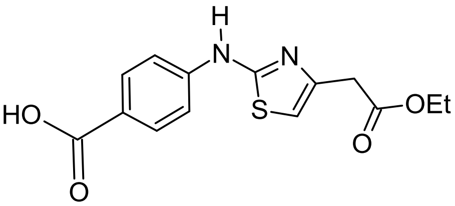

4-{[4-(2-Ethoxy-2-oxoethyl)-1,3-thiazol-2-yl]amino}benzoic acid (40)

White powder, m. p. = 273.0 °C, yield = 90%, ^{1}H NMR (DMSO-d_6, 300MHz): δ (ppm) 8.14 (d, J =8.4 Hz, 2H), 7.62 (d, J = 8.4 Hz, 2H), 7.07 (s, 1H), 3.85 (q, J = 7.0 Hz, 2H), 3.56 (s, 2H), 0.97 (t, J = 7.1 Hz, 3H); ^{13}C NMR (75MHz, DMSO-d_6) δ = 169.7, 168.2, 166.8, 136.3, 134.2, 133.7, 131.8, 129.5, 107.1, 61.4, 33.6, 14.1. HRMS (ESI-FTMS Mass (m/z): calcd for $C_{14}H_{15}N_2O_4S$ $[M + H]^+$ 307.0747, found 307.0751.

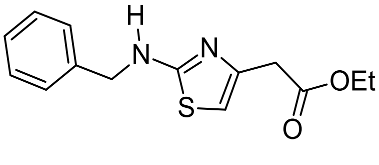

Ethyl [2-(benzylamino)-1,3-thiazol-4-yl]acetate (41)

White powder, m. p. = 220.0 °C, yield = 85%, ^{1}H NMR (DMSO-d_6, 300MHz): δ (ppm) 9.69 (br s, 1H), 7.41 – 7.33 (m, 3H), 7.08 (d, J = 7.0 Hz, 2H), 7.01 (s, 1H), 5.30 (s, 2H), 3.84 (q, J = Hz, 2H), 3.76 (s, 2H), 1.06 (t, J = 7.0 Hz, 3H); ^{13}C NMR (75MHz, DMSO-d_6) δ = 169.6, 168.3, 134.5, 133.7, 129.2, 128.5, 126.5, 107.4, 61.5, 48.7, 33.1, 14.2. HRMS (ESI-FTMS Mass (m/z): calcd for $C_{14}H_{17}N_2O_2S$ $[M + H]^+$ 277.1005, found 277.1008.

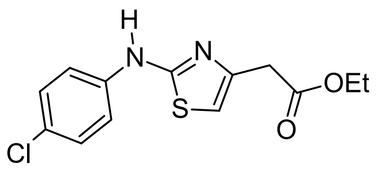

Ethyl {2-[(4-chlorophenyl)amino]-1,3-thiazol-4-yl}acetate (42)

Brownish solid, m. p. = 220.0 °C, yield = 99%, ^{1}H NMR (DMSO-d$_6$, 300MHz): 7.90 (d, J = 8.4 Hz, 2H), 7.56 (s, 1H), 7.53 (d, J = 8.4 Hz, 2H), 4.11 (q, J = 6.9 Hz, 2H), 3.87 (s, 2H), 1.18 (t, J = 7.0 Hz, 3); ^{13}C NMR (75MHz, CDCl$_3$) δ = 169.9, 166.0, 149.6, 135.4, 131.5, 128.6, 127.3, 115.9, 60.7, 36.6, 13.7. HRMS (ESI-FTMS Mass (m/z): calcd for $C_{13}H_{14}N_2O_2S$ $[M + H]^+$ 297.7799, found 297.7797.

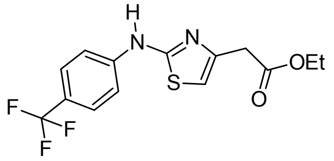

Ethyl (2-{[4-(trifluoromethyl)phenyl]amino}-1,3-thiazol-4-yl)acetate (43)

Viscous liquid, yield = 99%, ^{1}H NMR (CDCl$_3$, 300MHz): 8.06 (d, J = 8.2 Hz, 2H), 7.69 (d, J = 8.2 Hz, 2H), 7.29 (s, 1H), 4.23 (q, J = 7.1 Hz, 2H), 3.92 (s, 2H), 1.41 (t, J = 7.3 Hz, 3H); ^{13}C NMR (75MHz, CDCl$_3$) δ = 170.3, 165.9, 150.4, 136.5, 131.5 (^{2}J = 32.5 Hz), 126.7, 125.9 (^{3}J = 3.7 Hz), 117.2, 61.2, 37.0, 14.2. HRMS (ESI-FTMS Mass (m/z): calcd for $C_{14}H_{14}F_3N_2O_2S$ $[M + H]^+$ 331.3328, found 331.3328.

Ethyl (2-anilino-4,5-dihydro-1,3-thiazol-4-yl)acetate (1)

^{1}H NMR of **1**

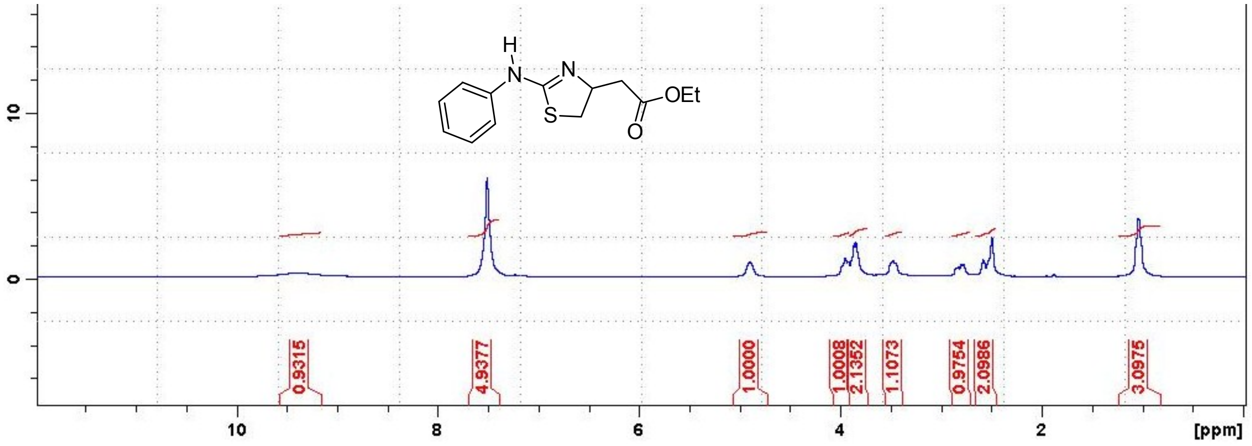

^{13}C NMR of **1**

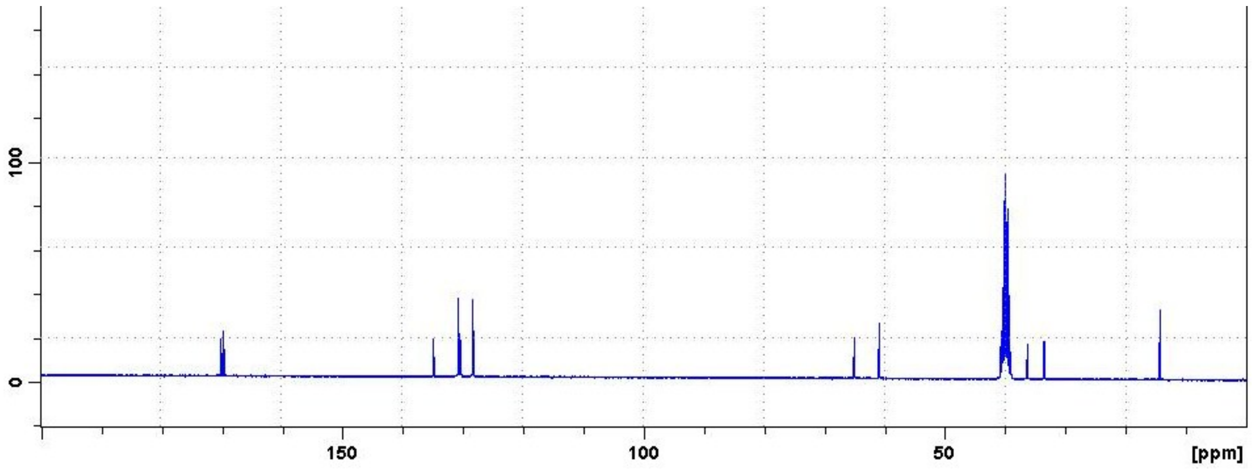

Ethyl [2-(4-methylanilino)-4,5-dihydro-1,3-thiazol-4-yl]acetate (2)

^{1}H NMR of **2**

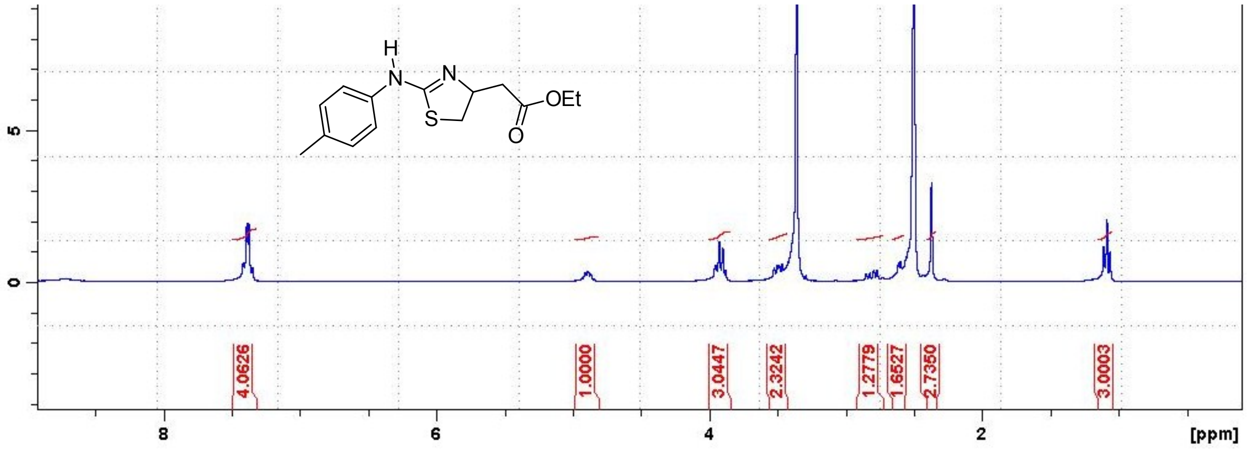

^{13}C NMR of **2**

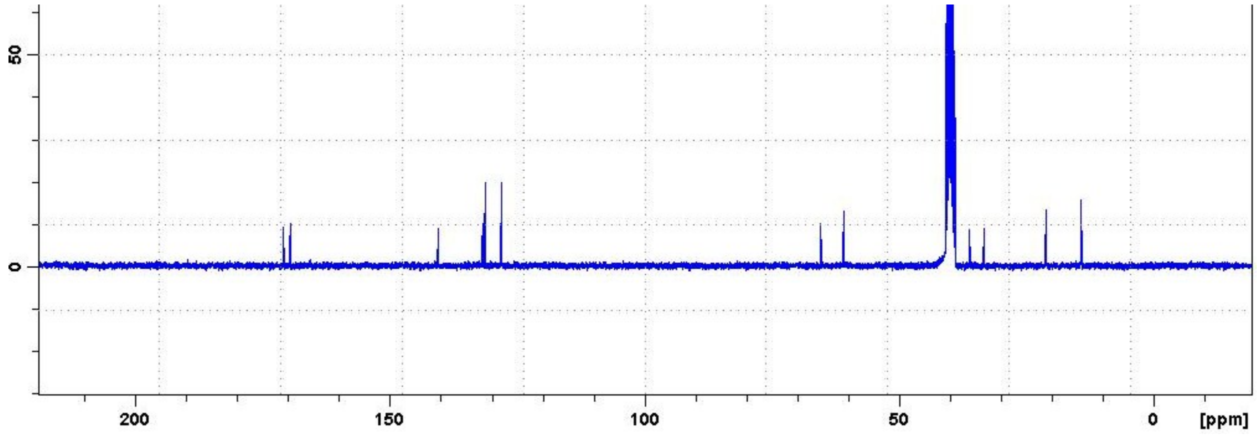

Ethyl [2-(2,4,6-trimethylanilino)-4,5-dihydro-1,3-thiazol-4-yl]acetate (3)

^{1}H NMR of **3**

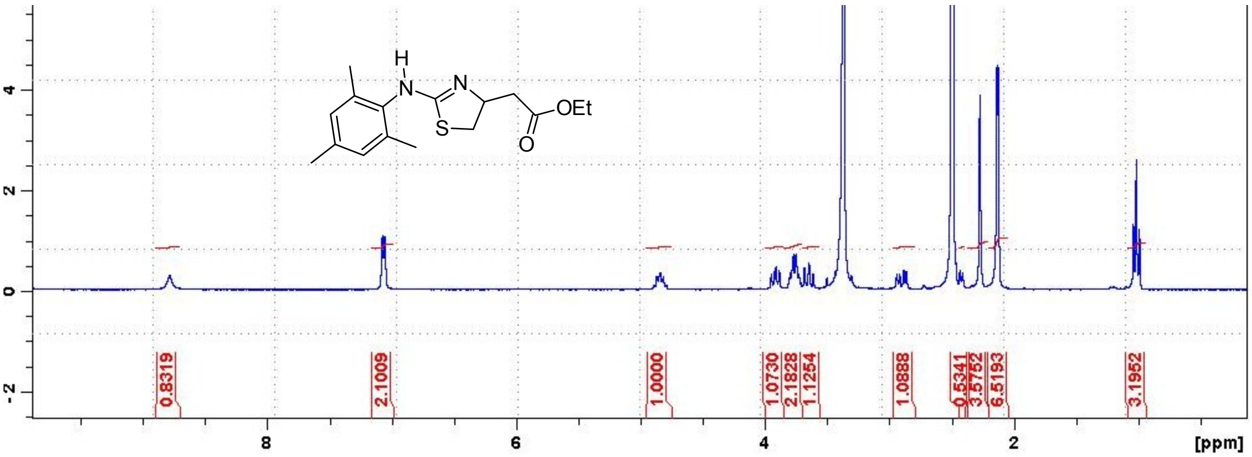

^{13}CNMR of **3**

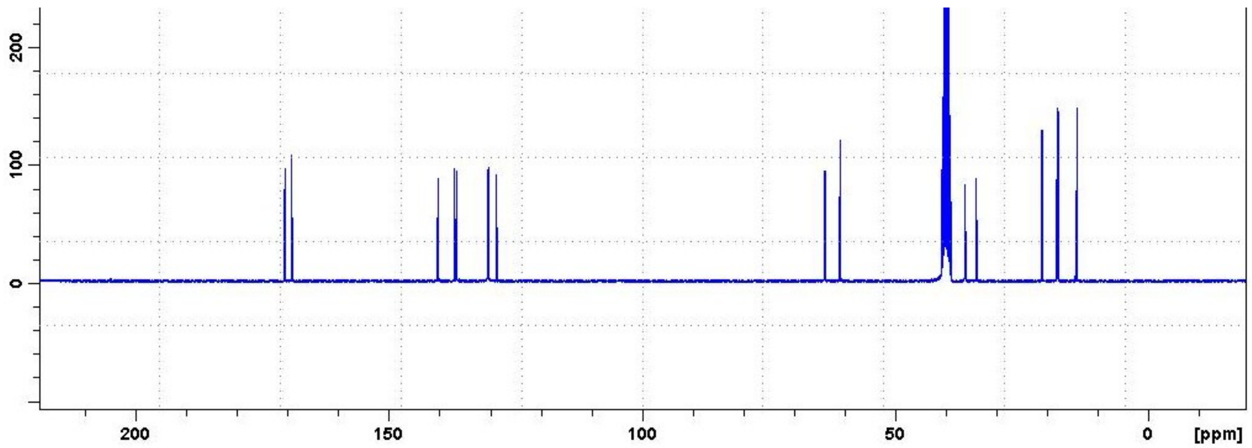

Ethyl [2-(2,4-dimethoxyanilino)-4,5-dihydro-1,3-thiazol-4-yl]acetate (4)

^{1}H NMR of **4**

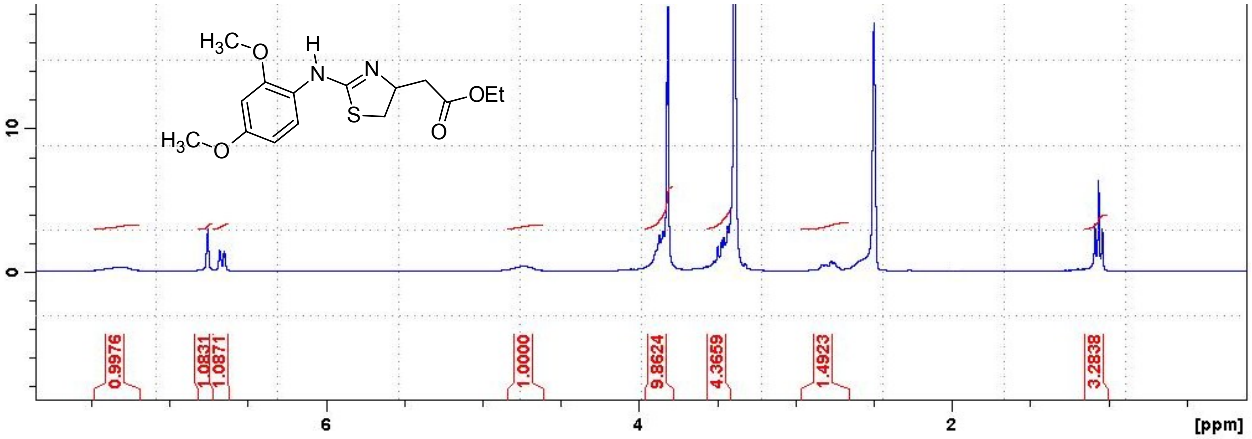

^{13}C NMR of **4**

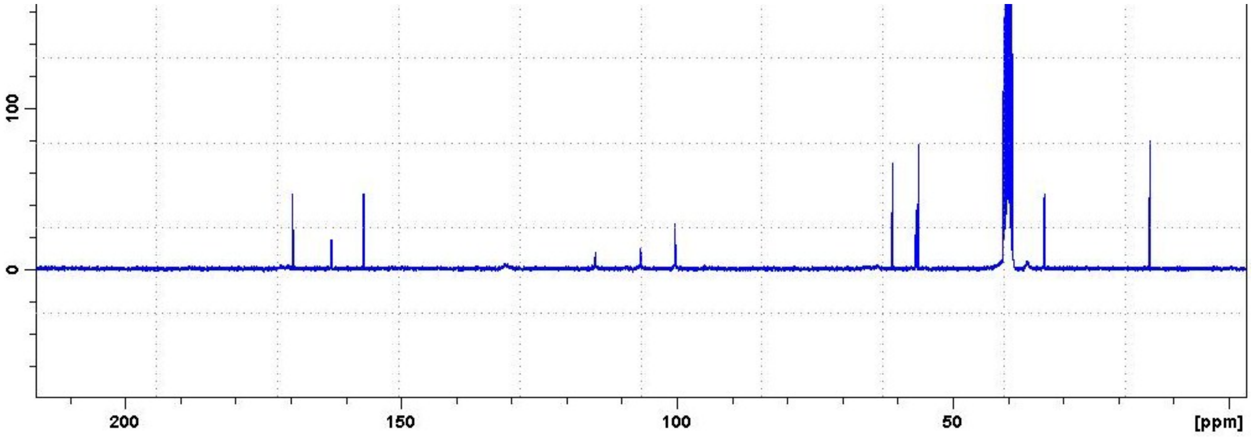

Ethyl [2-(2-fluoroanilino)-4,5-dihydro-1,3-thiazol-4-yl]acetate (5)

^{1}H NMR of **5**

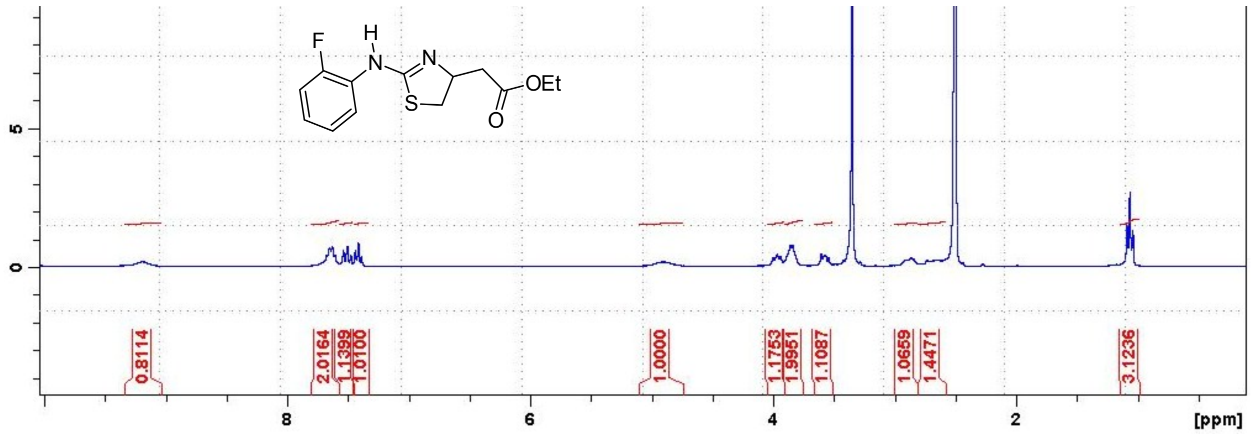

^{13}C NMR of **5**

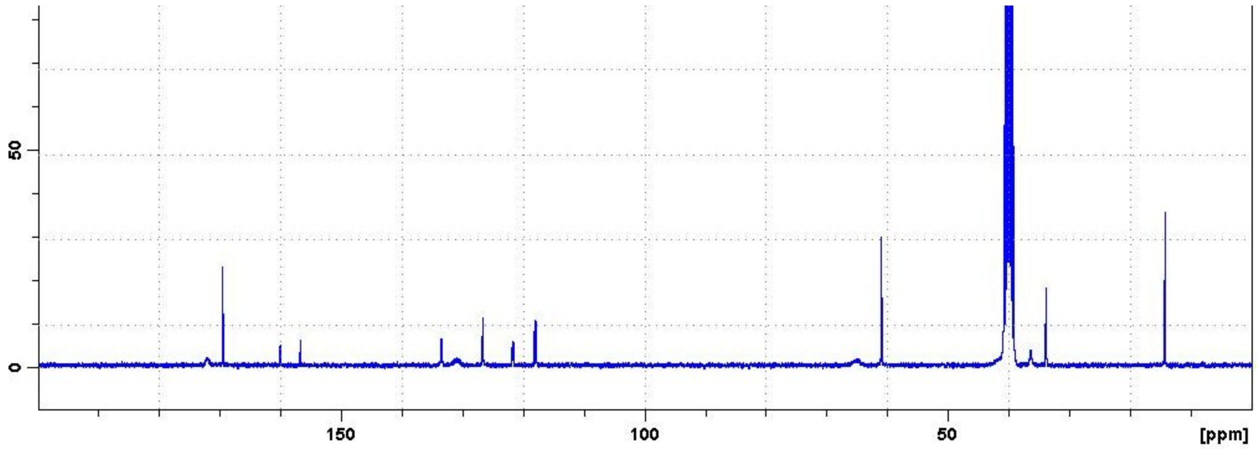

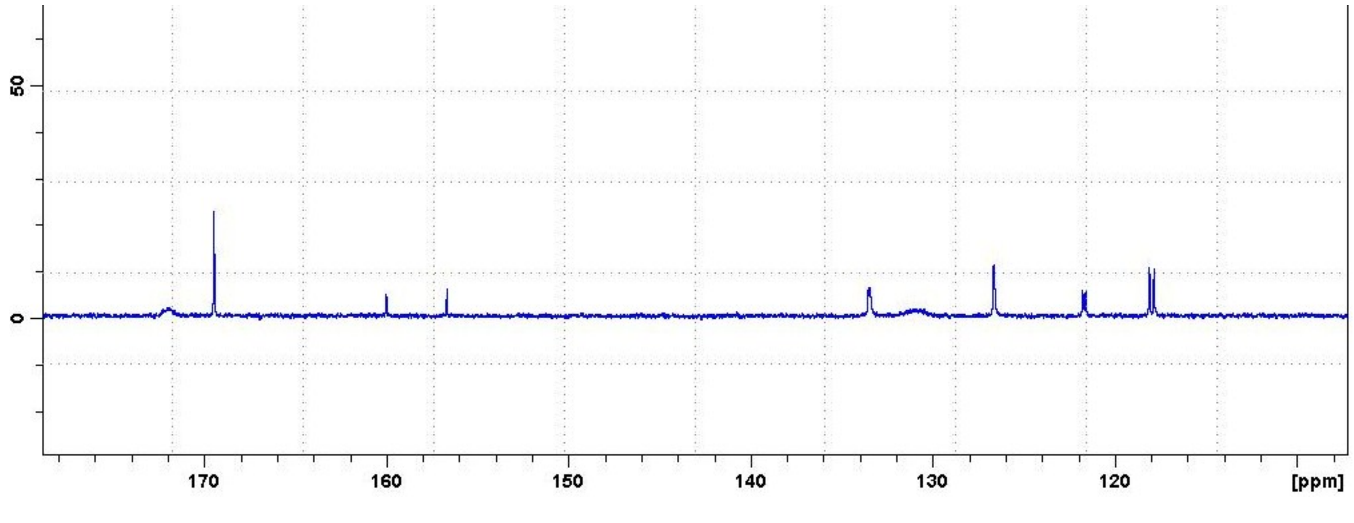

Ethyl [2-(3-fluoroanilino)-4,5-dihydro-1,3-thiazol-4-yl]acetate (6)

^{1}H NMR of **6**

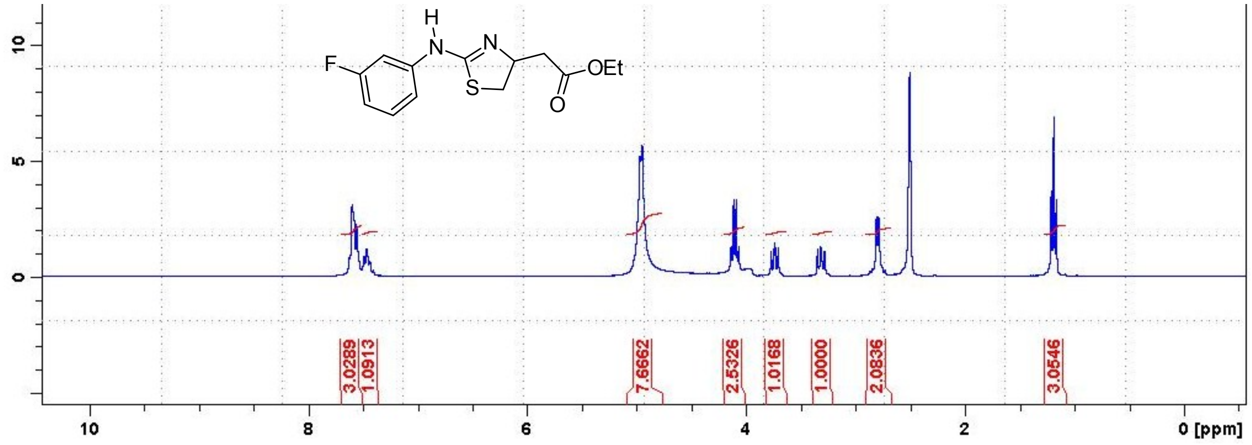

^{13}C NMR of **6**

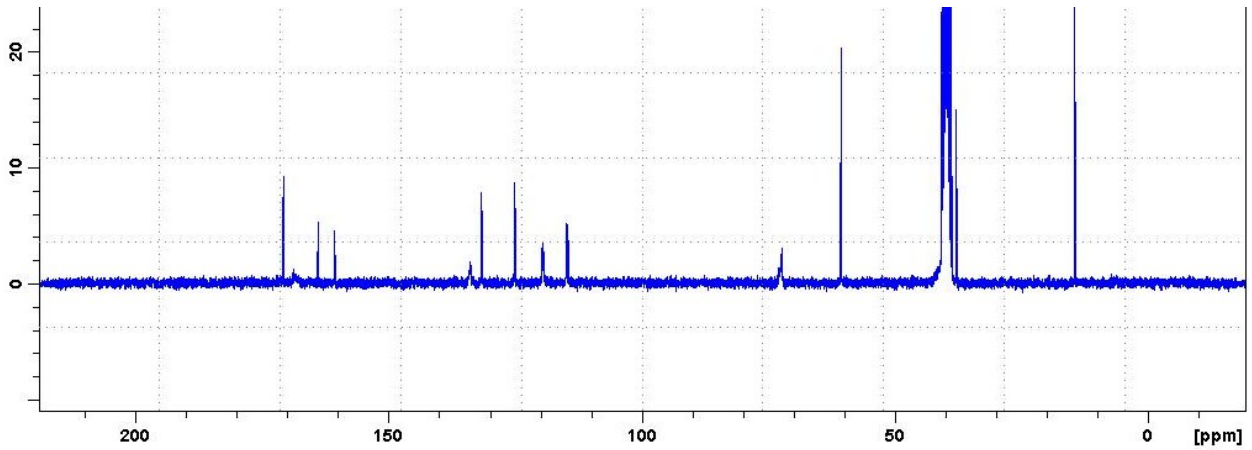

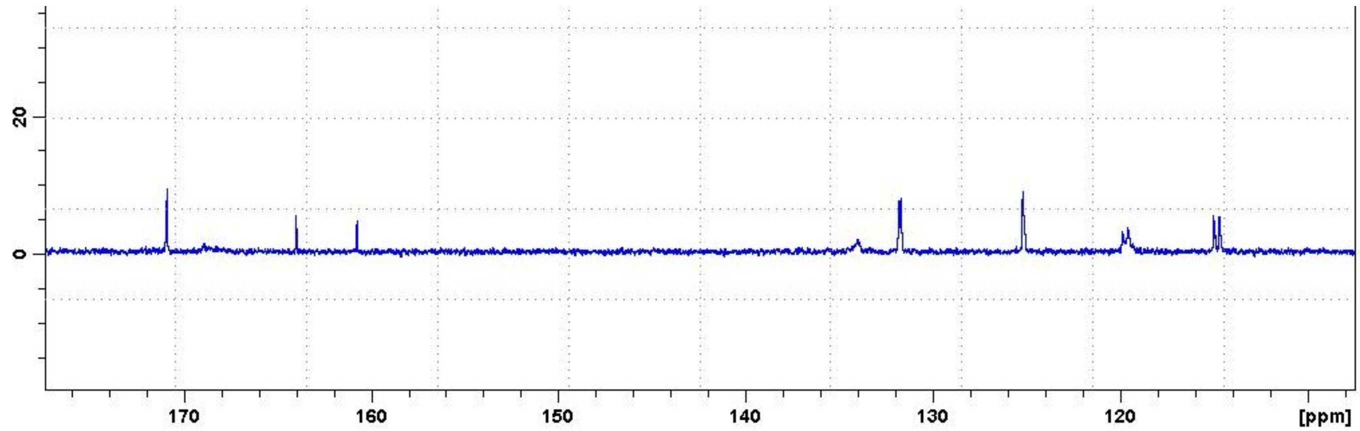

Ethyl [2-(4-chloroanilino)-4,5-dihydro-1,3-thiazol-4-yl]acetate (7)

^{1}H NMR of **7**

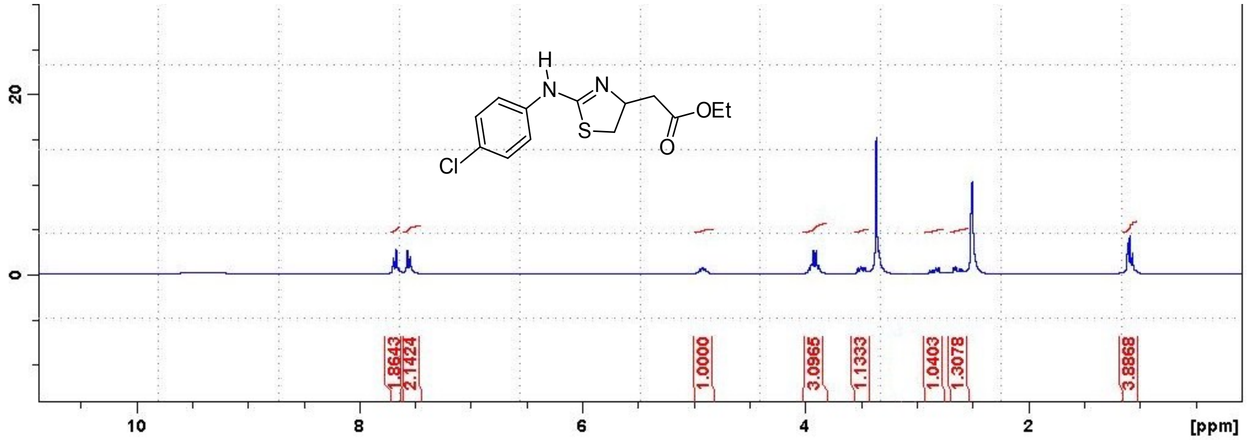

^{13}C NMR of **7**

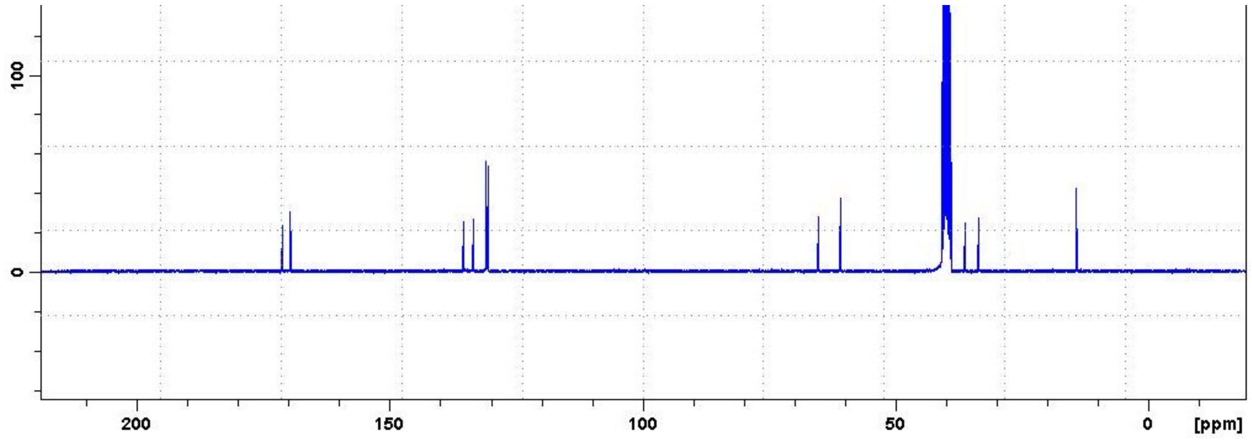

Ethyl {2-[3-(trifluoromethyl)anilino]-4,5-dihydro-1,3-thiazol-4-yl}acetate (8)

^{1}H NMR of **8**

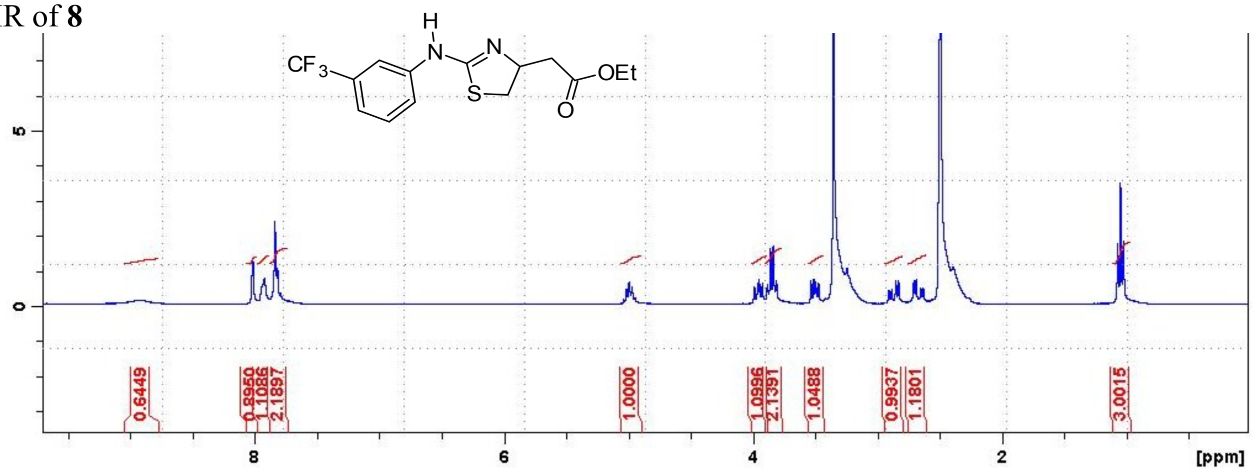

^{13}C NMR of **8**

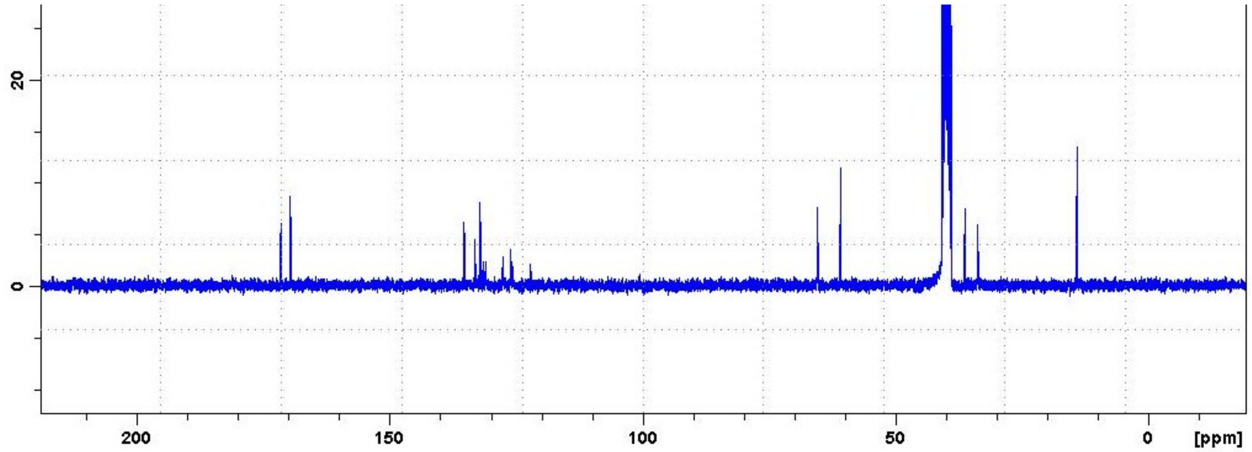

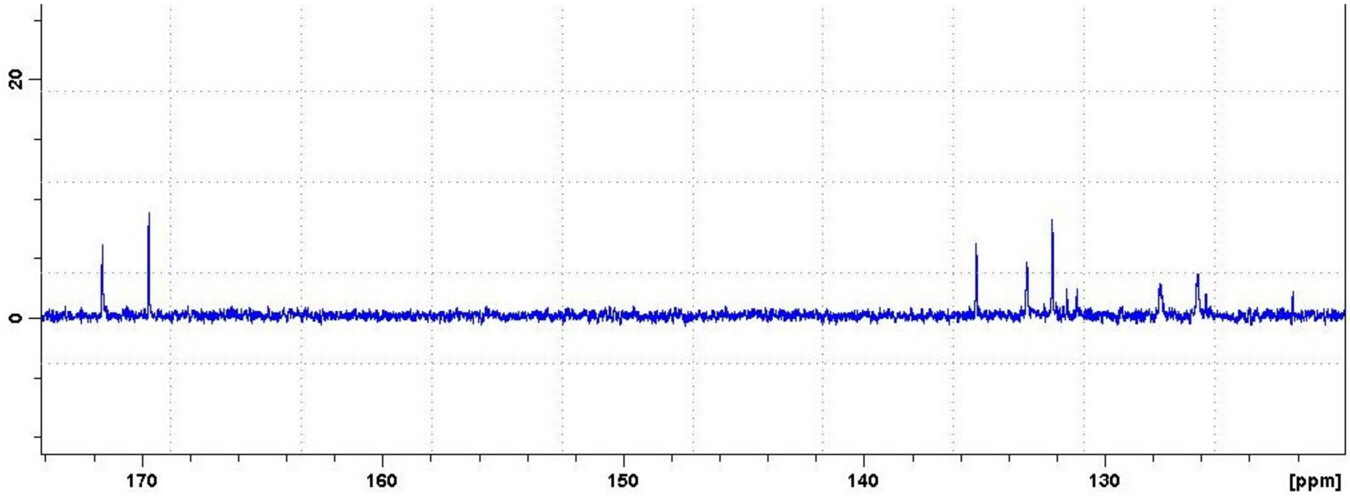

Ethyl {2-[3,5-bis(trifluoromethyl)anilino]-4,5-dihydro-1,3-thiazol-4-yl}acetate (9)

^{1}H NMR of **9**

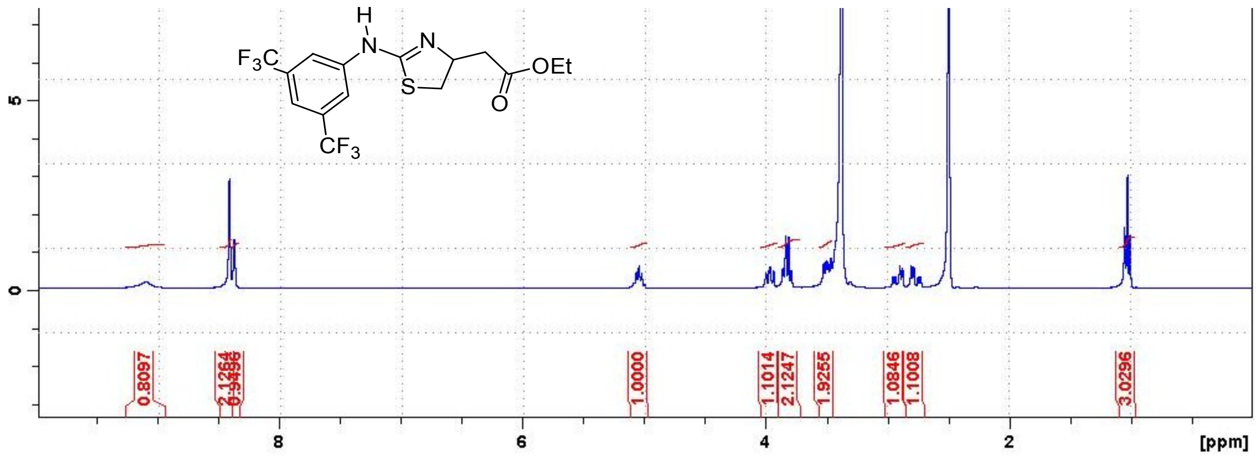

^{13}C NMR of **9**

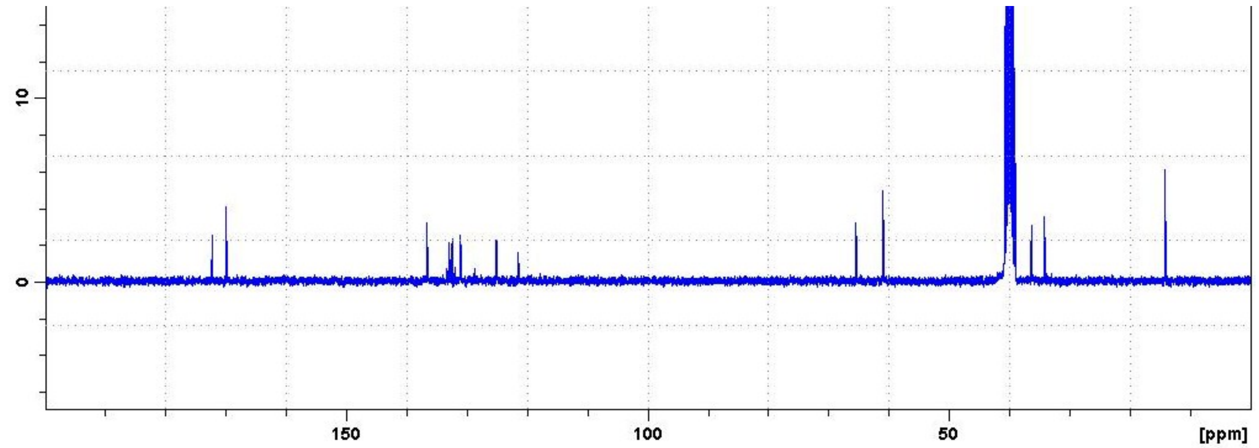

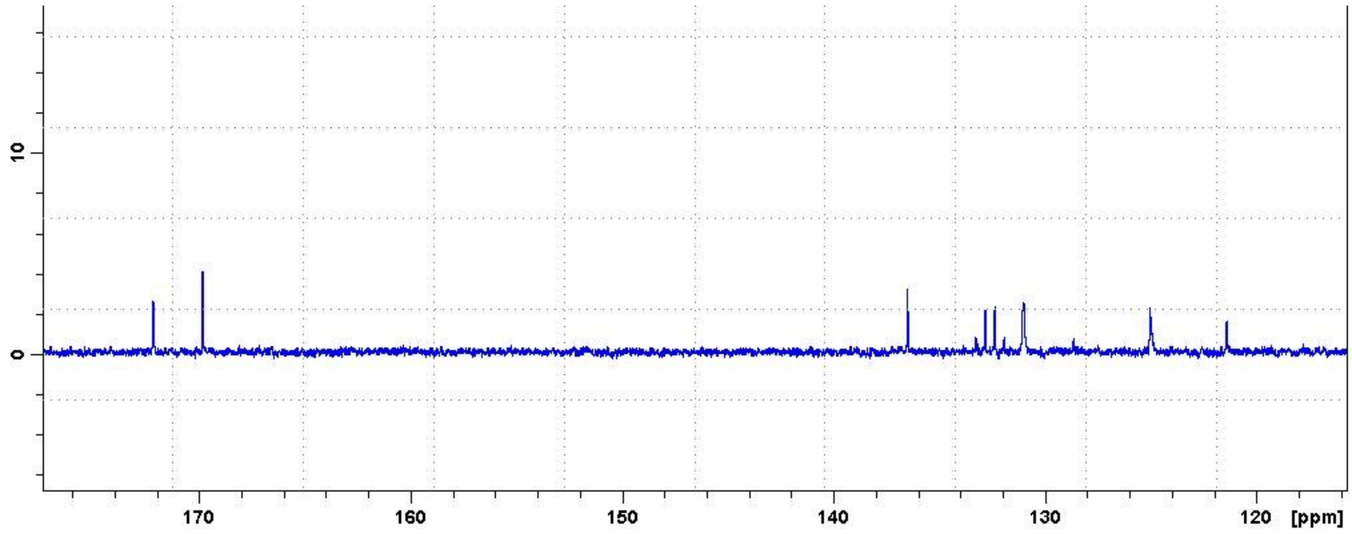

4-{[4-(2-Ethoxy-2-oxoethyl)-4,5-dihydro-1,3-thiazol-2-yl]amino}benzoic acid (10)

^{1}H NMR of **10**

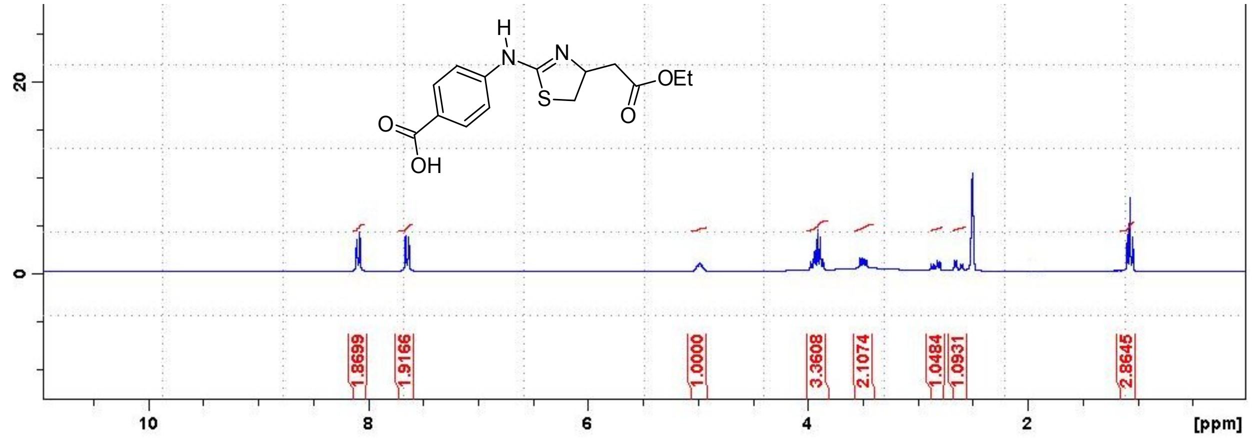

^{13}C NMR of **10**

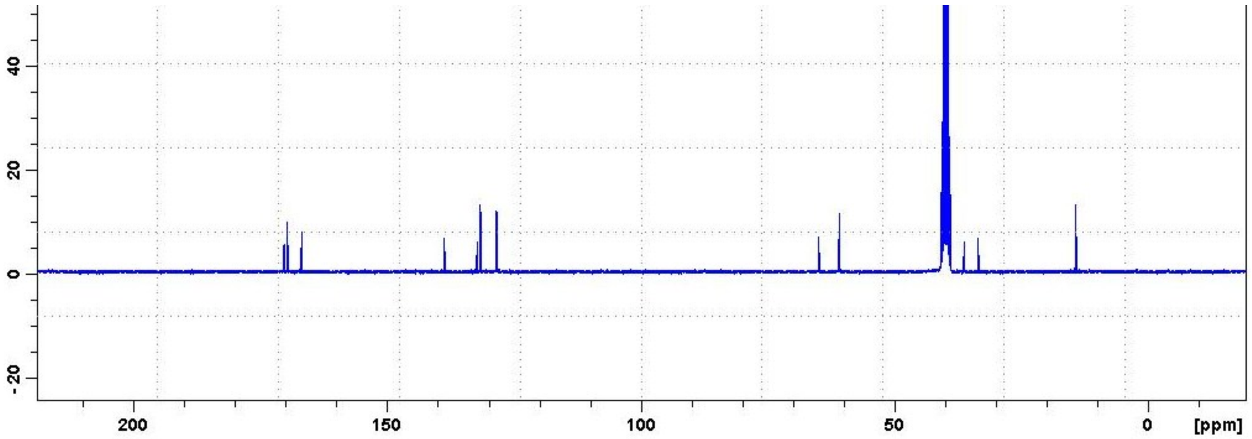

Ethyl [2-(benzylamino)-4,5-dihydro-1,3-thiazol-4-yl]acetate (11)

^{1}H NMR of **11**

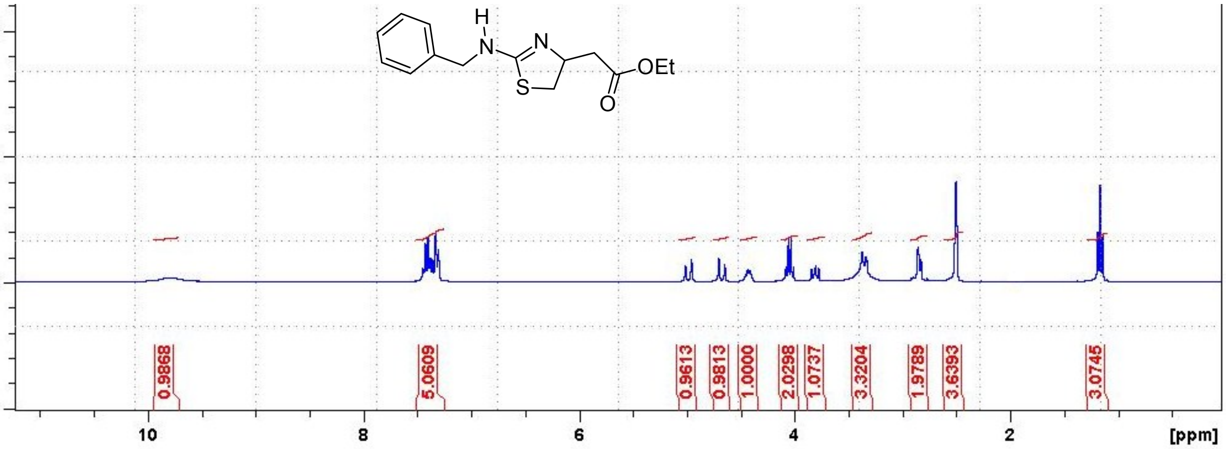

^{13}C NMR of **11**

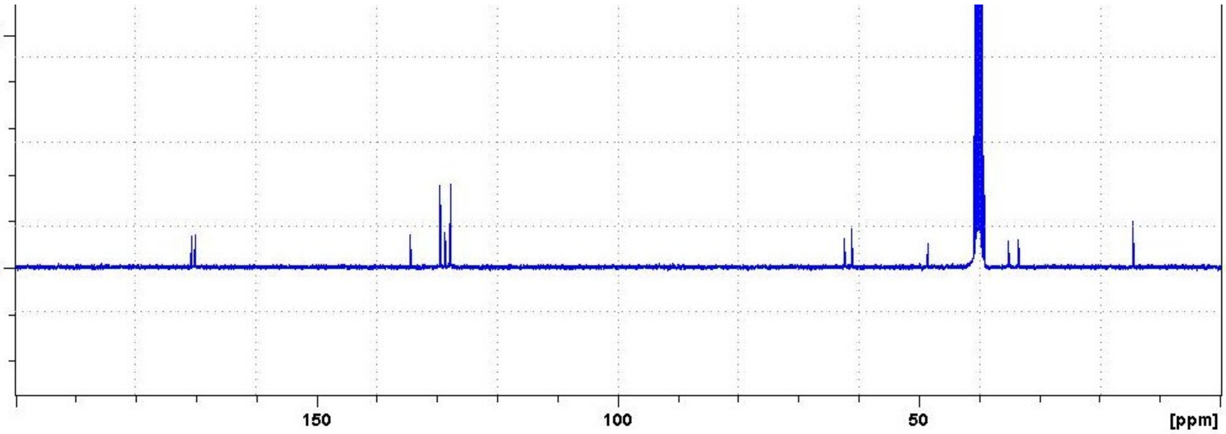

Ethyl {2-[(prop-2-en-1-yl)amino]-4,5-dihydro-1,3-thiazol-4-yl}acetate (12)

^{1}H NMR of **12**

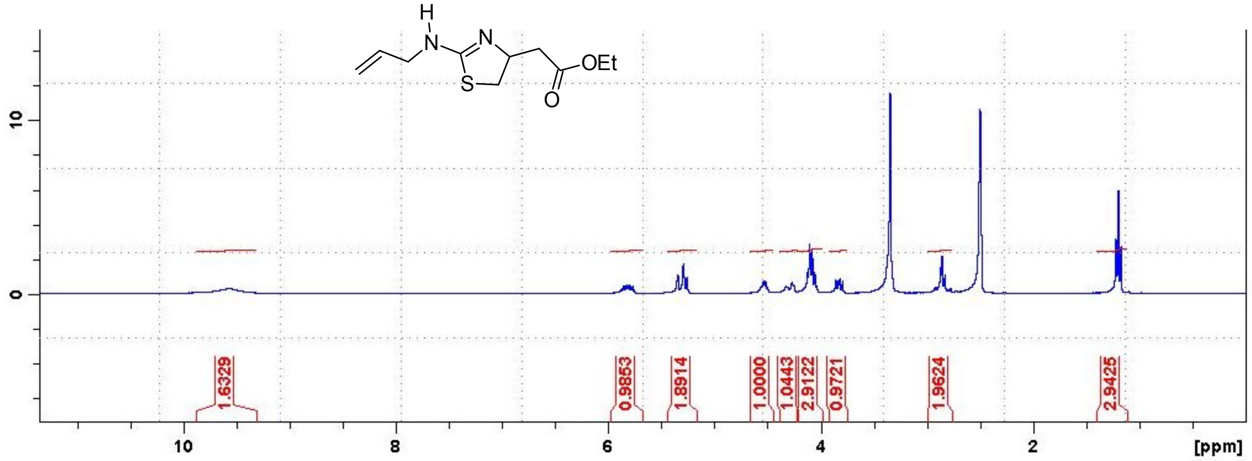

^{13}C NMR of **12**

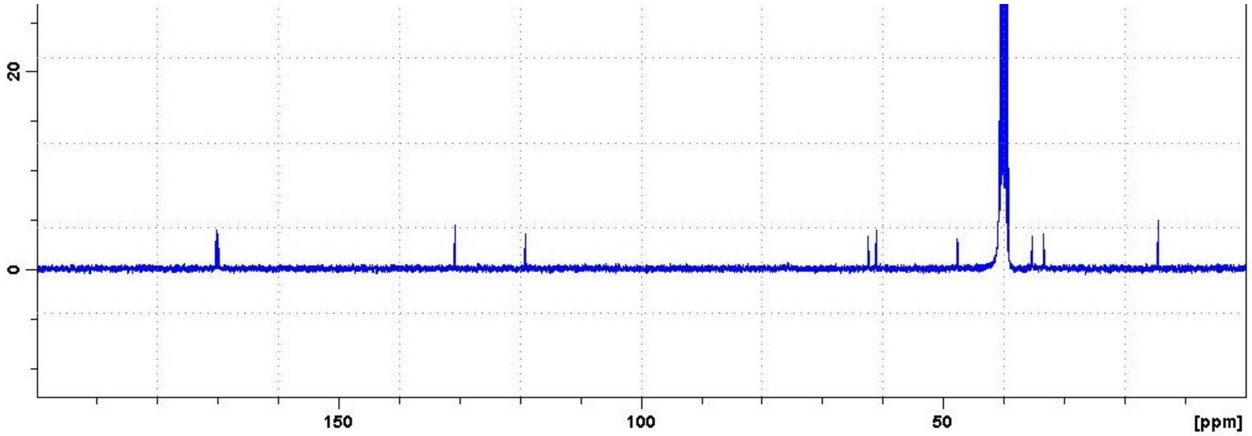

Ethyl (2-amino-4,5-dihydro-1,3-thiazol-4-yl)acetate (13)

^{1}H NMR of **13**

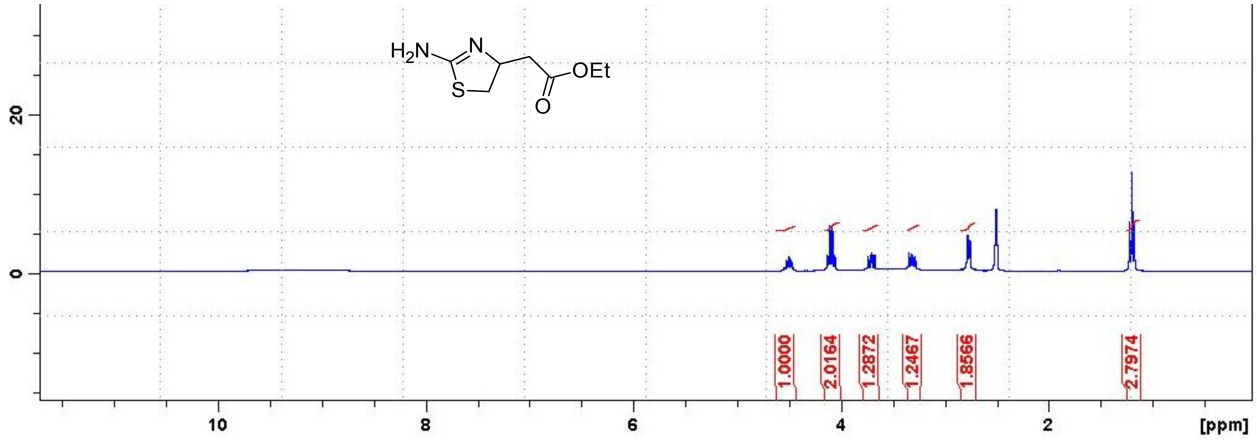

^{13}C NMR of **13**

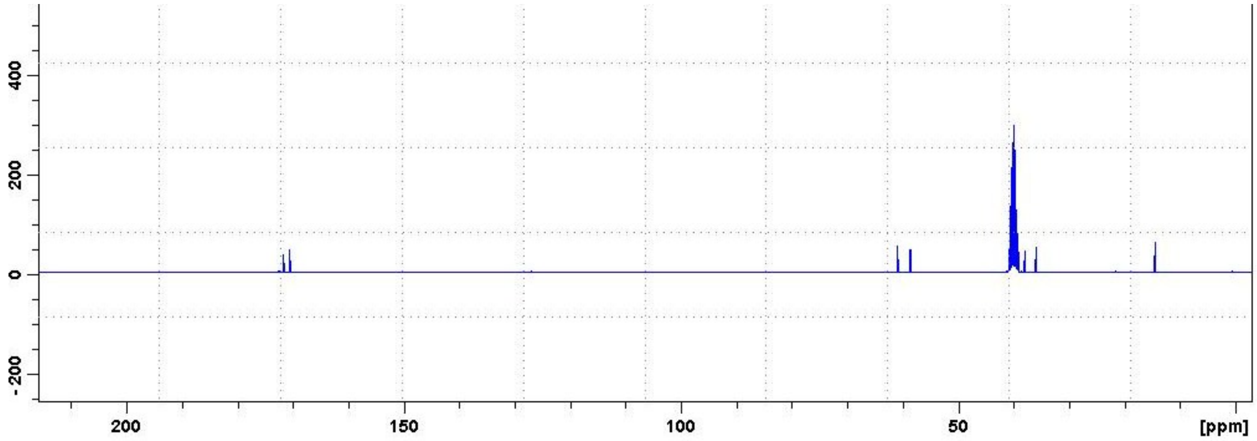

Ethyl [2-(3-methoxyphenyl)-4,5-dihydro-1,3-thiazol-4-yl]acetate (14)

^{1}H NMR of **14**

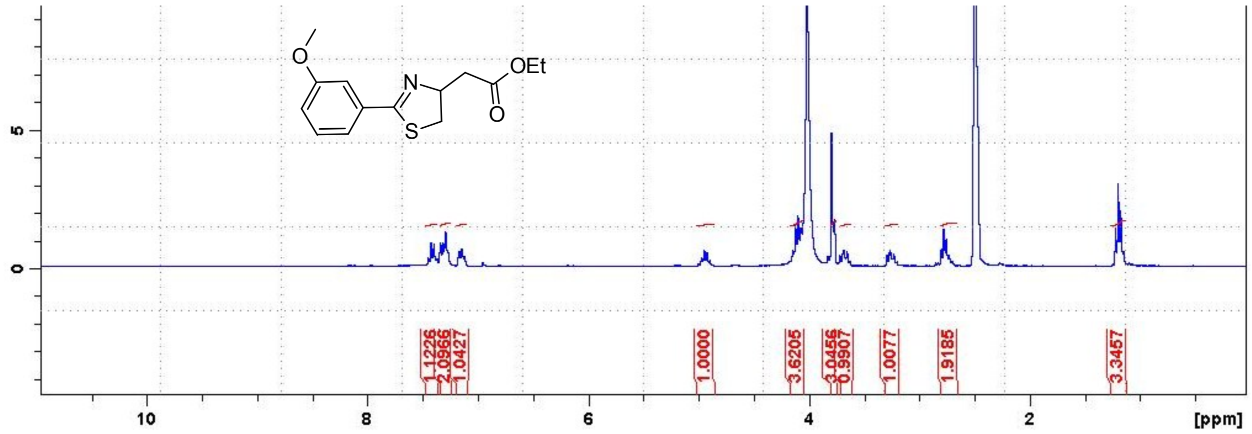

^{13}C NMR of **14**

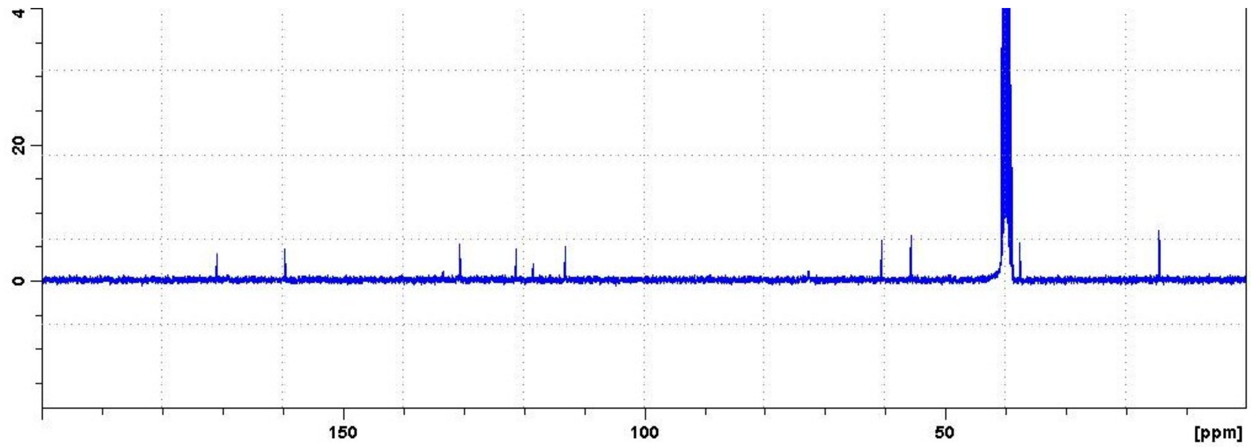

Ethyl [2-(2-hydroxyphenyl)-4,5-dihydro-1,3-thiazol-4-yl]acetate (15)

^{1}H NMR of **15**

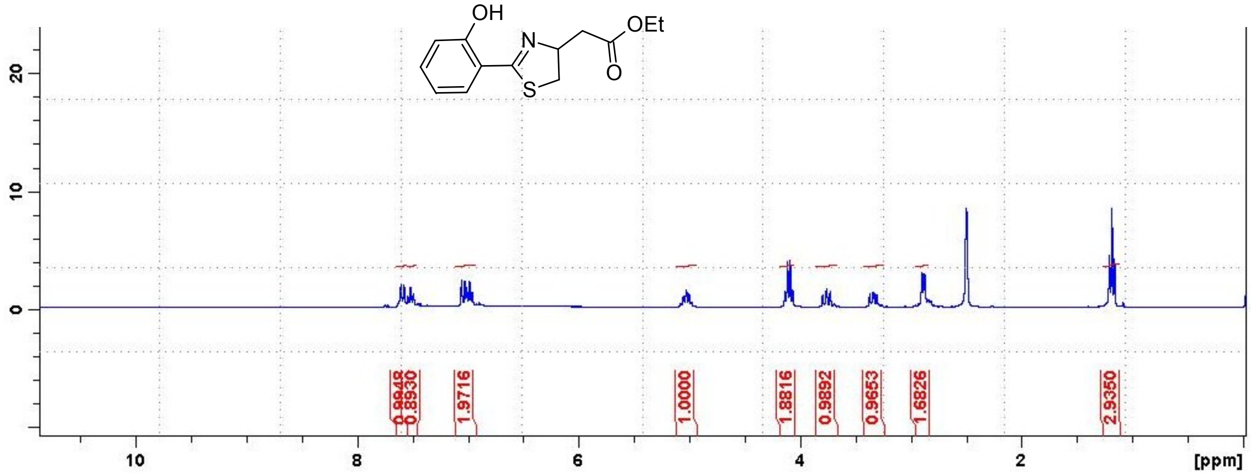

^{13}C NMR of **15**

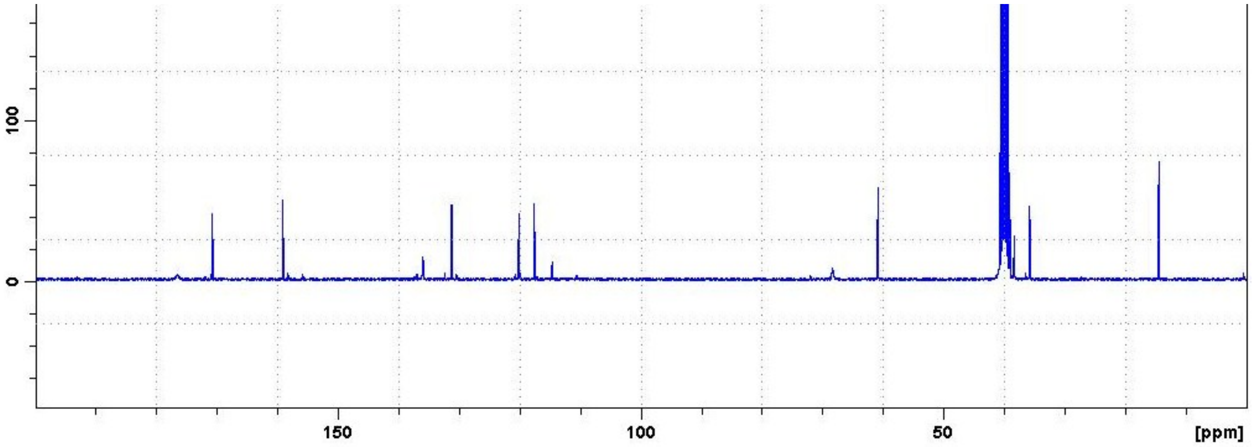

Ethyl [2-(4-hydroxyphenyl)-4,5-dihydro-1,3-thiazol-4-yl]acetate (16)

^{1}H NMR of **16**

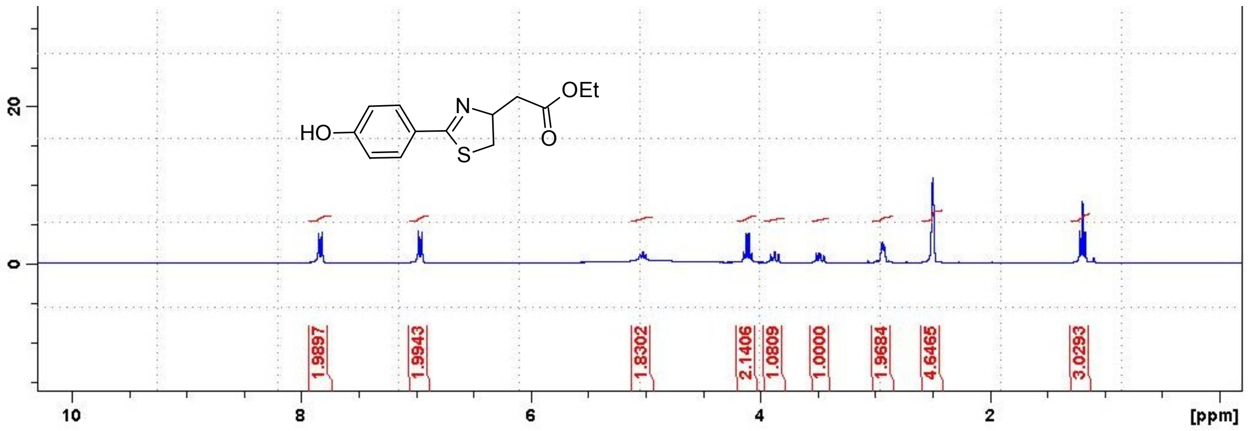

^{13}C NMR of **16**

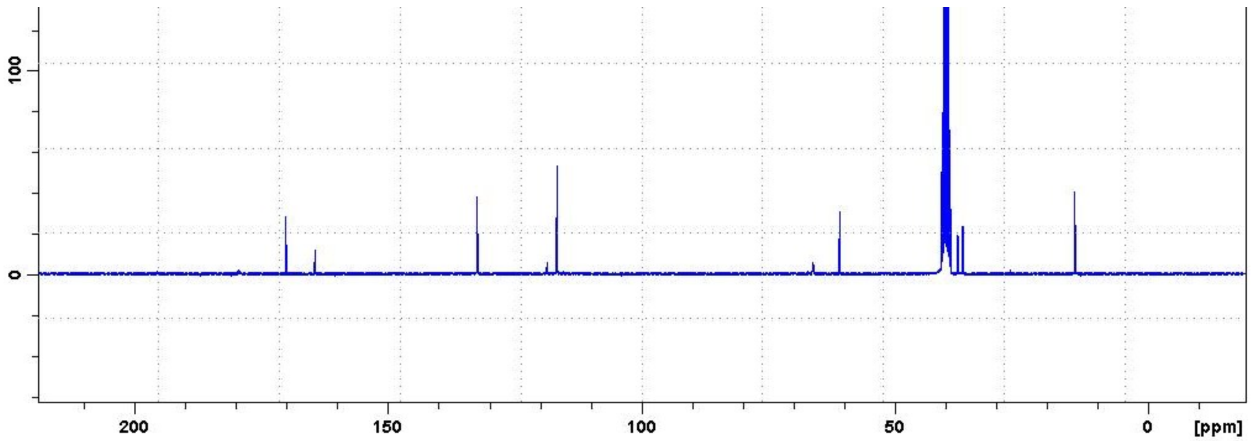

Ethyl [2-(4-methylphenyl)-4,5-dihydro-1,3-thiazol-4-yl]acetate (17)

^{1}H NMR of **17**

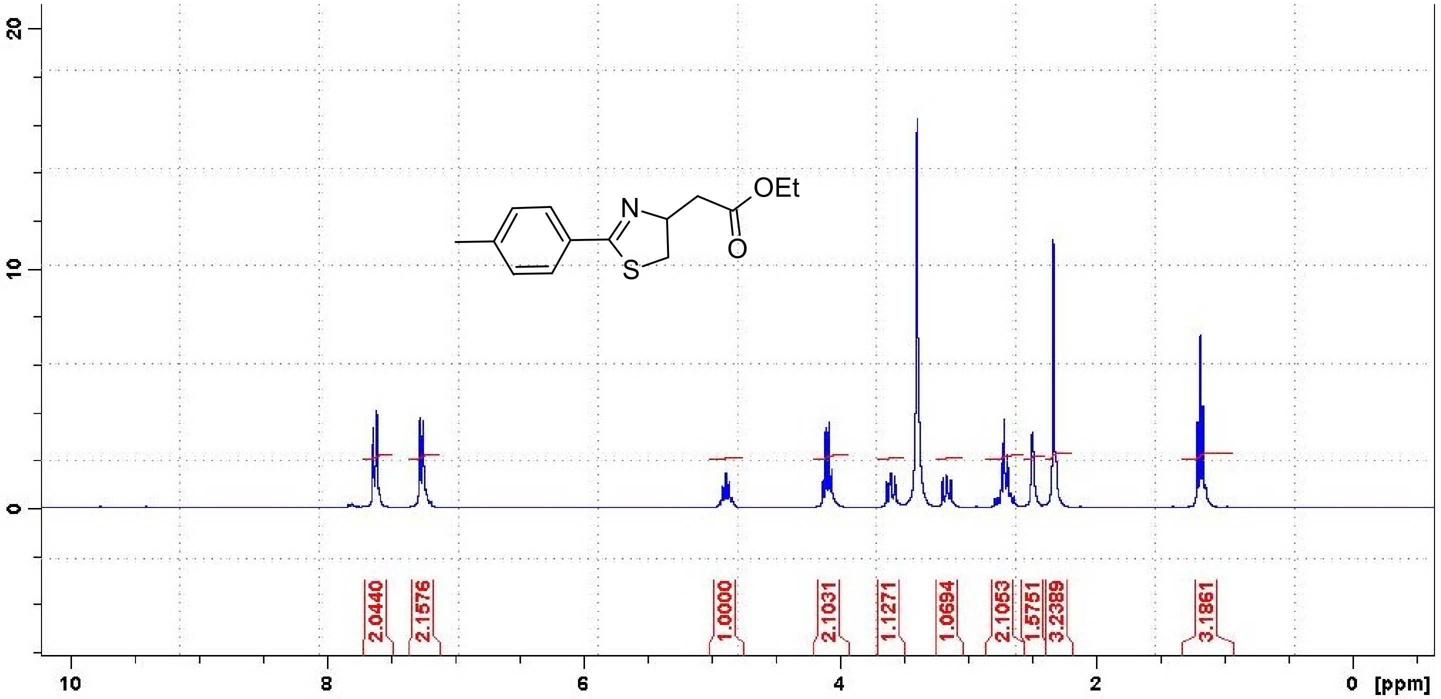

^{13}C NMR of **17**

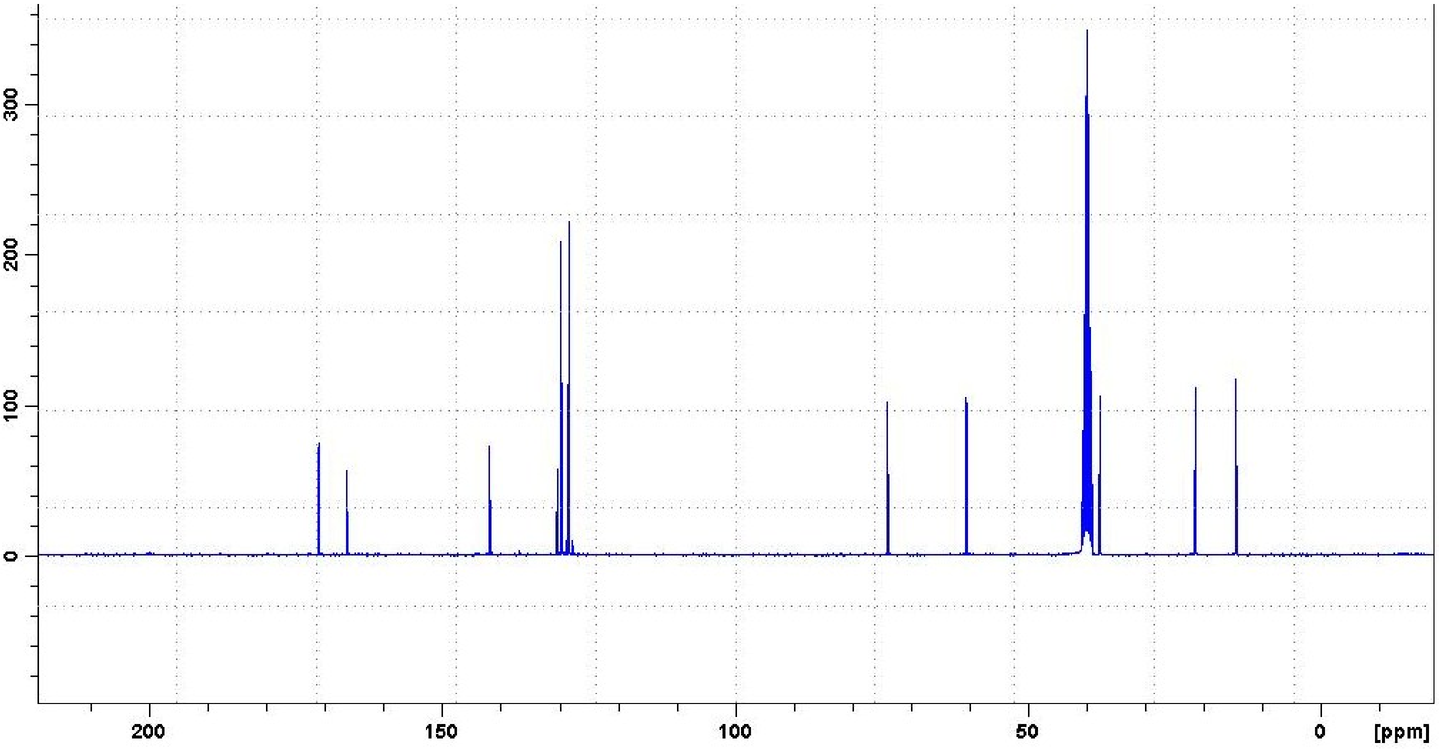

Ethyl [2-(4-fluorophenyl)-4,5-dihydro-1,3-thiazol-4-yl]acetate (18)

^{1}H NMR of **18**

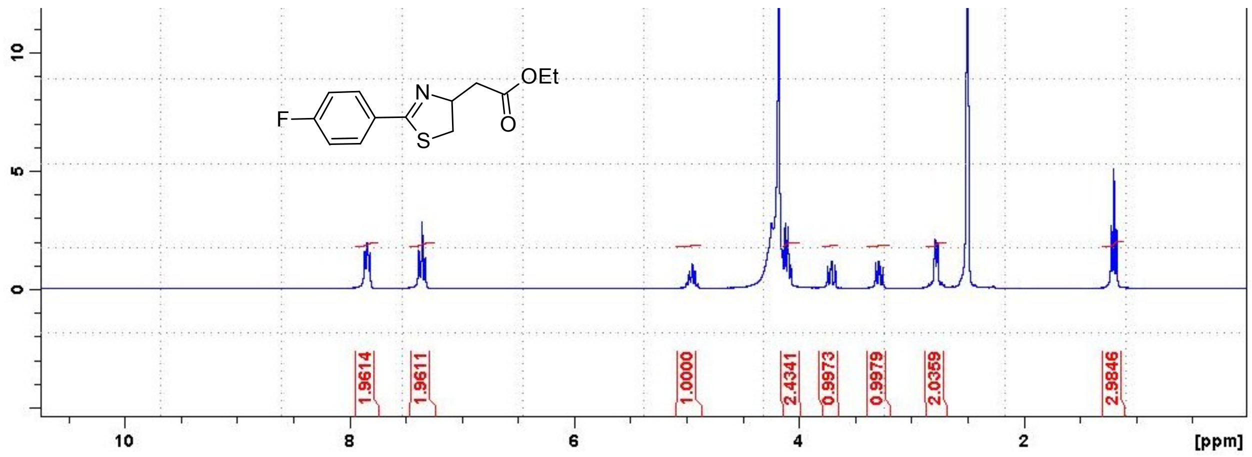

^{13}C NMR of **18**

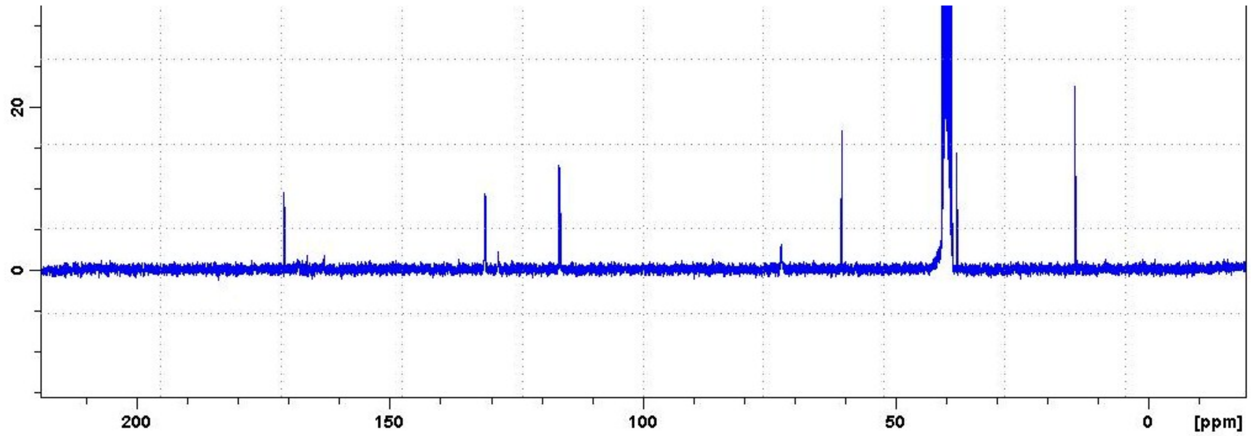

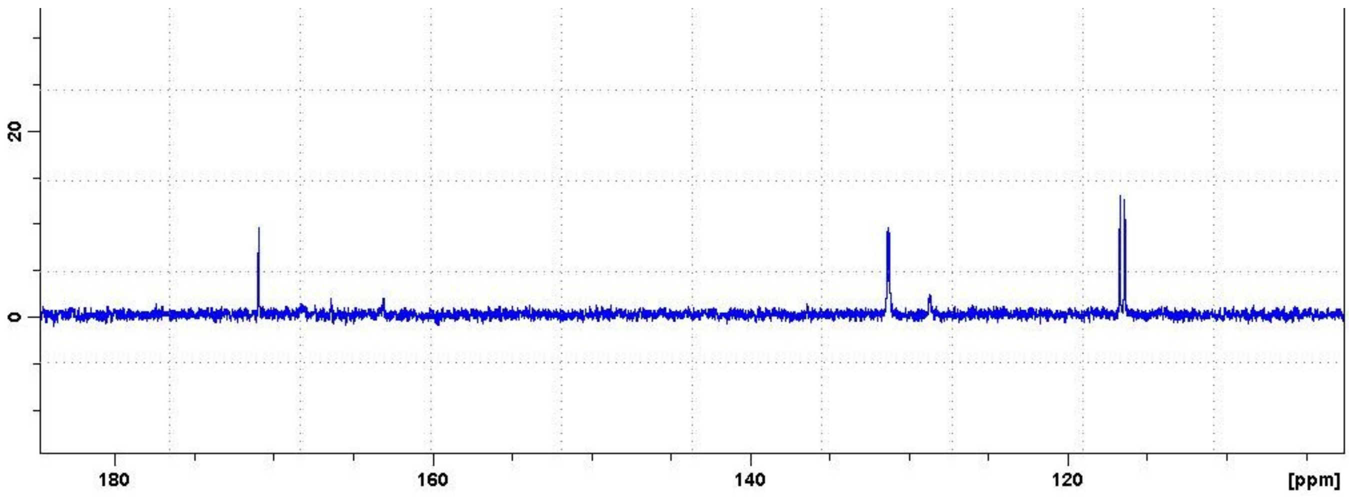

Ethyl [2-(3-chlorophenyl)-4,5-dihydro-1,3-thiazol-4-yl]acetate (19)

^{1}H NMR of **19**

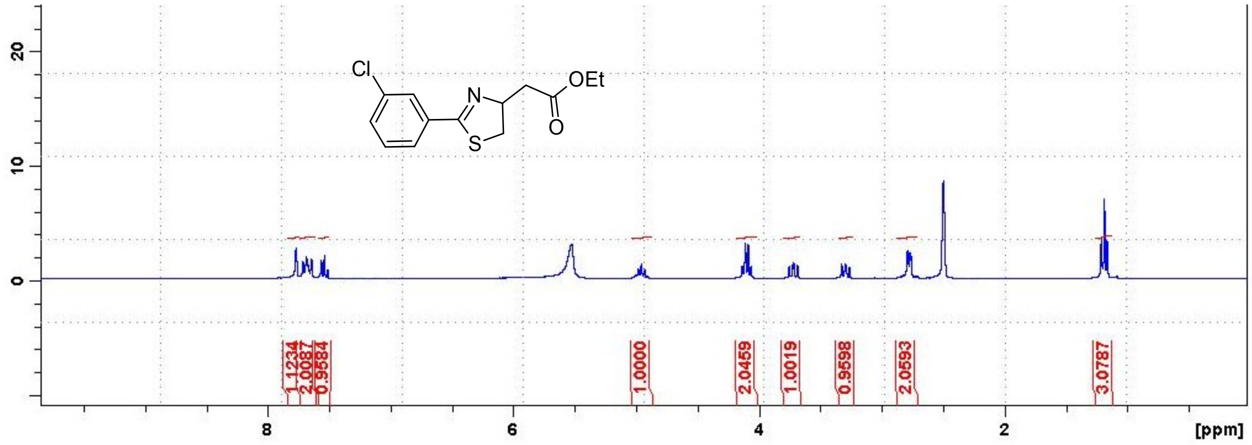

^{13}C NMR of **19**

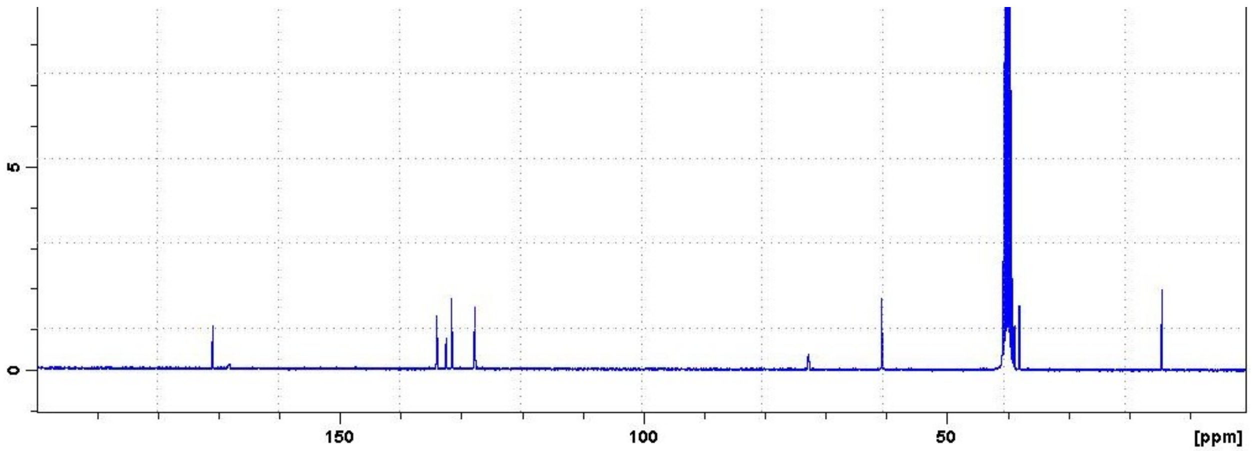

Ethyl [2-(4-chlorophenyl)-4,5-dihydro-1,3-thiazol-4-yl]acetate (20)

^{1}H NMR of **20**

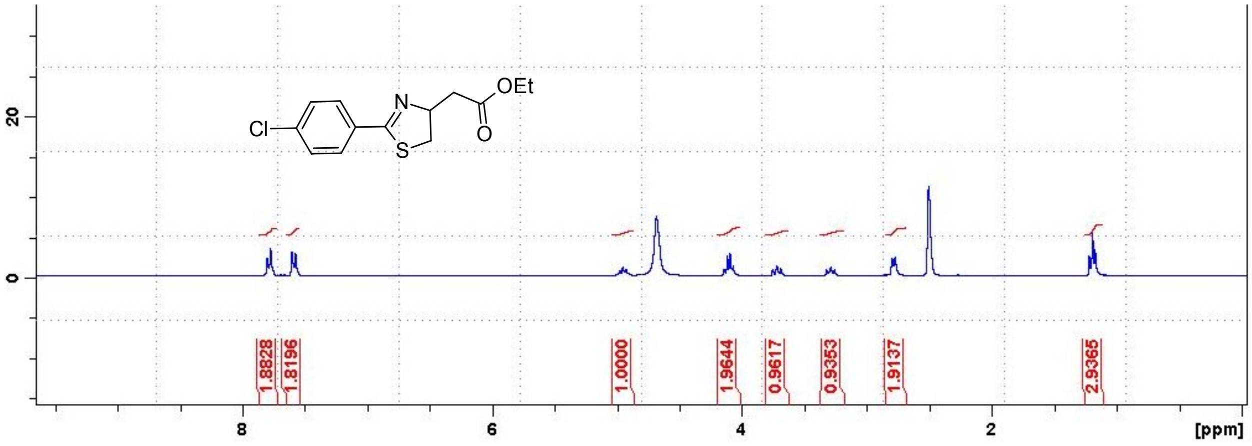

^{13}C NMR of **20**

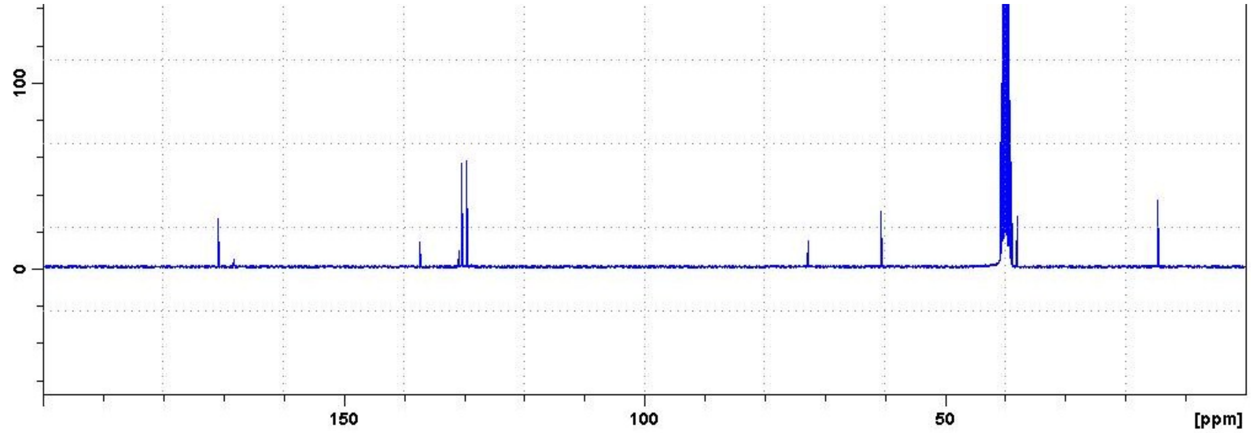

Ethyl {2-[4-(trifluoromethyl)phenyl]-4,5-dihydro-1,3-thiazol-4-yl}acetate (21)

^{1}H NMR of **21**

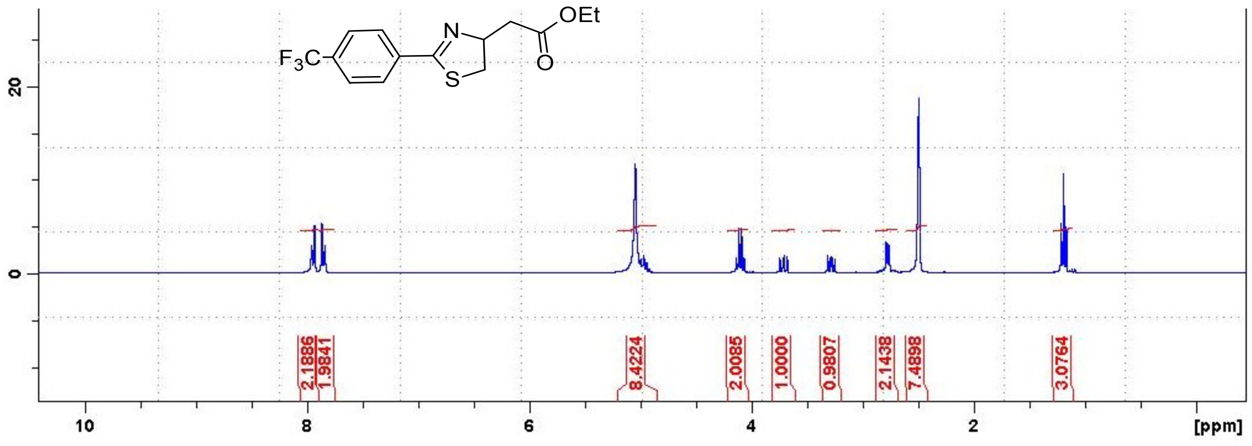

^{13}C NMR of **21**

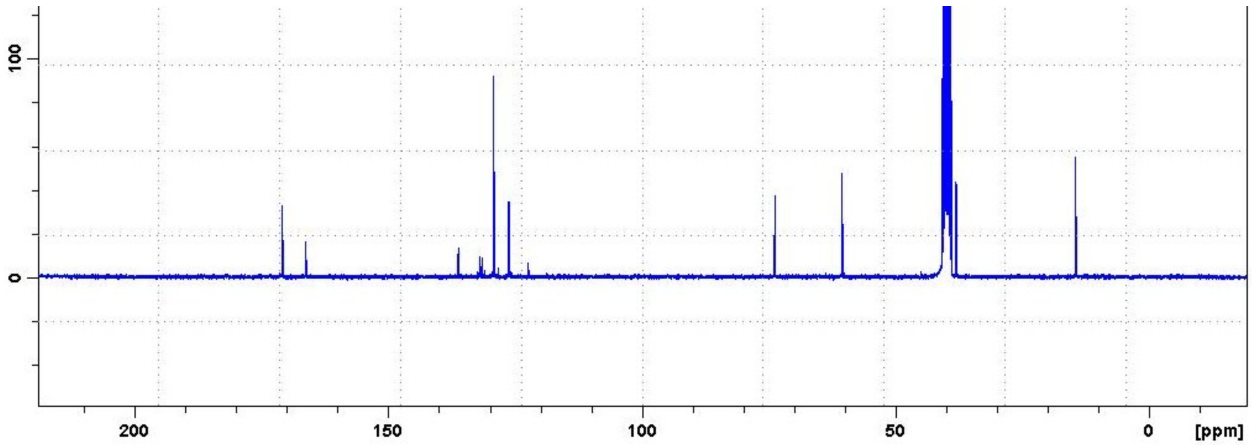

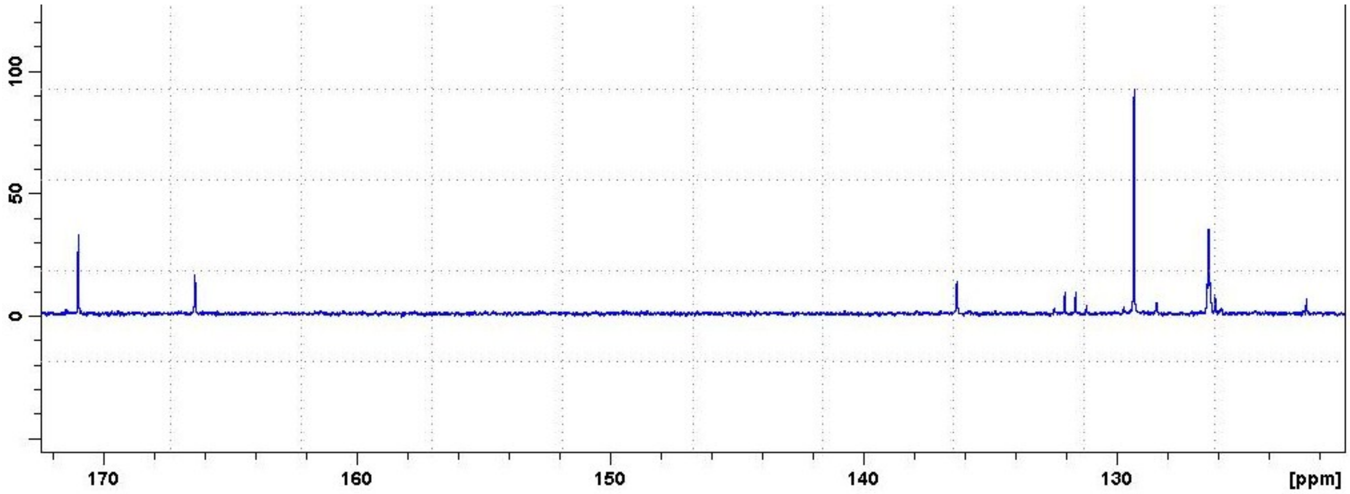

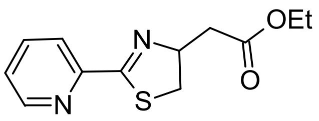

Ethyl [2-(XXXyridine-2-yl)-4,5-dihydro-1,3-thiazol-4-yl]acetate (22)

^{1}H NMR of **22**

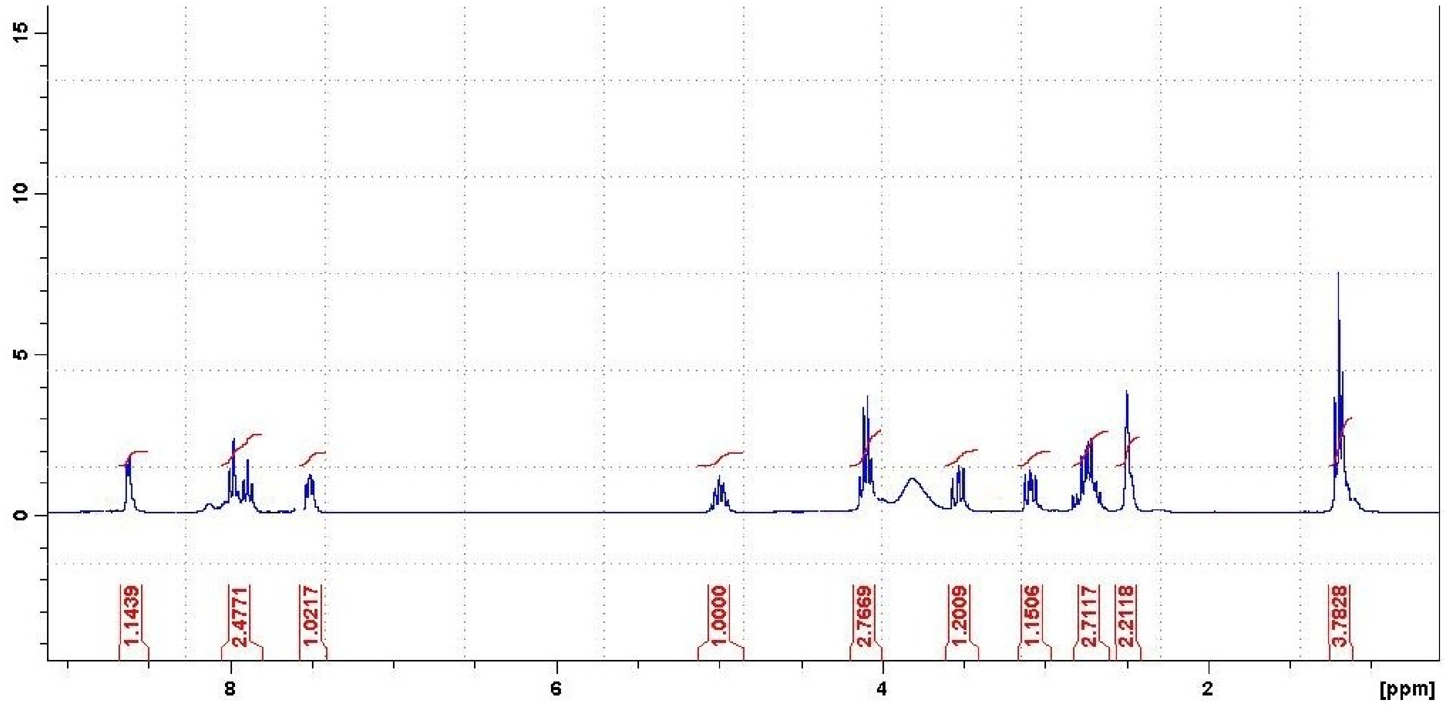

^{13}C NMR of **22**

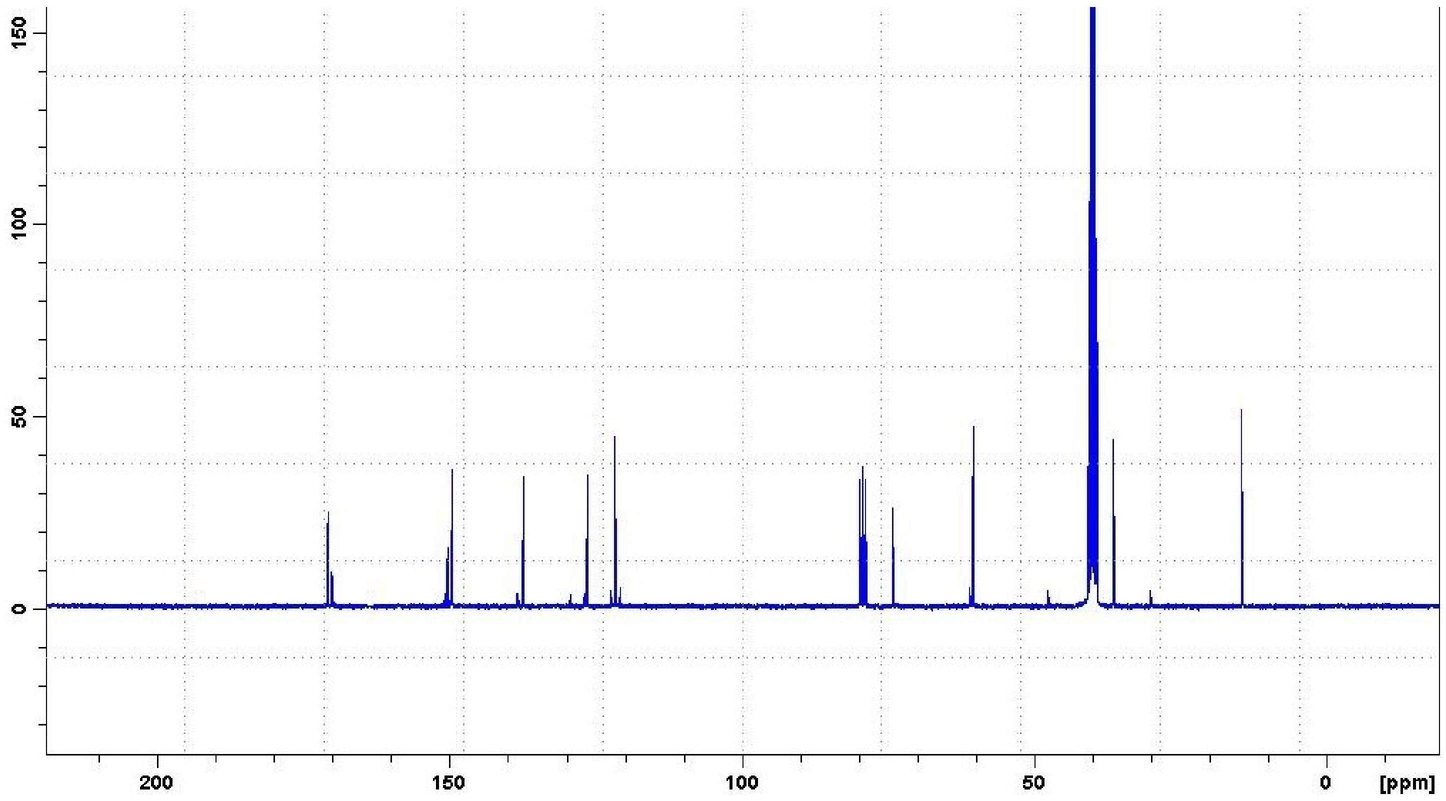

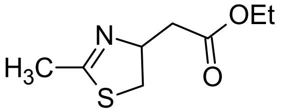

Ethyl (2-methyl-4,5-dihydro-1,3-thiazol-4-yl)acetate (23)

^{1}H NMR of **23**

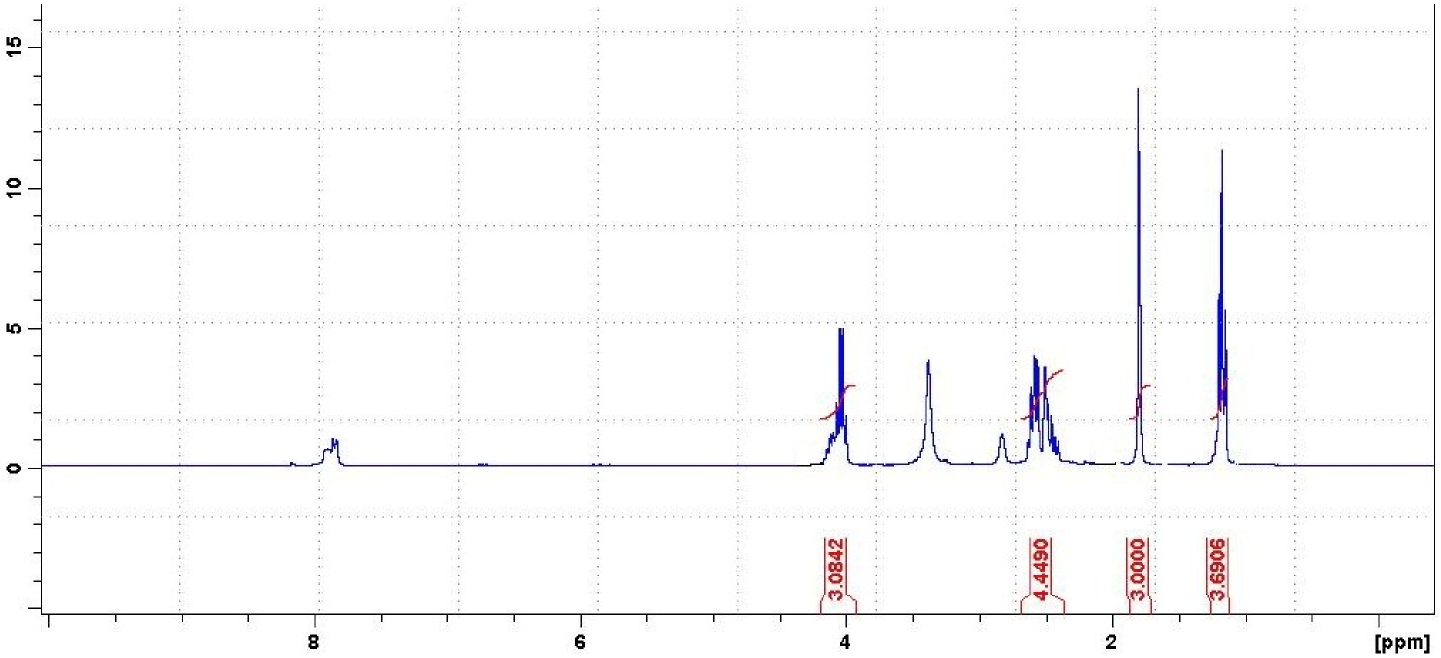

^{1}H NMR of **23**

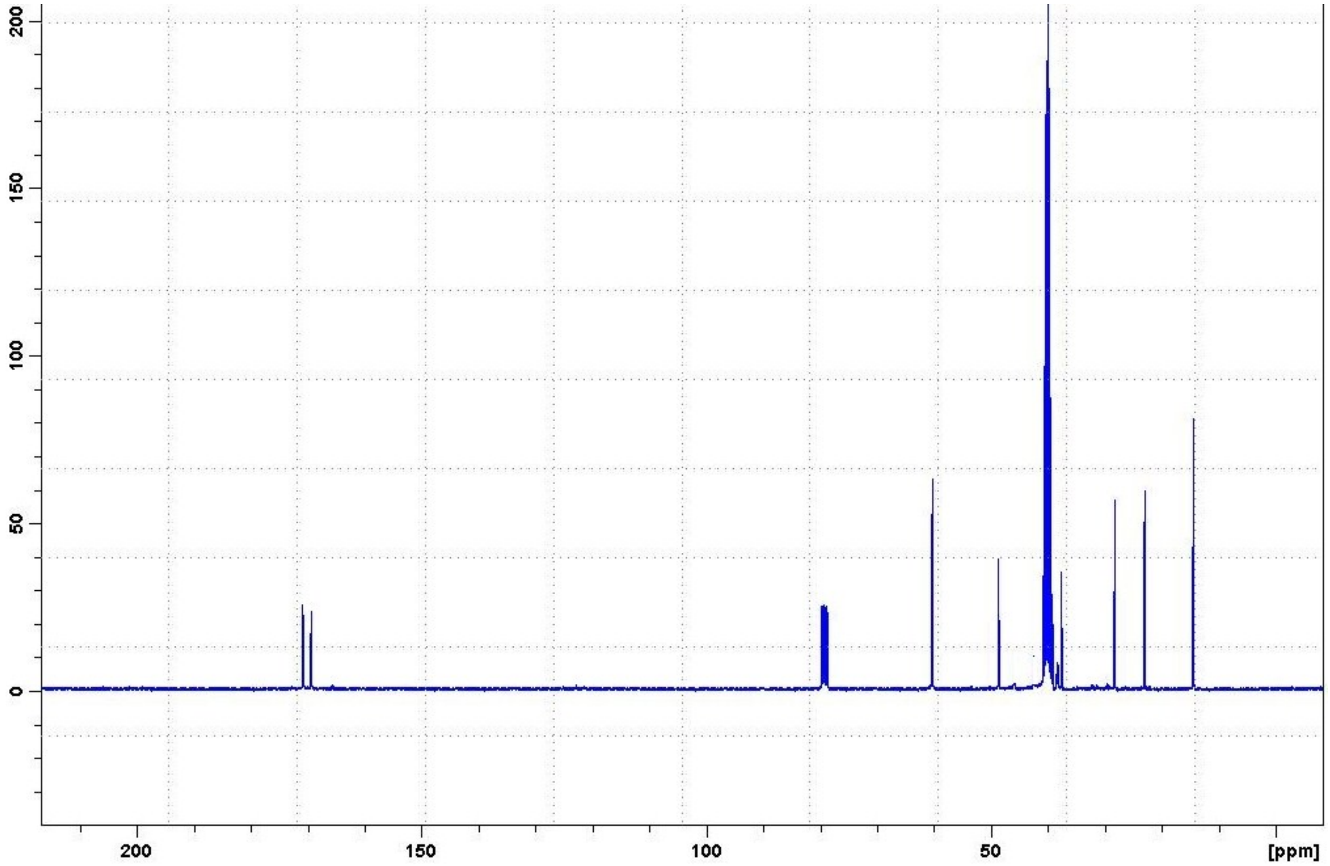

Ethyl {2-[(2*E*)-2-benzylidenehydrazinyl]-4,5-dihydro-1,3-thiazol-4-yl}acetate (24)

^{1}H NMR of **24**

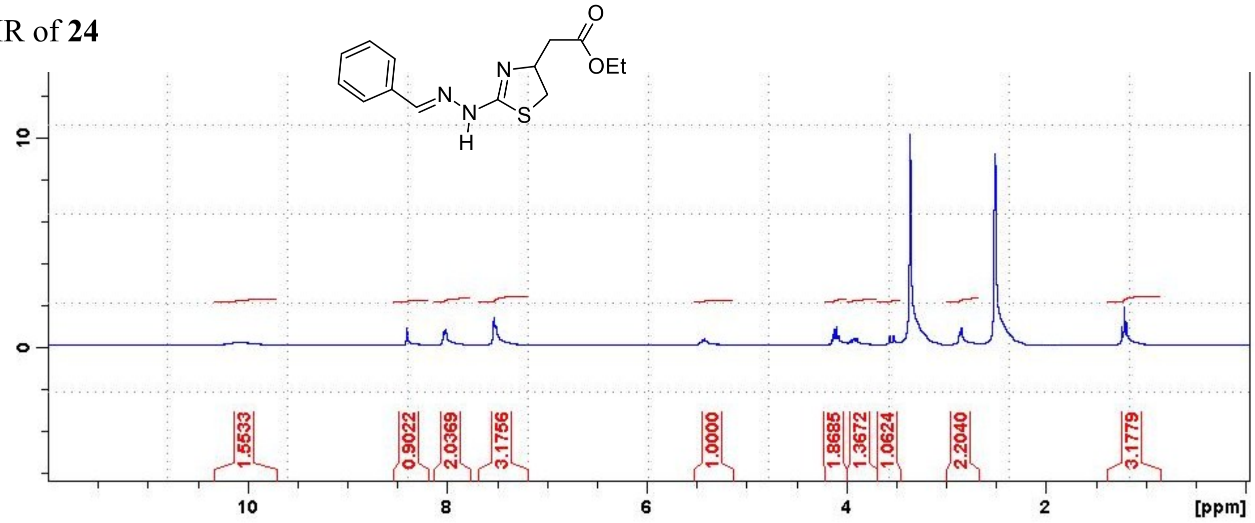

^{13}C NMR of **24**

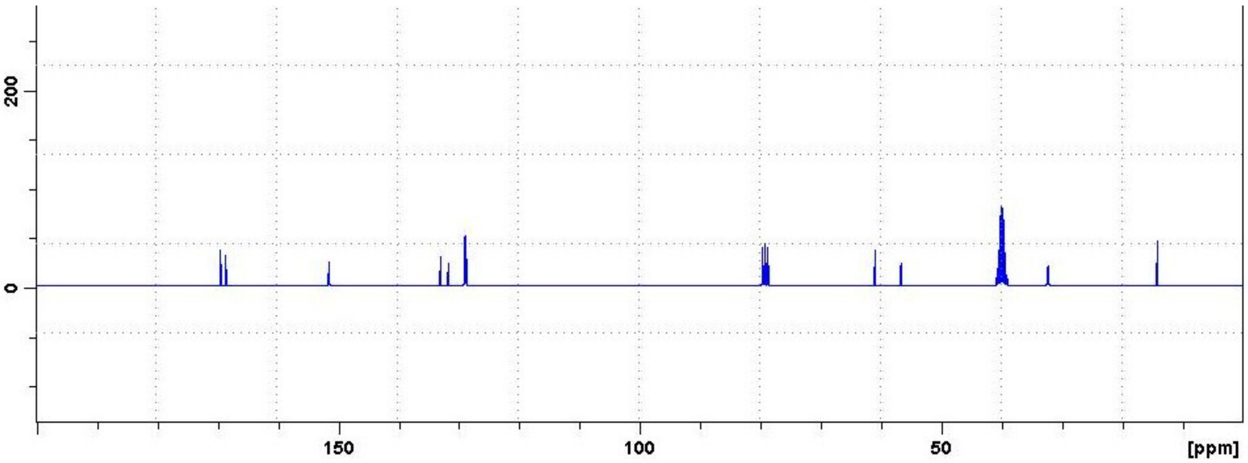

Ethyl (2-{(2*E*)-2-[(4-hydroxyphenyl)methylidene]hydrazinyl}-4,5-dihydro-1,3-thiazol-4-yl)acetate (25)

^{1}H NMR of **25**

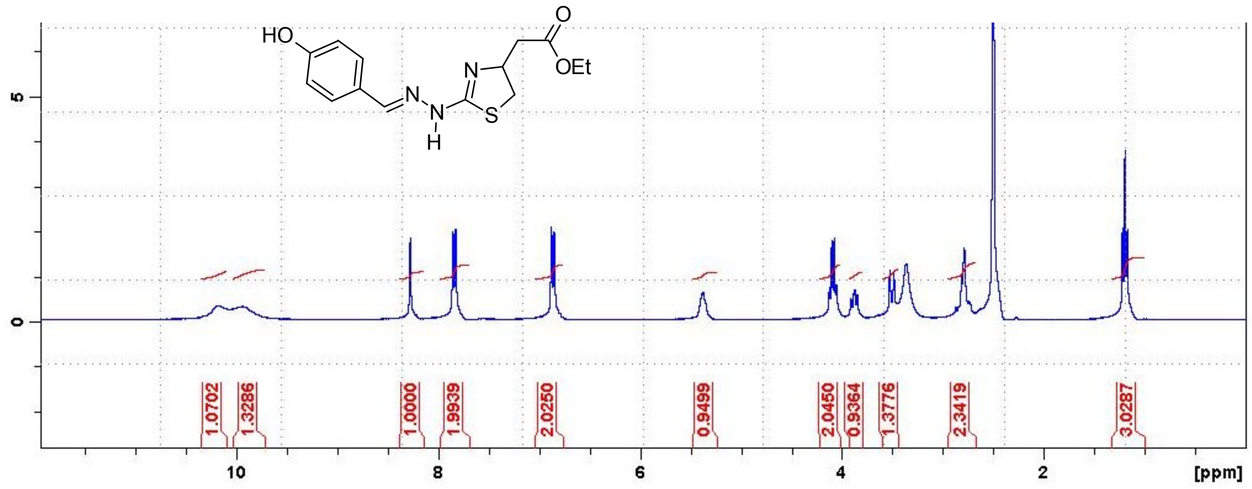

^{13}C NMR of **25**

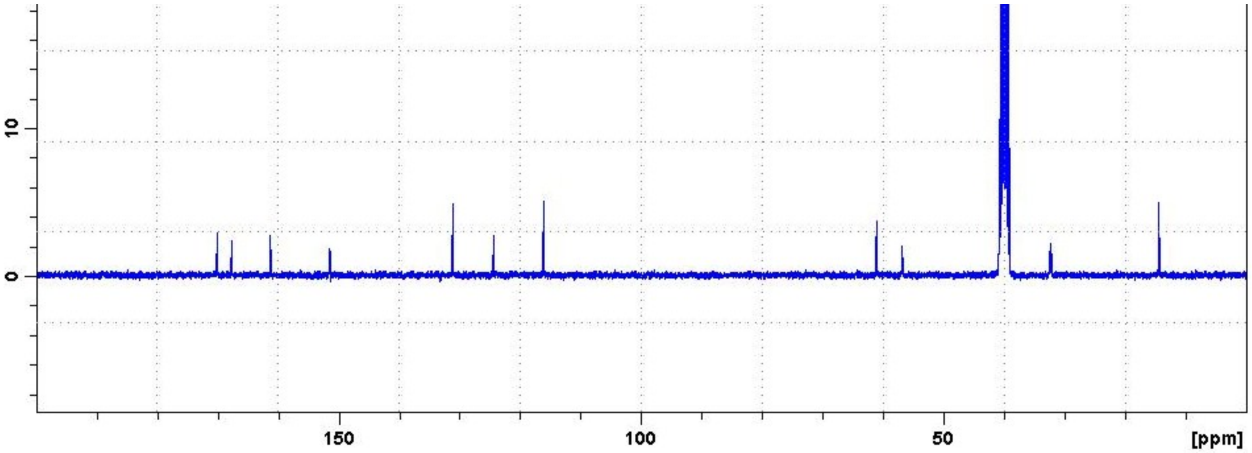

Ethyl {2-[(2*E*)-2-{[4-(1*H*-pyrazol-1-yl)phenyl]methylidene}hydrazinyl]-4,5-dihydro-1,3-thiazol-4-yl}acetate (26)

^{1}H NMR of **26**

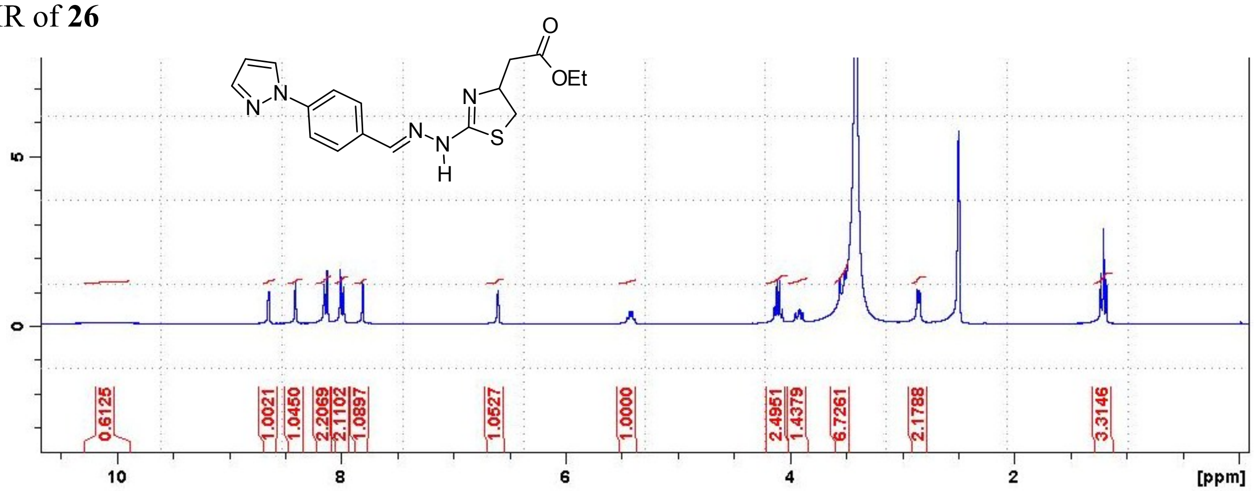

^{13}C NMR of **26**

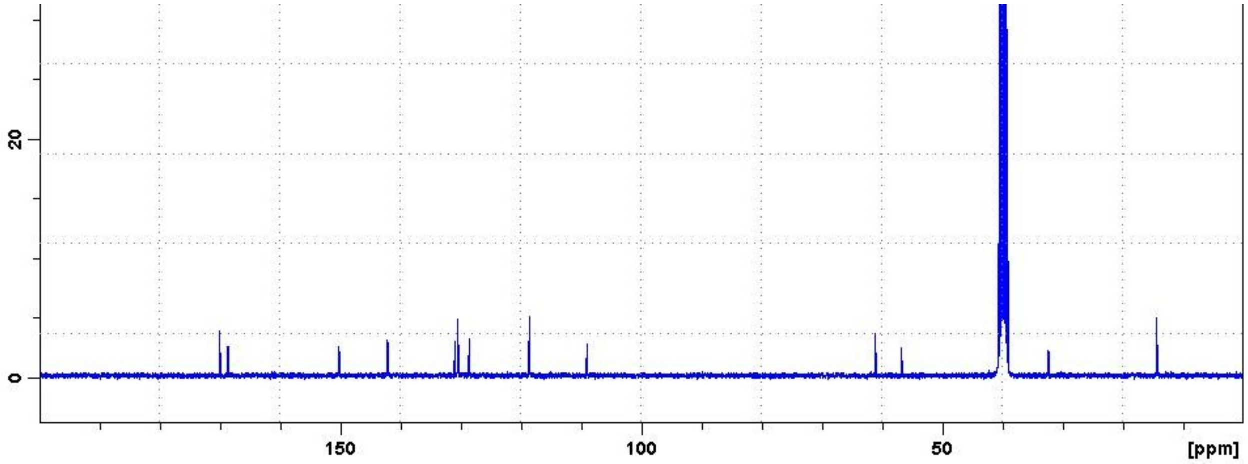

Ethyl {2-[(2*E*)-2-{[4-(dimethylamino)phenyl]methylidene}hydrazinyl]-4,5-dihydro-1,3-thiazol-4-yl}acetate (27)

^{1}H NMR of **27**

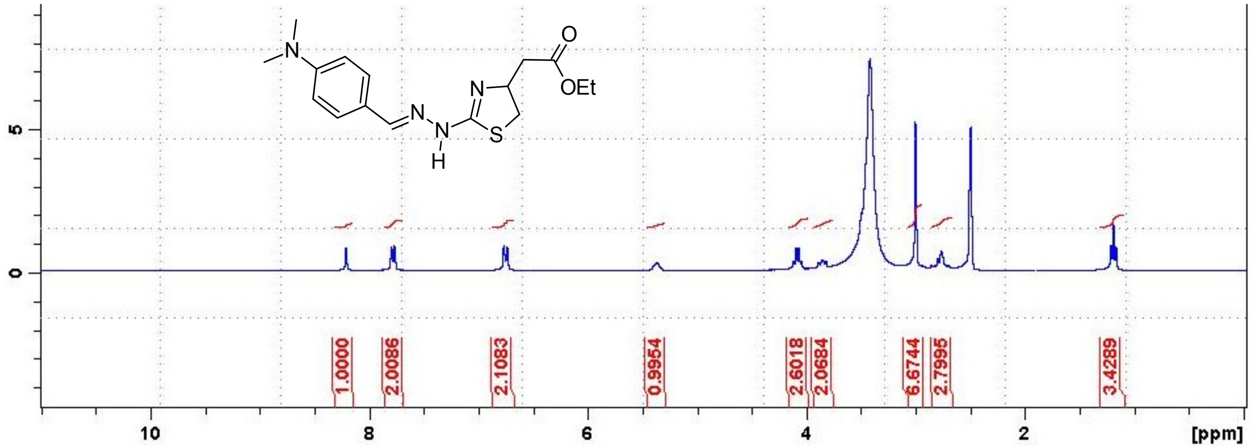

^{13}C NMR of **27**

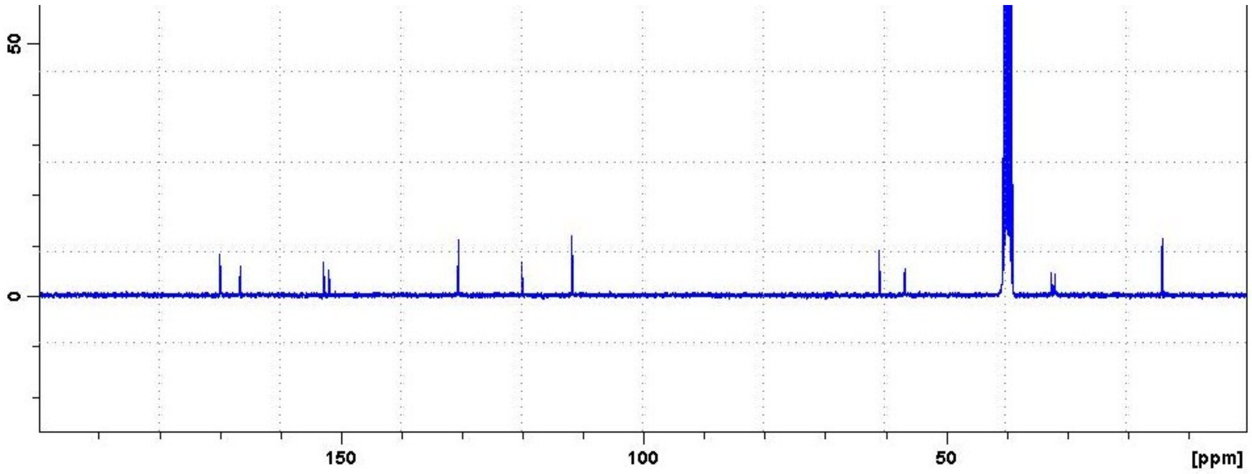

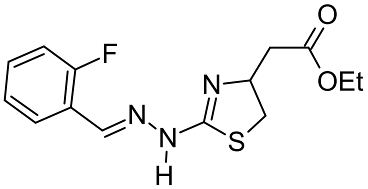

Ethyl (2-{(2*E*)-2-[(2-fluorophenyl)methylidene]hydrazinyl}-4,5-dihydro-1,3-thiazol-4-yl)acetate (28)

^{1}H NMR of **28**

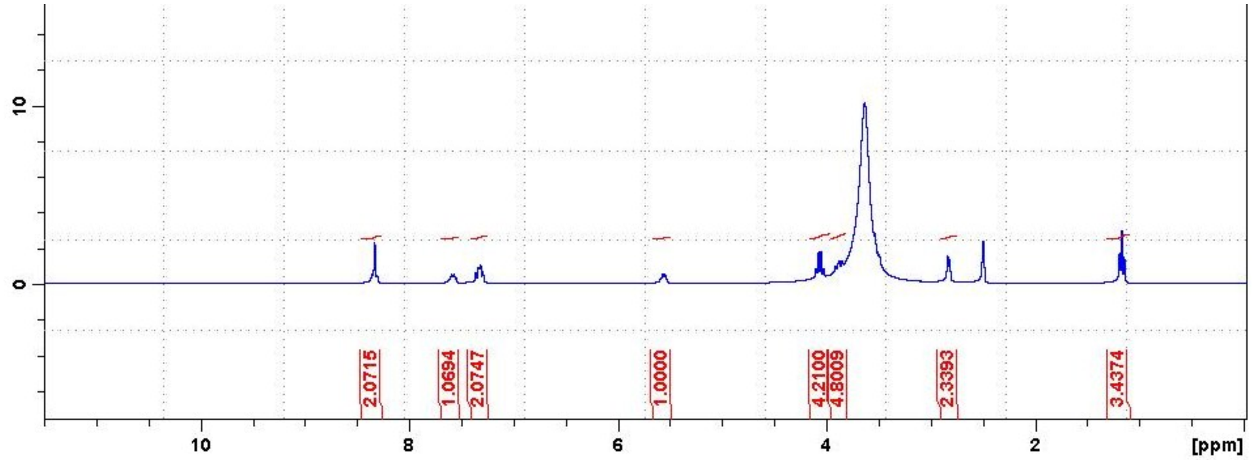

^{13}C NMR of **28**

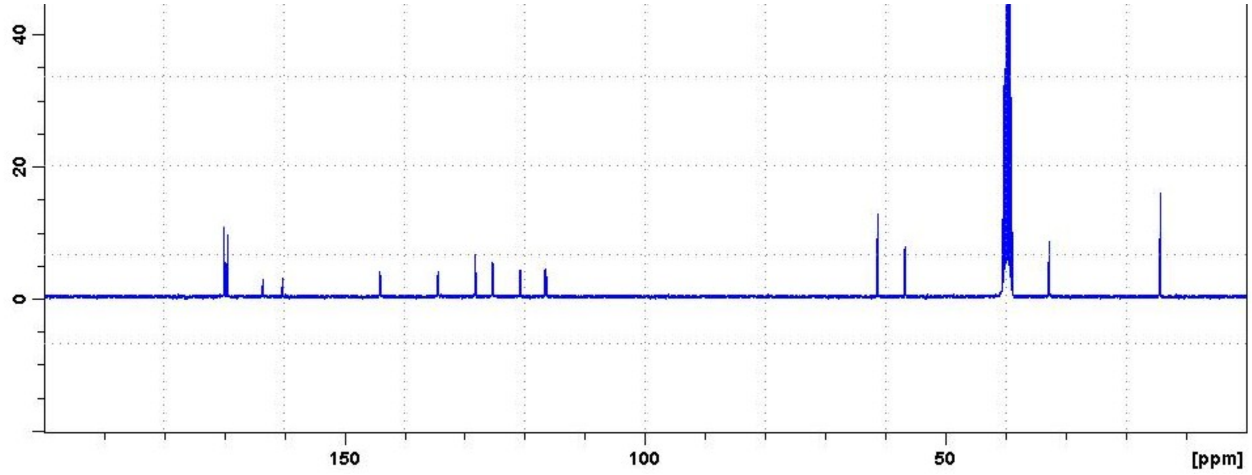

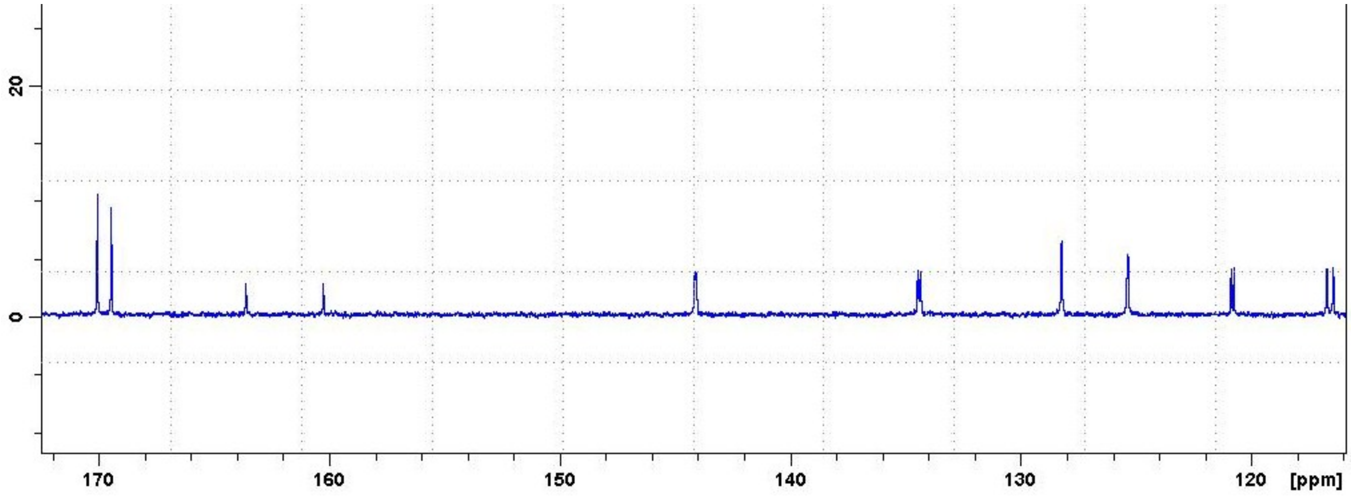

Ethyl (2-{(2*E*)-2-[(4-fluorophenyl)methylidene]hydrazinyl}-4,5-dihydro-1,3-thiazol-4-yl)acetate (29)

^{1}H NMR of **29**

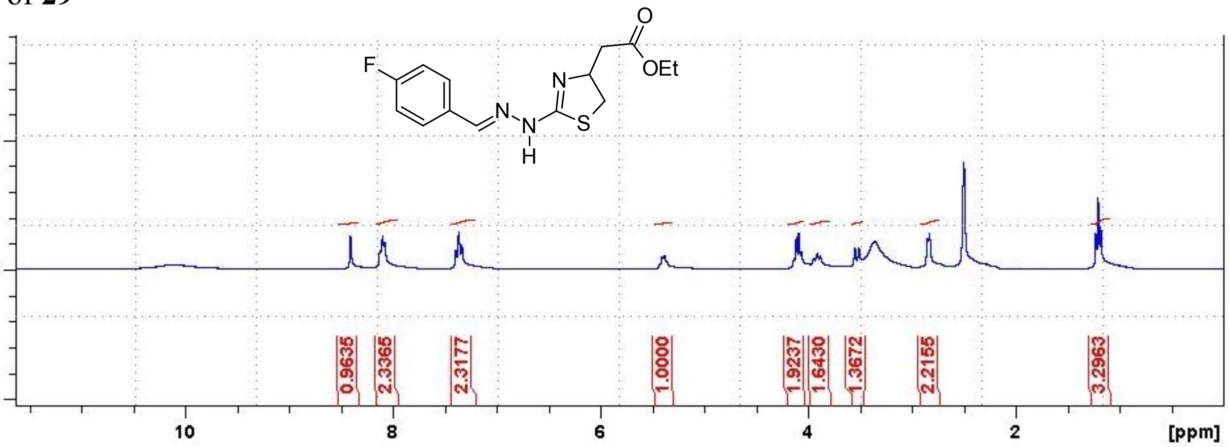

^{13}C NMR of **29**

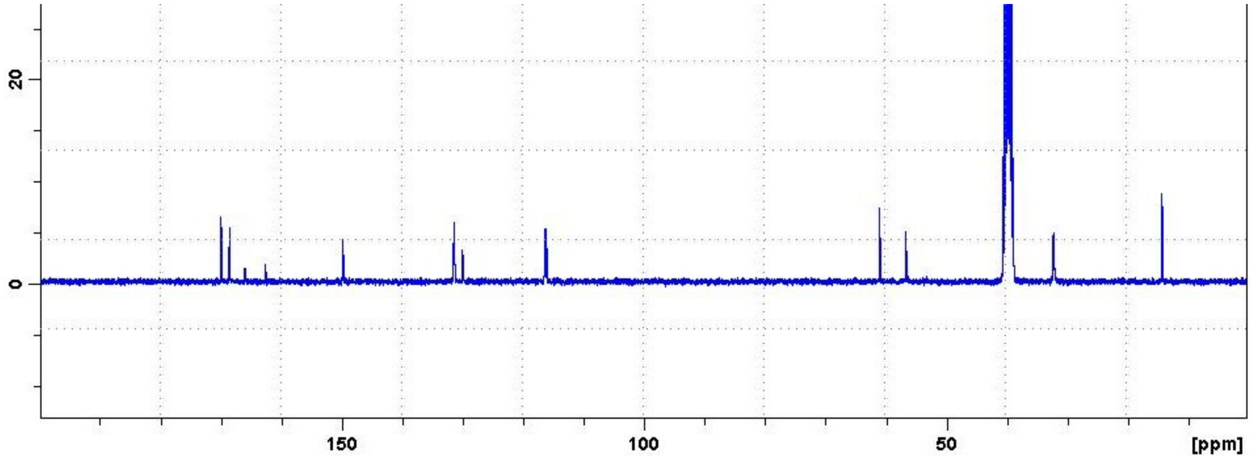

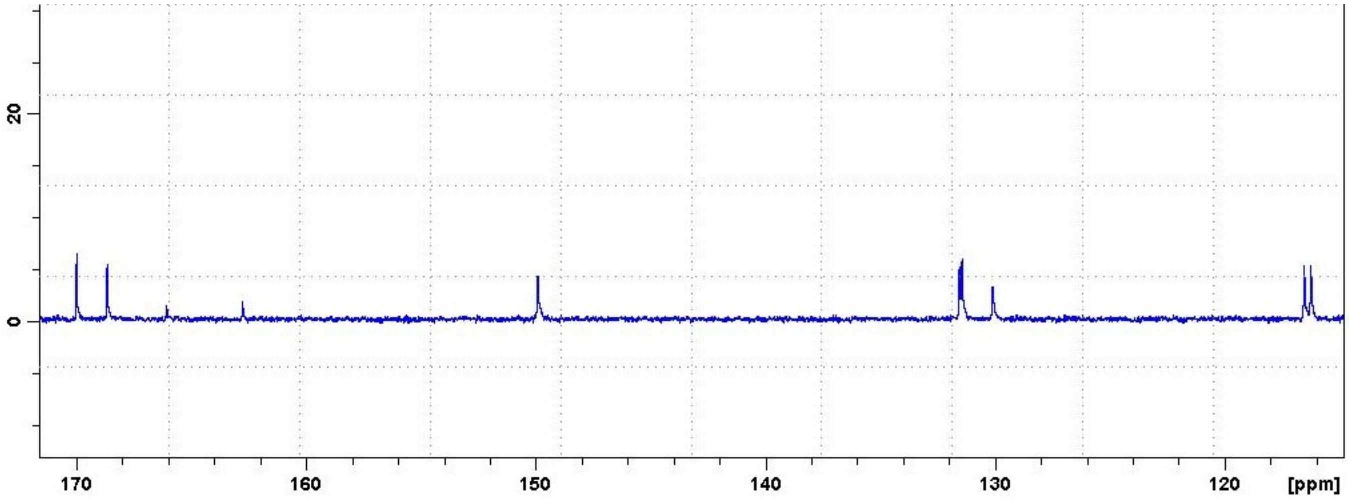

Ethyl (2-{(2*E*)-2-[(4-chlorophenyl)methylidene]hydrazinyl}-4,5-dihydro-1,3-thiazol-4-yl)acetate (30)

^{1}H NMR of **30**

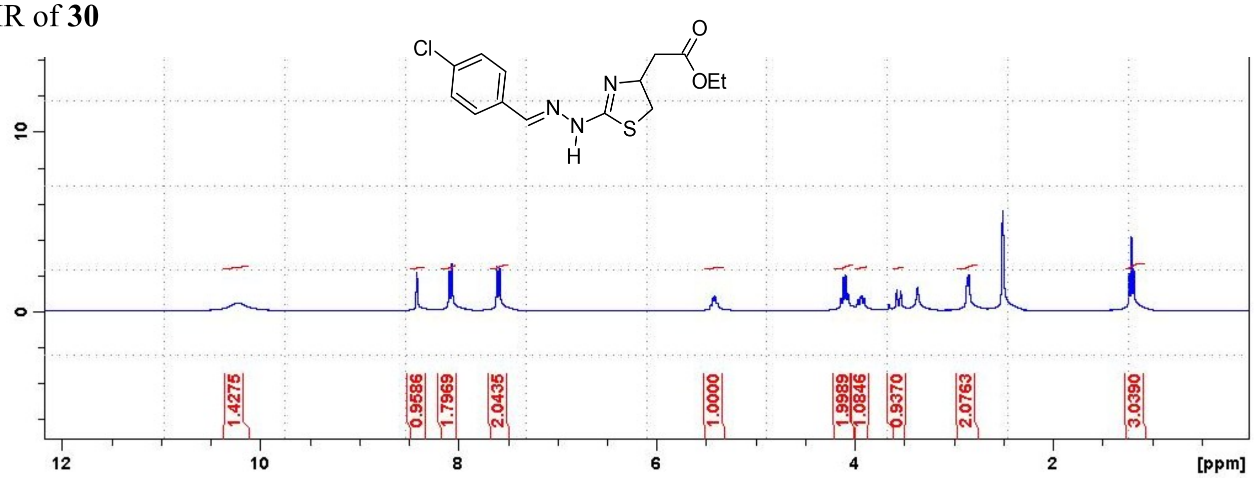

^{13}C NMR of **30**

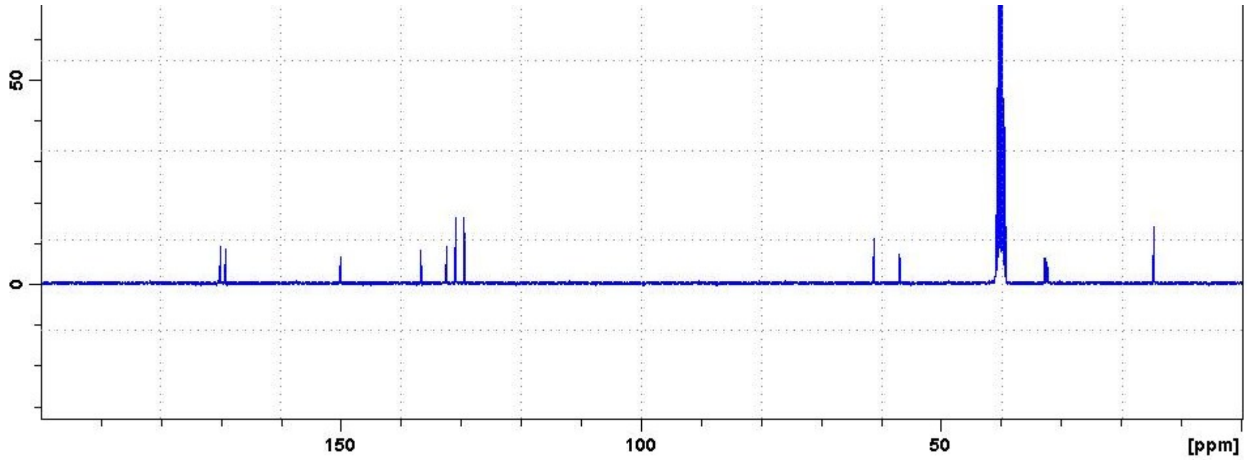

Ethyl (2-{(2*E*)-2-[(3,4-difluorophenyl)methylidene]hydrazinyl}-4,5-dihydro-1,3-thiazol-4-yl)acetate (31)

^{1}H NMR of **31**

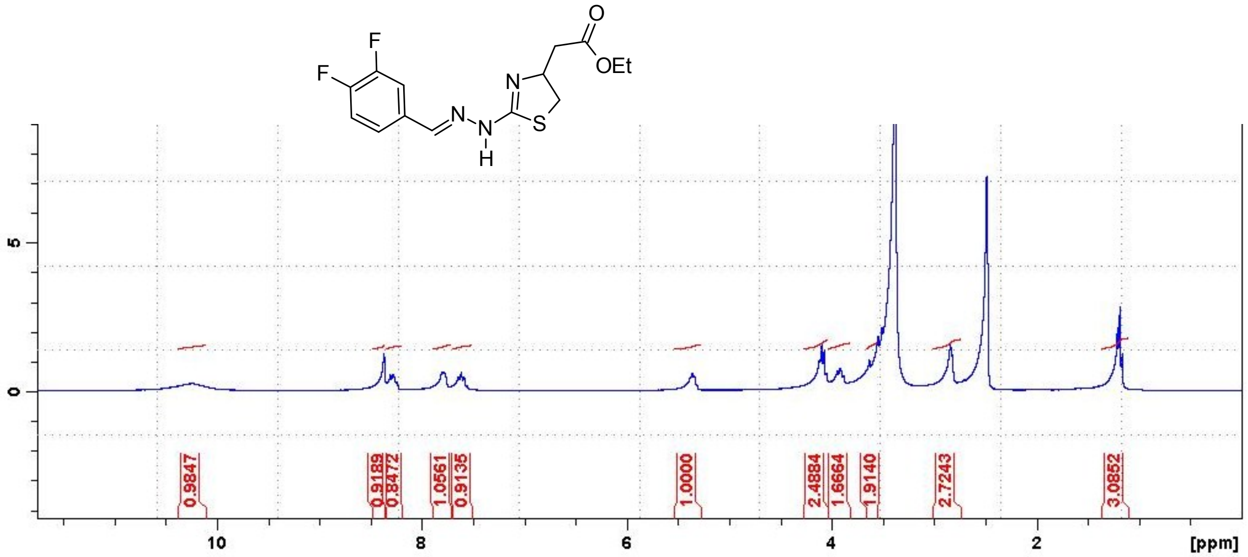

^{13}C NMR of **31**

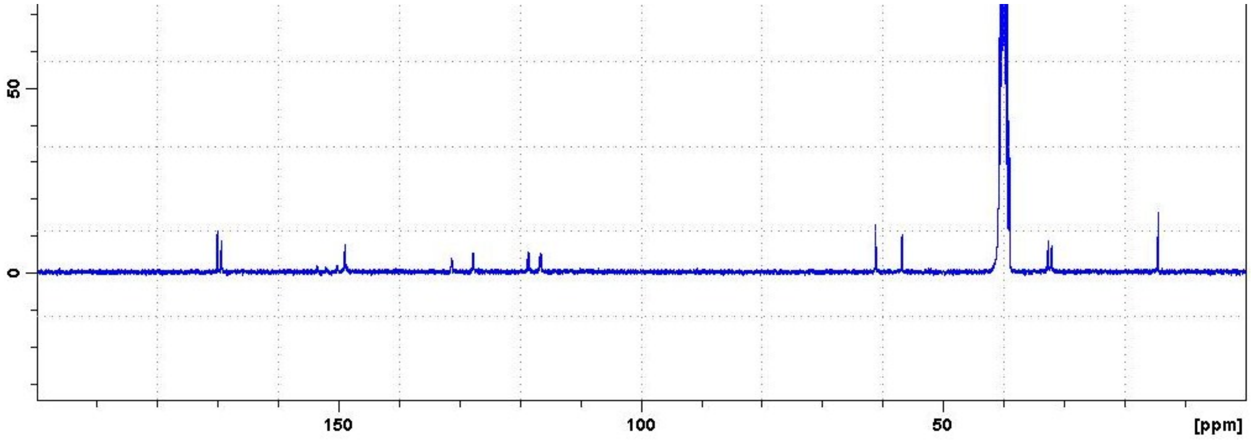

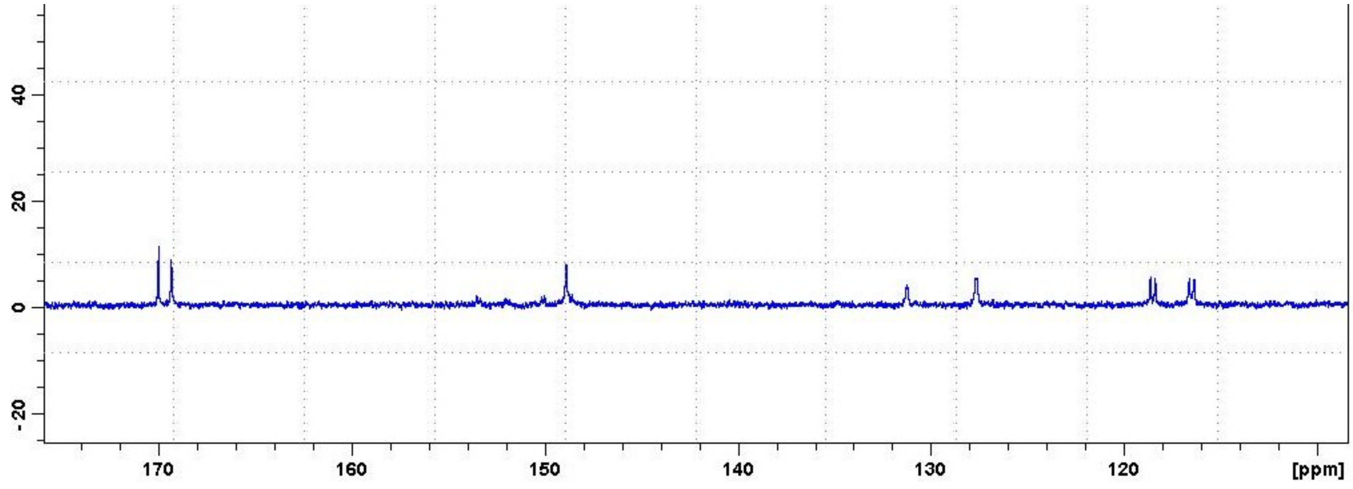

Ethyl (2-{(2*E*)-2-[(3-bromo-4-fluorophenyl)methylidene]hydrazinyl}-4,5-dihydro-1,3-thiazol-4-yl)acetate (32)

^{1}H NMR of **32**

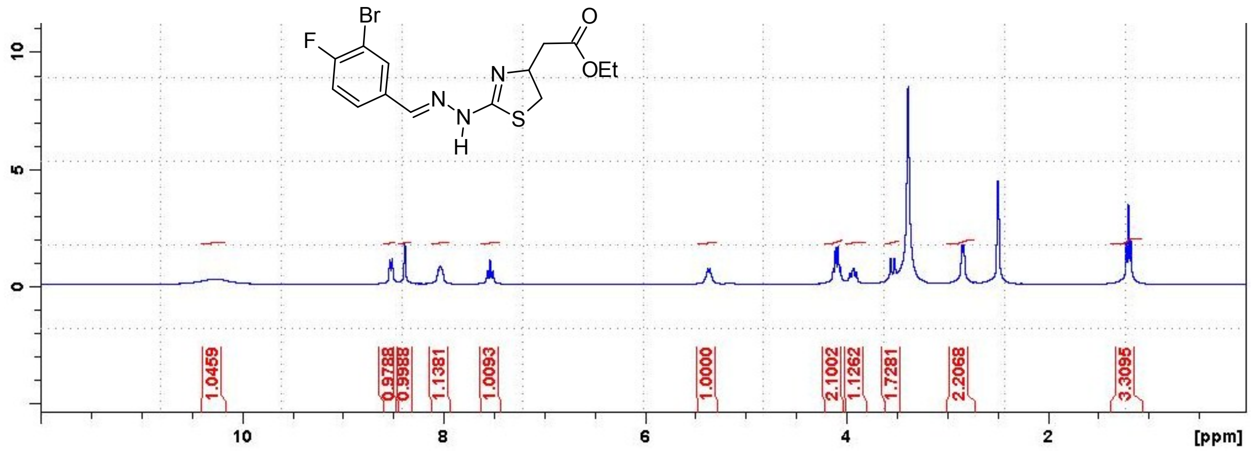

^{13}C NMR of **32**

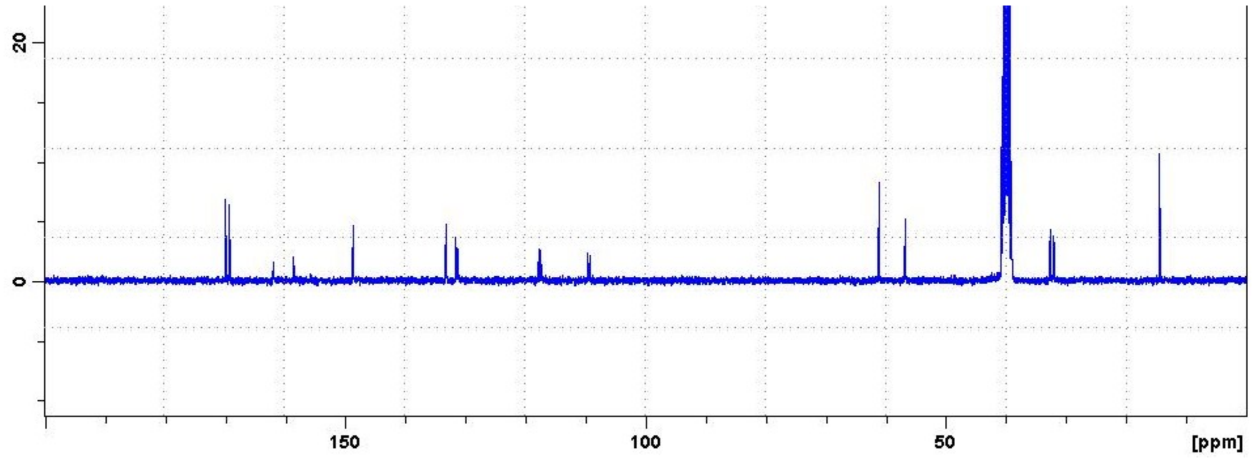

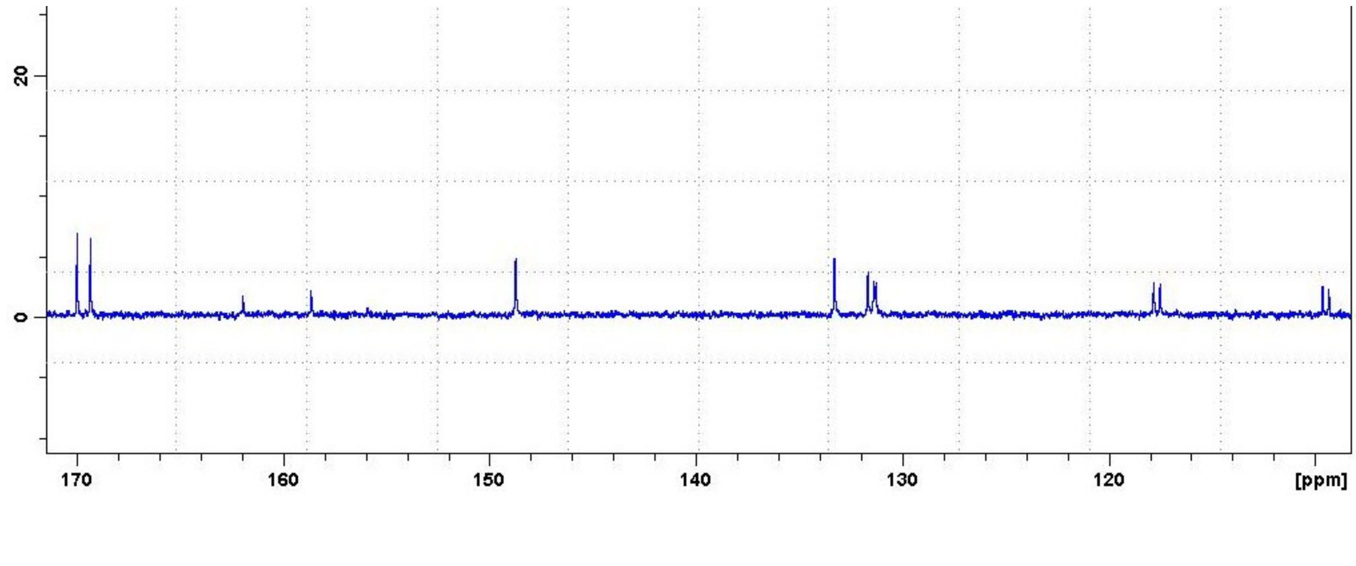

Ethyl (2-{(2*E*)-2-[(3-nitrophenyl)methylidene]hydrazinyl}-4,5-dihydro-1,3-thiazol-4-yl)acetate (33)

^{1}H NMR of **33**

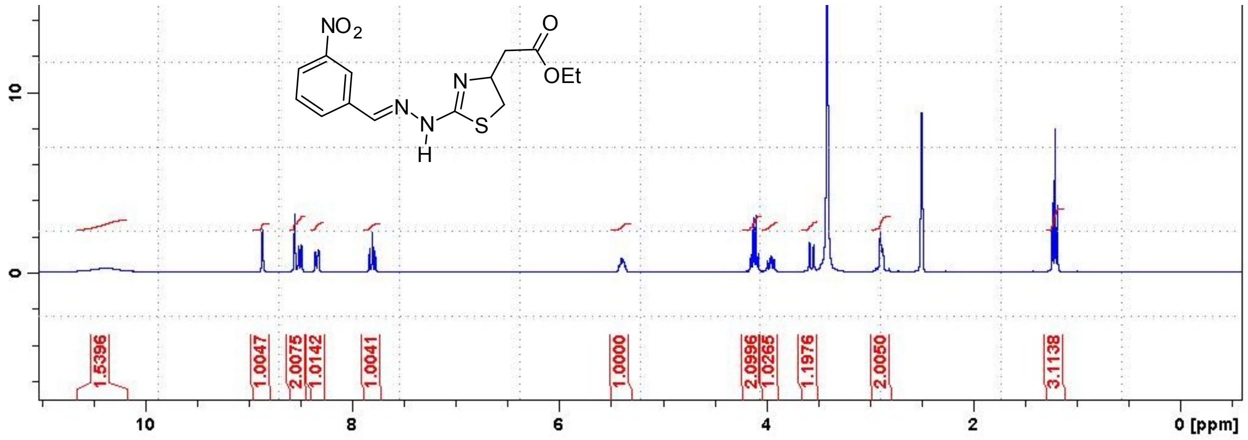

^{13}C NMR of **33**

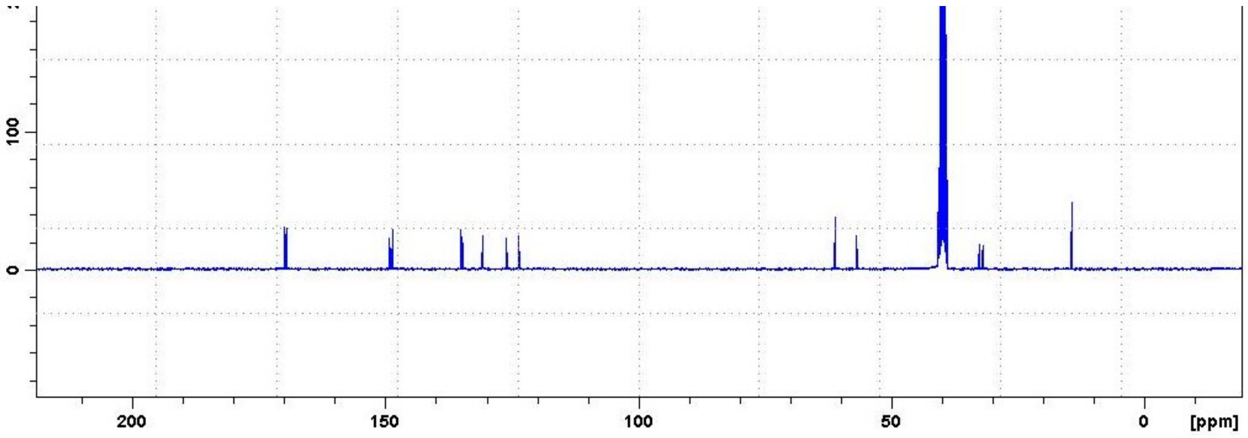

Ethyl (2-{(2*E*)-2-[(4-nitrophenyl)methylidene]hydrazinyl}-4,5-dihydro-1,3-thiazol-4-yl)acetate (34)

^{1}H NMR of **34**

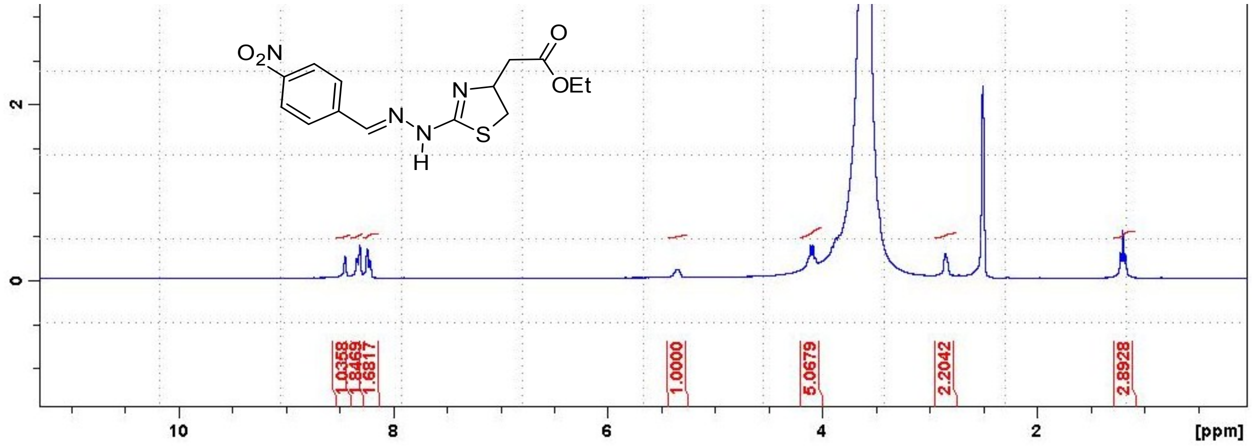

^{13}C NMR of **34**

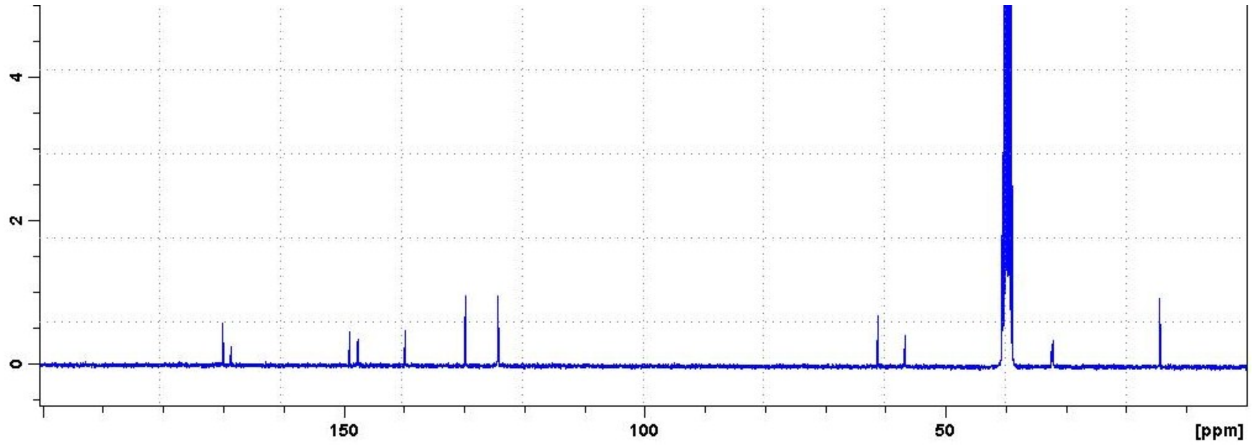

Ethyl (2-{(2*E*)-2-[(3*H*-indol-3-yl)methylidene]hydrazinyl}-4,5-dihydro-1,3-thiazol-4-yl)acetate (35)

^{1}H NMR of **35**

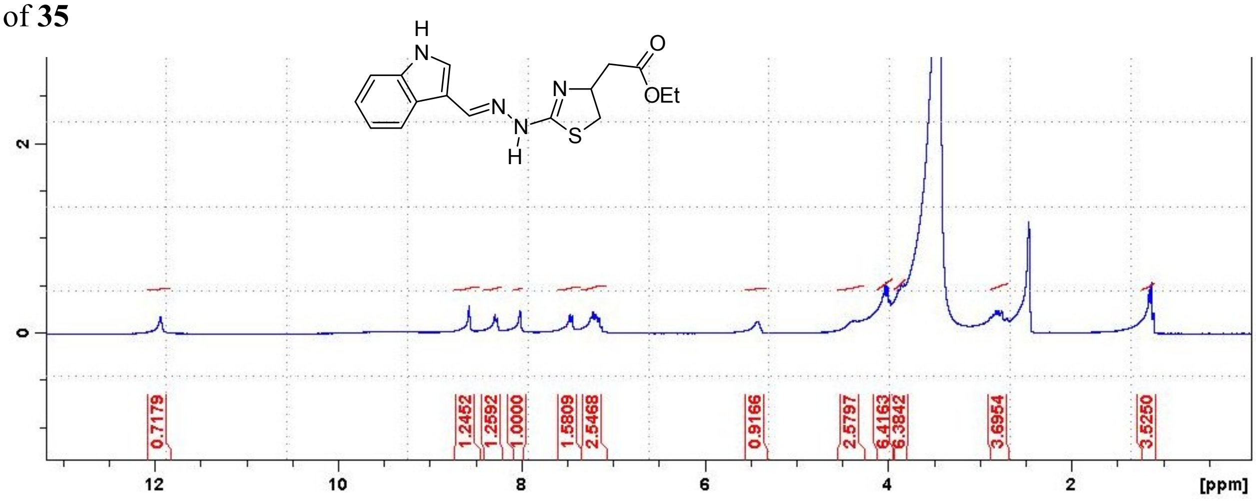

^{13}C NMR of **35**

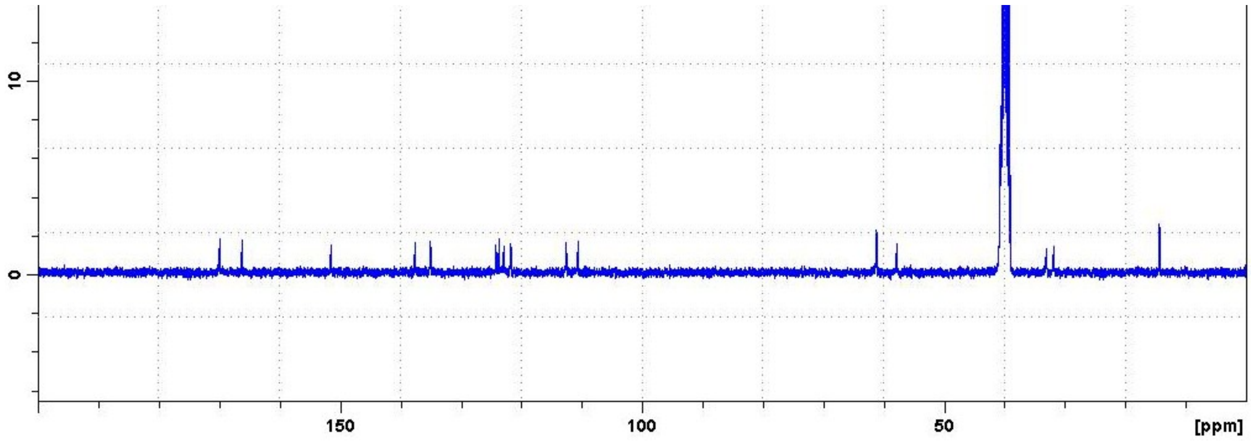

Ethyl {2-[(2*Z*)-2-(1-phenylethylidene)hydrazinyl]-4,5-dihydro-1,3-thiazol-4-yl}acetate (36)

^{1}H NMR of **36**

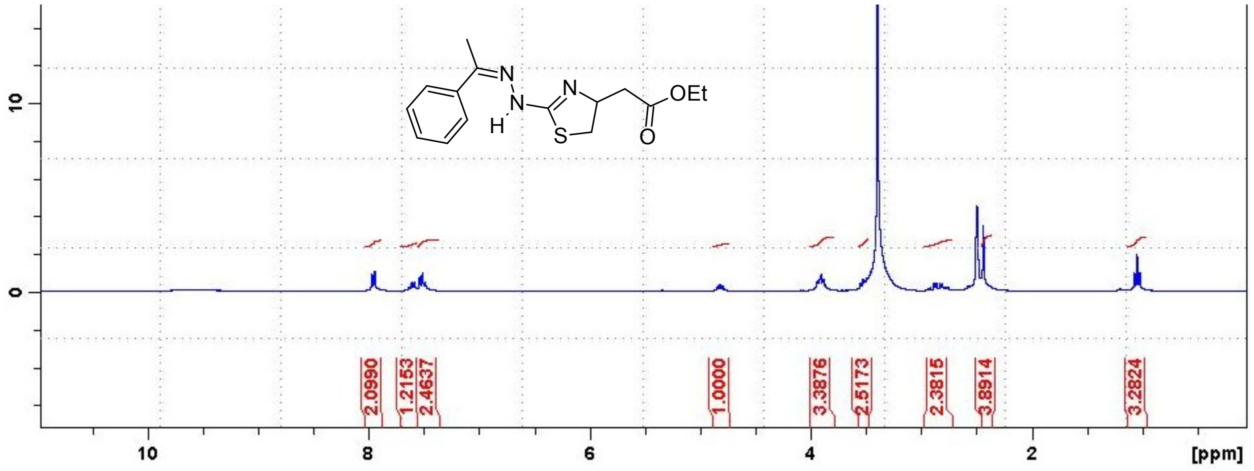

^{13}C NMR of **36**

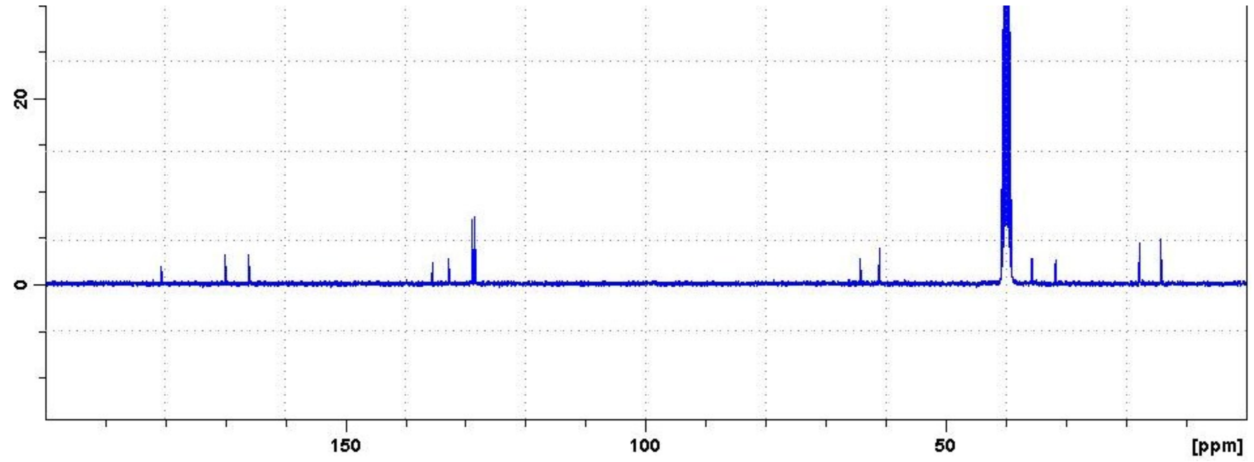

Ethyl {2-[(2*E*)-2-{2-[3-(trifluoromethyl)phenyl]propylidene}hydrazinyl]-4,5-dihydro-1,3-thiazol-4-yl}acetate (37)

^{1}H NMR of **37**

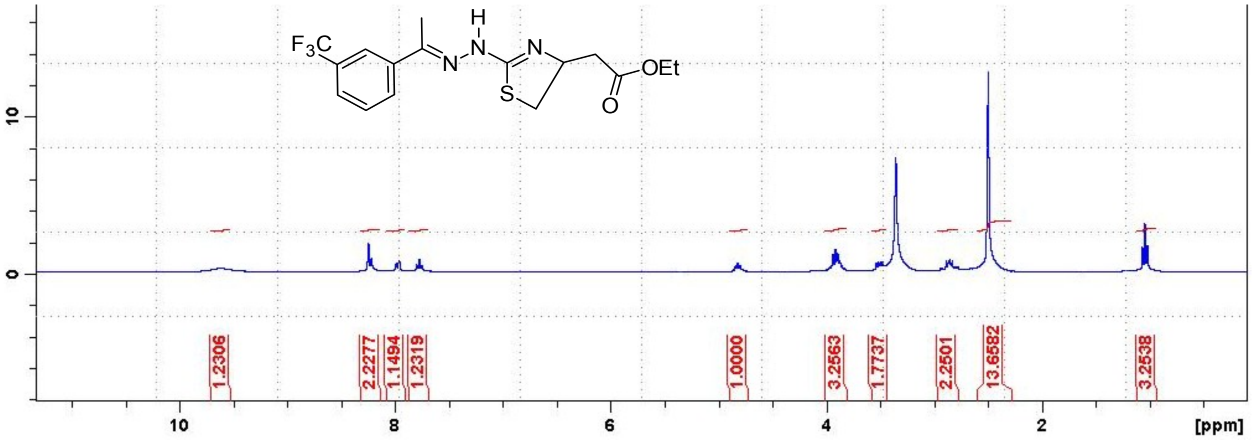

^{13}C NMR of **37**

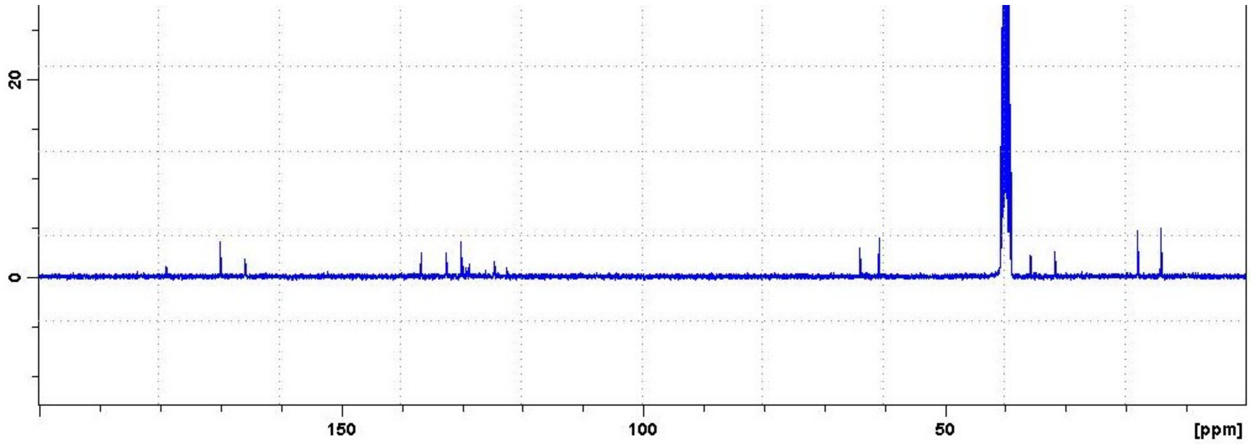

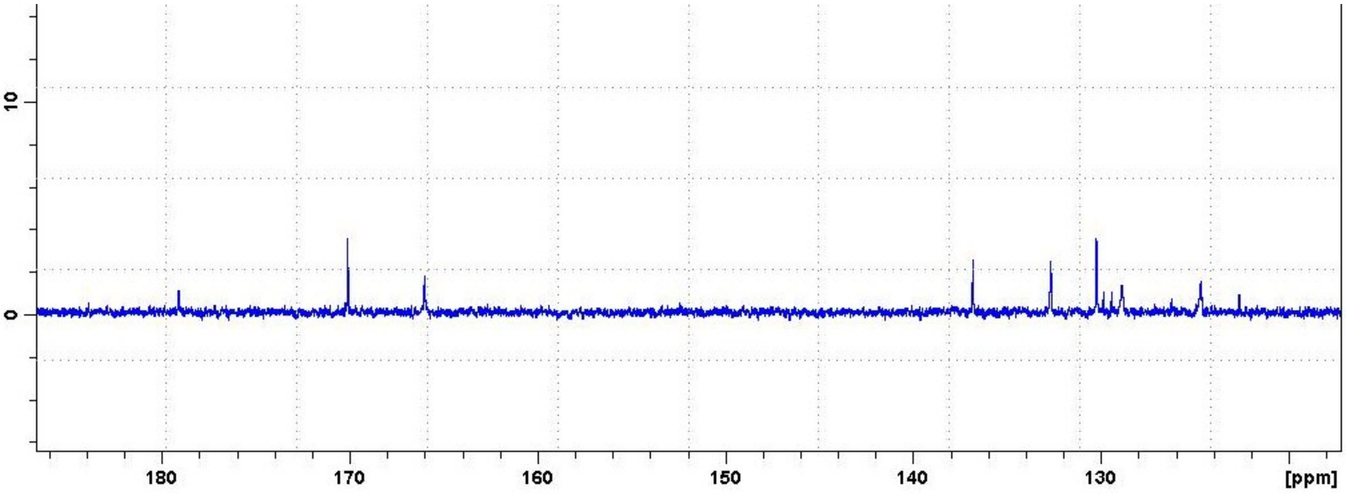

Ethyl (2-{(2*E*)-2-[2-(naphthalen-2-yl)propylidene]hydrazinyl}-4,5-dihydro-1,3-thiazol-4-yl)acetate (38)

^{1}H NMR of **38**

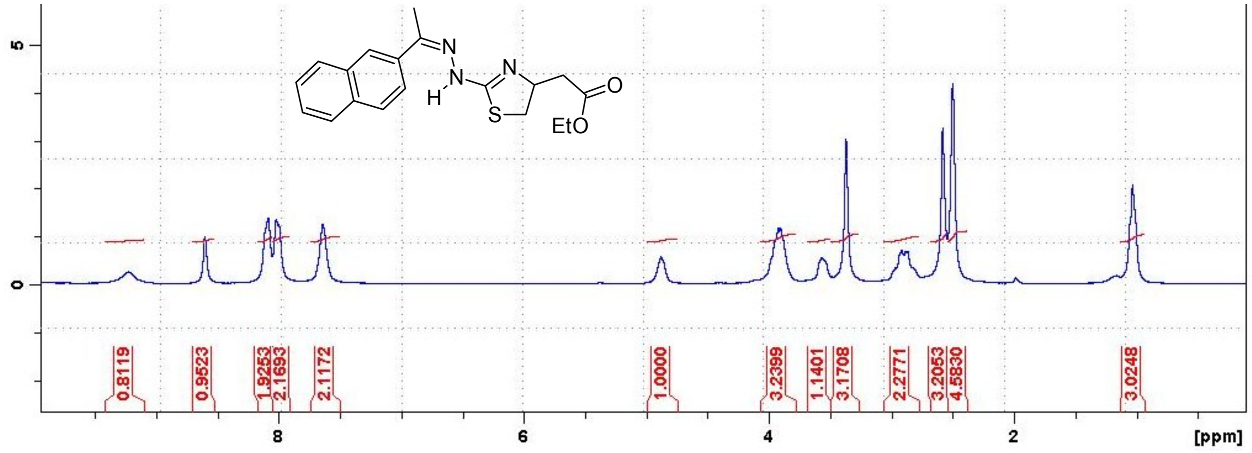

^{13}C NMR of **38**

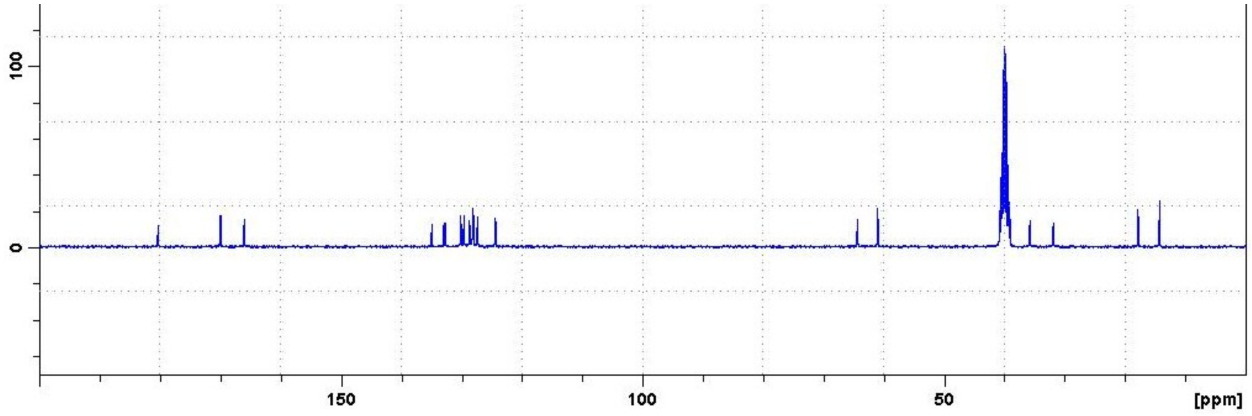

Ethyl (2-{(2*Z*)-2-[1-(6-methoxynaphthalen-2-yl)ethylidene]hydrazinyl}-4,5-dihydro-1,3-thiazol-4-yl)acetate (39)

^{1}H NMR of **39**

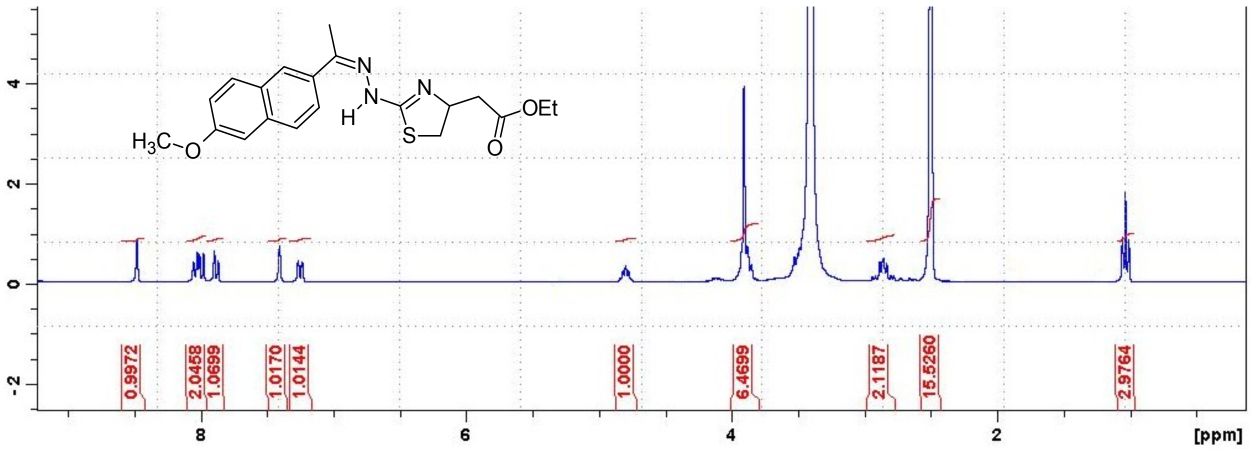

^{13}C NMR of **39**

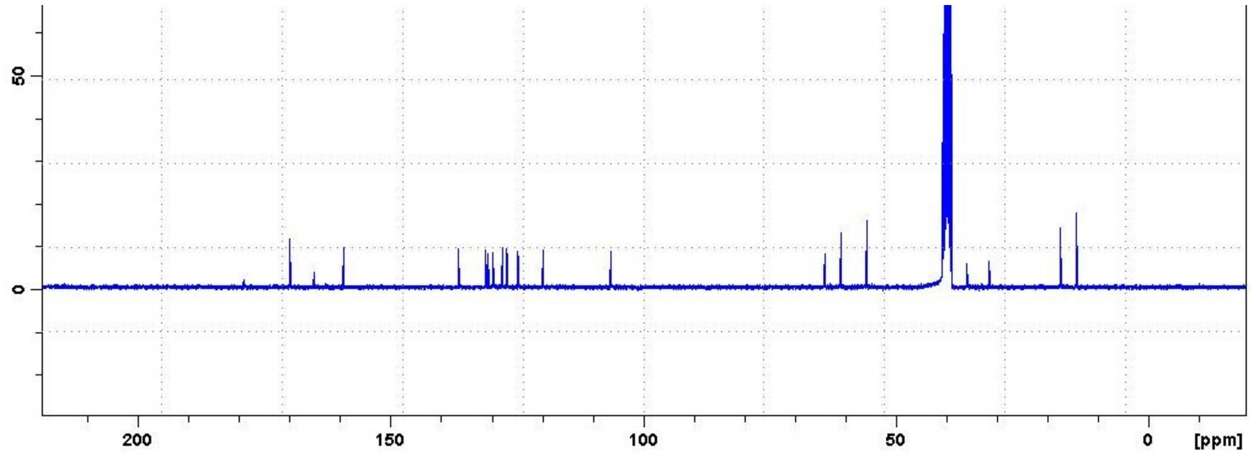

4-{[4-(2-Ethoxy-2-oxoethyl)-1,3-thiazol-2-yl]amino}benzoic acid (40)

^{1}H NMR of **40**

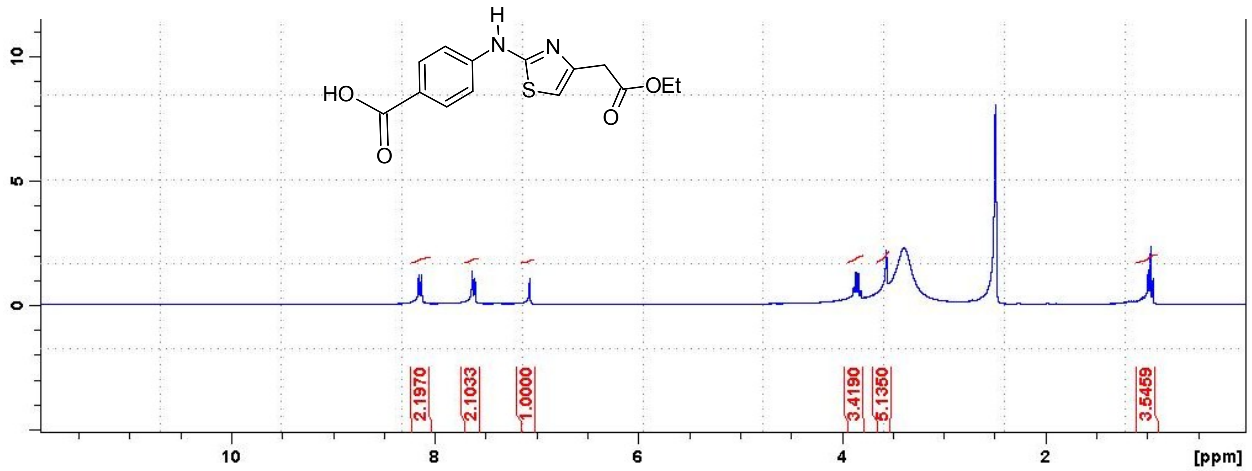

^{13}C NMR of **40**

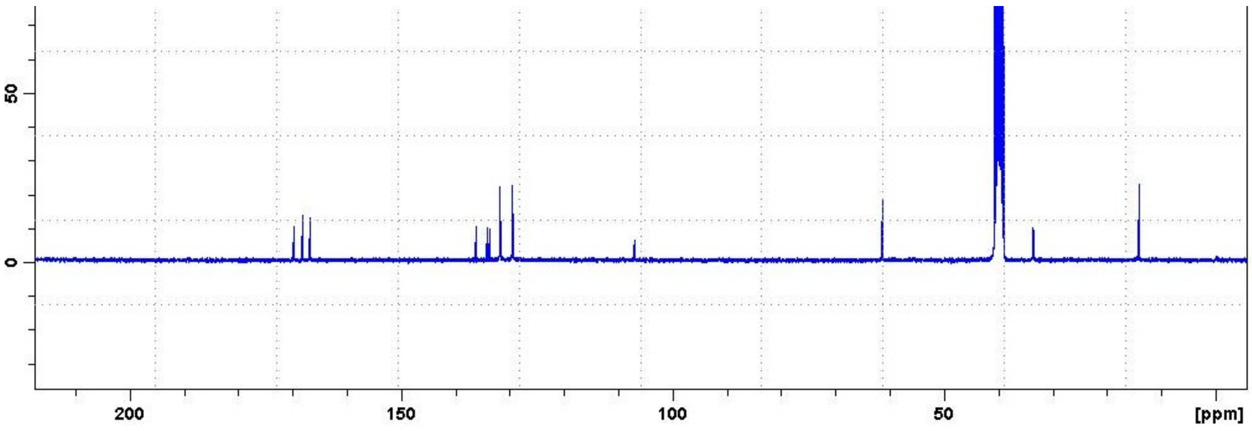

Ethyl [2-(benzylamino)-1,3-thiazol-4-yl]acetate (41)

^{1}H NMR of **41**

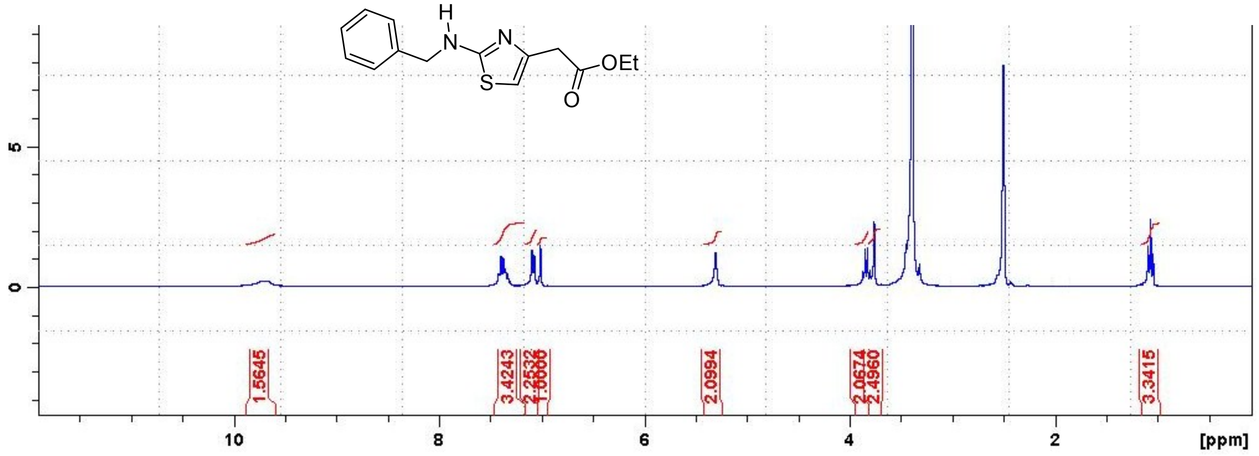

^{13}C NMR of **41**

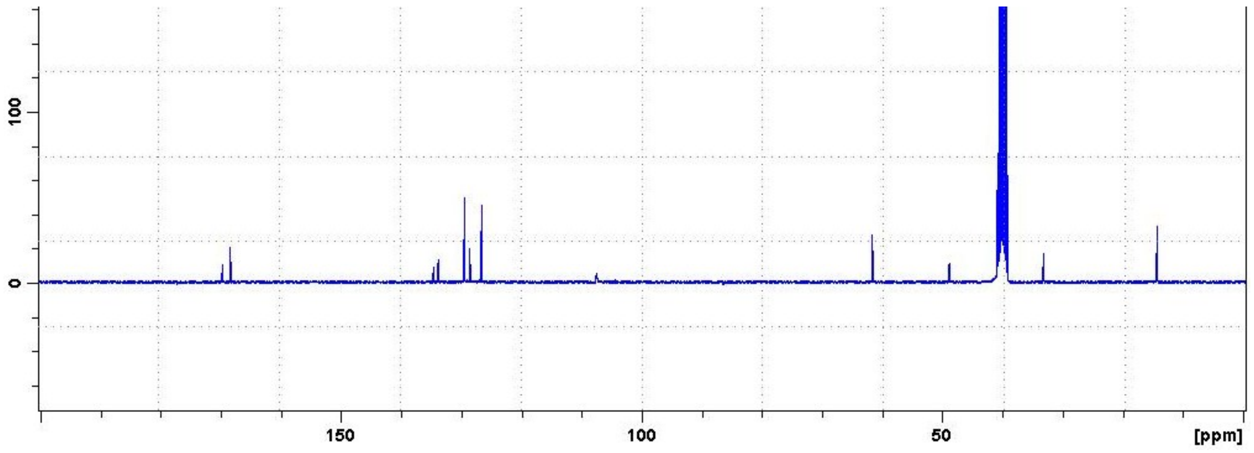

Ethyl {2-[(4-chlorophenyl)amino]-1,3-thiazol-4-yl}acetate (42)

^{1}H NMR of **42**

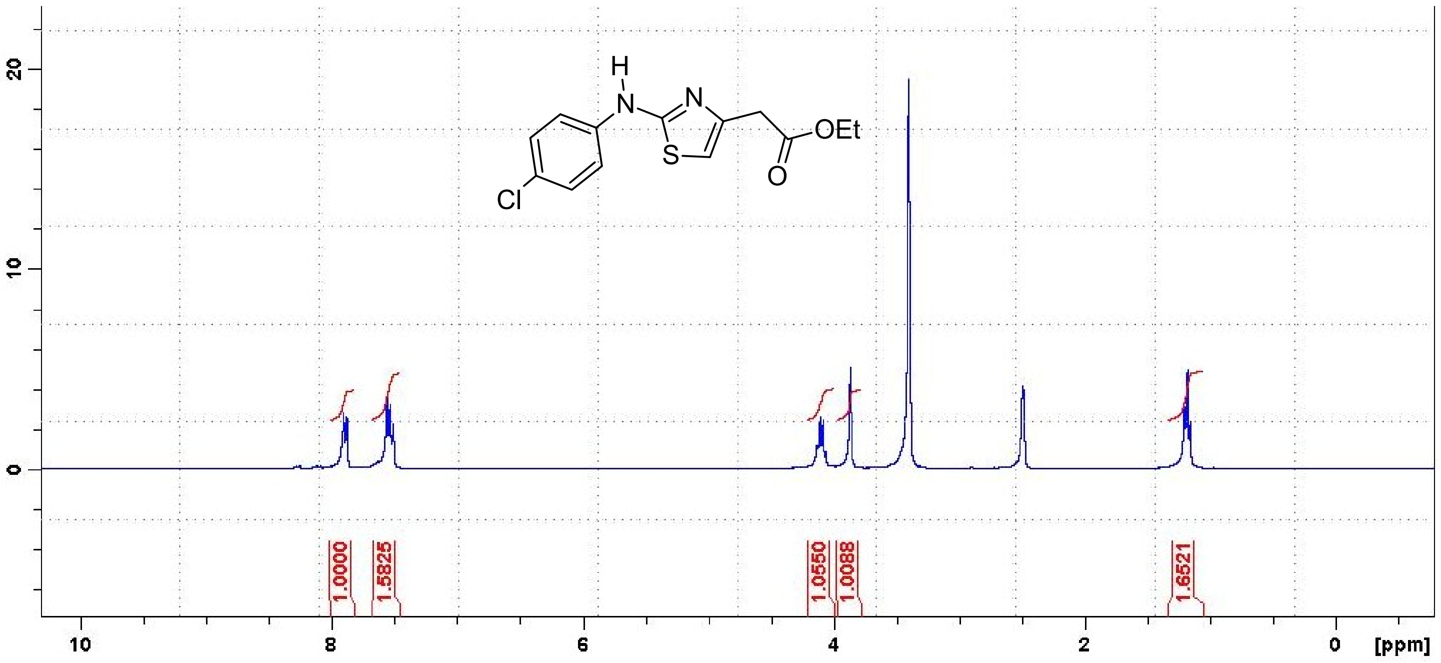

^{13}C NMR of **42**

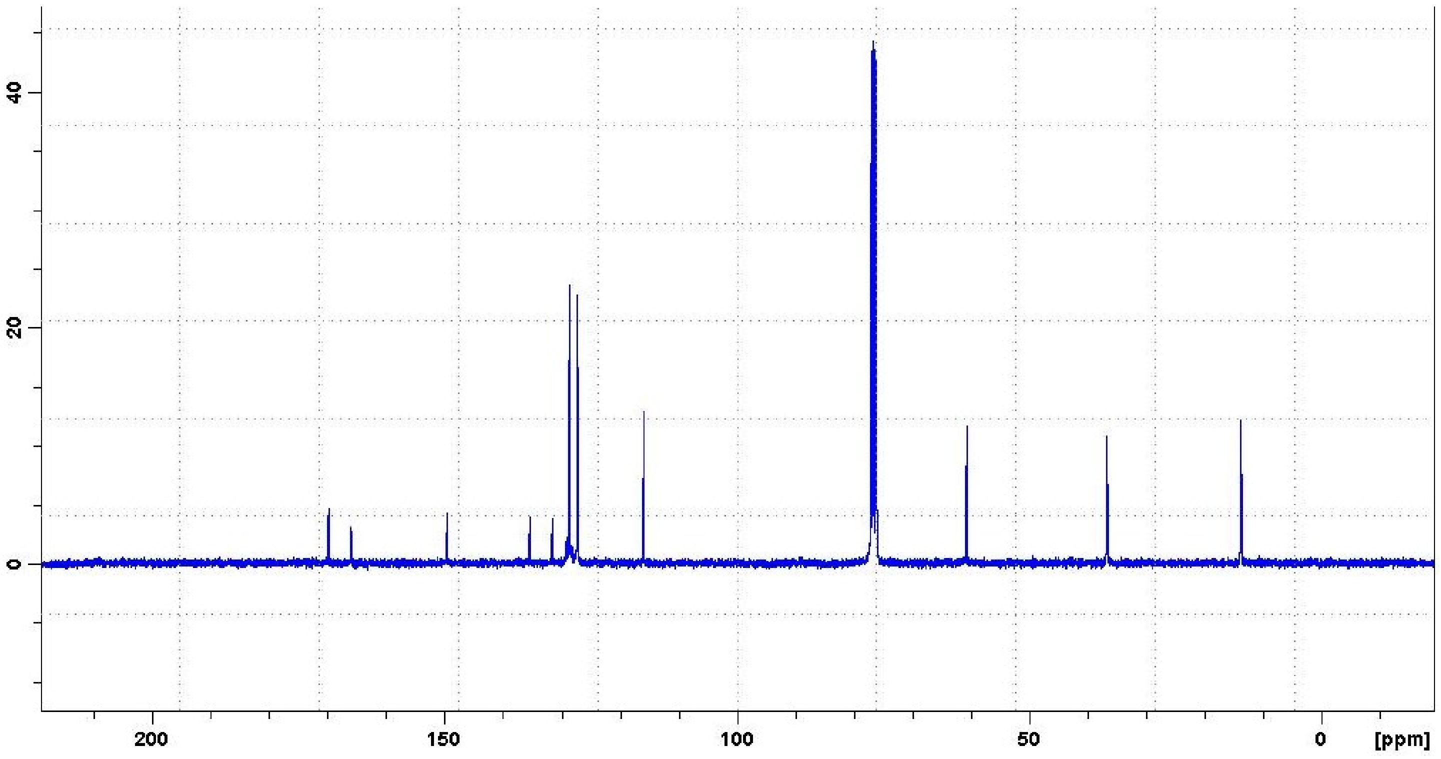

Ethyl (2-{[4-(trifluoromethyl)phenyl]amino}-1,3-thiazol-4-yl)acetate (43)

^{1}H NMR of **43**

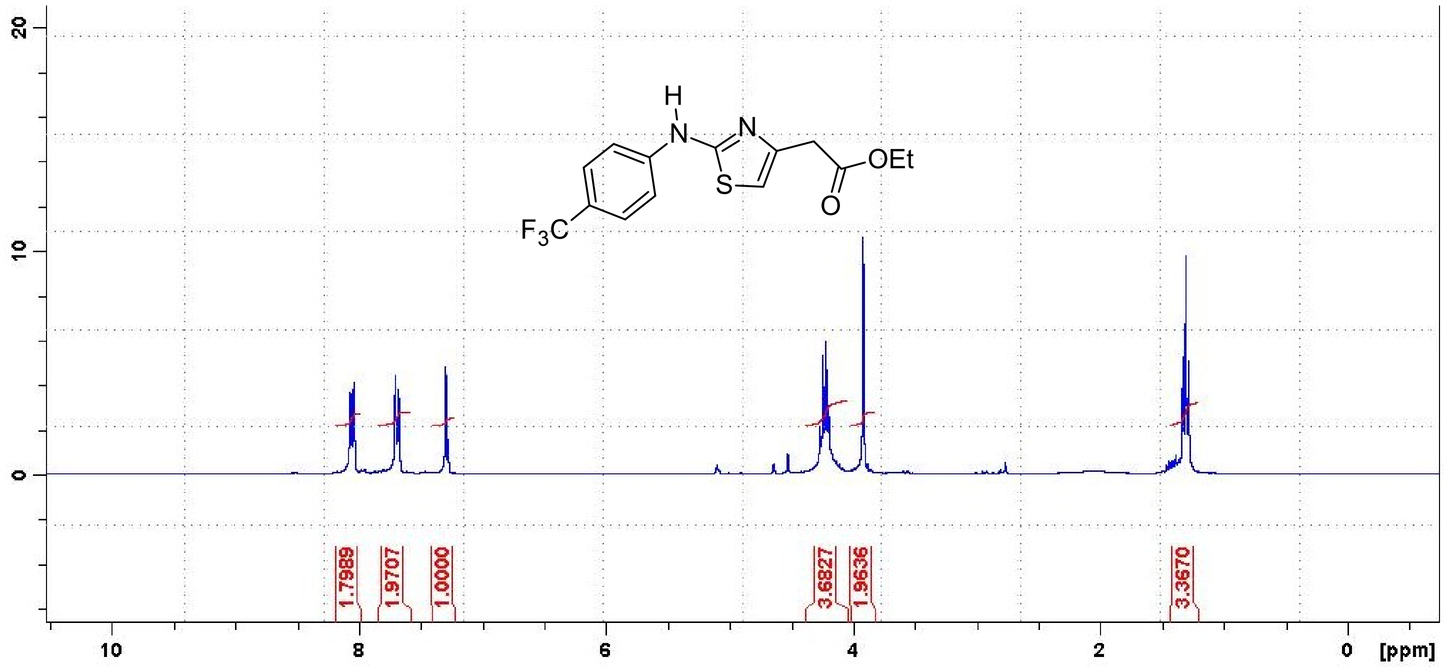

^{13}C NMR of **43**

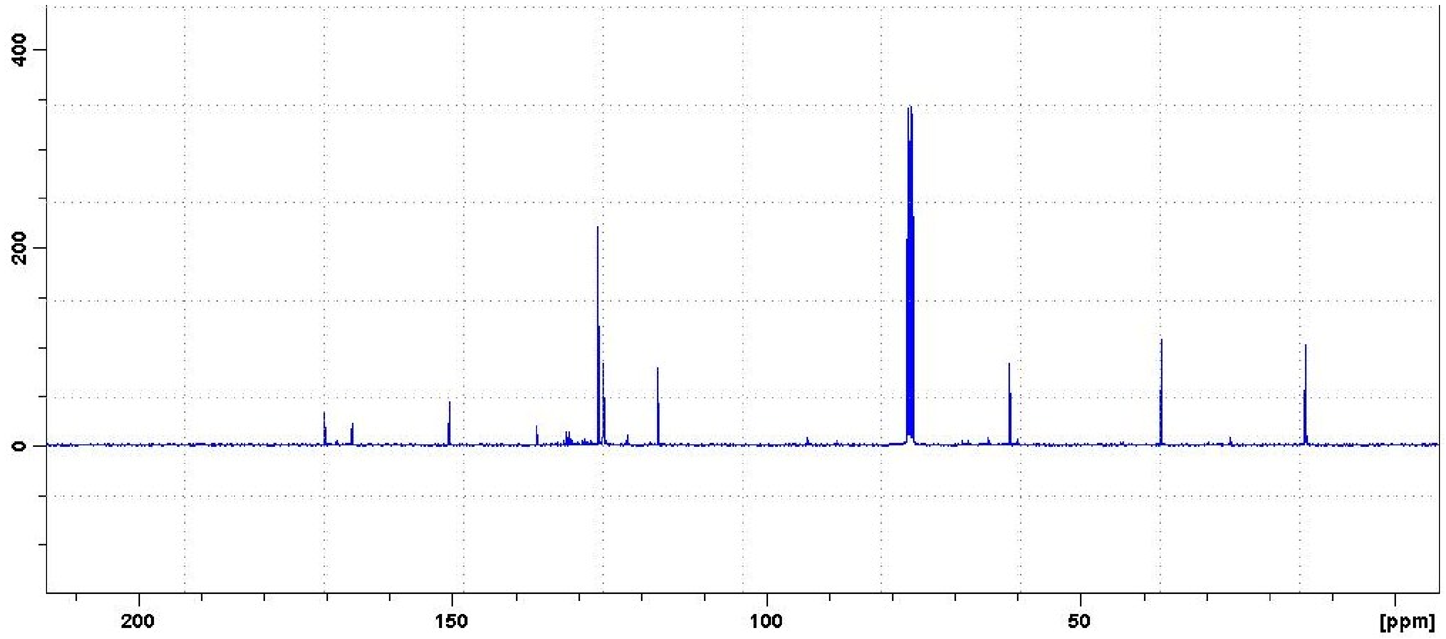

Supplement: Supplementary file 1 [file RA-007-C7RA05993K-s001.pdf]
